# Supplementary material for: Rationally designed laterally-condensed-catalysts deliver robust activity and selectivity for ethylene production in acetylene hydrogenation
Source: Nat Commun. 2024 Dec 10;15:10660. doi: 10.1038/s41467-024-54784-z (PMC11632098; doi:10.1038/s41467-024-54784-z)
Supplement: Supplementary file 1 — Supplementary Information [file 41467_2024_54784_MOESM1_ESM.pdf]

Rationally Designed Laterally-Condensed-Catalysts Deliver Robust Activity and Selectivity for Ethylene Production in Acetylene Hydrogenation

Zehua Li[1,10], Eylül Öztuna[1,2,10], Katarzyna Skorupska[1,10,*], Olga V. Vinogradova[3], Afshan Jamshaid[4], Alexander Steigert[5], Christian Rohner[1], Maria Dimitrakopoulou[1], Mauricio J. Prieto[4], Christian Kunkel[3], Matus Stredansky[1], Pierre Kube[1], Michael Götte[5], Alexandra M. Dudzinski[3], Frank Girgsdies[1], Sabine Wrabetz[1], Wiebke Frandsen[4], Raoul Blume[1,6], Patrick Zeller[1,2], Martin Muske[5], Daniel Delgado[1], Shan Jiang[1], Franz-Philipp Schmidt[1], Tobias Köhler[5], Manuela Arztmann[5], Anna Efimenko[7], Johannes Frisch[7], Tathiana M. Kokumai[7], Raul Garcia-Diez[7], Marcus Bär[7,8,9], Adnan Hammud[1], Jutta Kröhnert[1], Annette Trunschke[1], Christoph Scheurer[3], Thomas Schmidt[4], Thomas Lunkenbein[1], Daniel Amkreutz[5], Helmut Kuhlenbeck[4], Vanessa J. Bukas[3], Axel Knop-Gericke[1,6], Rutger Schlatmann[5], Karsten Reuter[3], Beatriz Roldan Cuenya[4], Robert Schlögl[1,*]

[1] Department of Inorganic Chemistry, Fritz-Haber Institute of the Max Planck Society, Berlin, Germany
[2] Bessy II, Helmholtz-Zentrum Berlin für Materialien und Energie GmbH, Berlin, Germany
[3] Theory Department, Fritz-Haber Institute of the Max Planck Society, Berlin, Germany
[4] Department of Interface Science, Fritz-Haber Institute of the Max Planck Society, Berlin, Germany
[5] PVcomB, Helmholtz-Zentrum Berlin für Materialien und Energie GmbH, Berlin, Germany
[6] Max-Planck-Institute for Chemical Energy Conversion, Mülheim an der Ruhr, Germany
[7] Department Interface Design, Helmholtz-Zentrum Berlin für Materialien und Energie GmbH, Berlin, Germany
[8] Helmholtz Institute Erlangen-Nürnberg for Renewable Energy (HI ERN), Berlin, Germany
[9] Department of Chemistry and Pharmacy, Friedrich-Alexander-Universität Erlangen-Nürnberg (FAU), Erlangen, Germany
[10] These authors contributed equally

* **Corresponding authors**
Katarzyna Skorupska (skorupska@fhi-berlin.mpg.de)
Robert Schlögl (rs01@fhi-berlin.mpg.de)

Table of Contents

Supplementary Note 1: 3
Supplementary Note 2: 5
Supplementary Note 3: 6
Supplementary Note 4: 7
Supplementary Note 5: 9
Supplementary Note 6: 11
Supplementary Note 7: 13
Supplementary Note 8: 16
Supplementary Note 9: 17
Supplementary Figures 1-32 19
Supplementary Tables 1-9 54
Supplementary Sample Table 68
Supplementary Method for DoE 75
Supplementary References 83

Supplementary Notes

Supplementary Note 1:

The carbon management process

The process must not be conducted at one location. Hydrogen can be brought from distant sources. Water must be desalinated and purified for electrolysis. The process can only work economically if the required energy is very low-cost. This is compatible in world locations where direct air capture (DAC) processes can be performed. It is, however highly advantageous to use as much as possible concentrated carbon emission sources for closing the carbon loop. The likely largest source for this will be waste composed of municipal waste, discarded organic materials from buildings and furniture and sludges from waste water treatment and biotechnology processes. It is envisaged that these carbon streams can be converted into torrefied compacted materials that can be transported to locations of low-cost energy where they might be gasified to syngas ideally combined with a CO_2 stream from DAC. Such a concept of relocating large-scale industrial processes from the traditional industrial centres to non-industrialized or largely inhabitable areas helps re-distribution the economic advantages of the energy transition in the world. It follows the same logic that created the present industrial centres in locations where cheap energy (coal, hydro-electricity) was available in the past. The reverse is also possible where liquid sunshine (derivatives of hydrogen) will be brought to locations where wastes are produced (large cities).

The economics of the process relies on the primary value of the molecular products. They are carbon neutral as can be verified directly at the production site and requires no complicated balancing system. In addition, service charges will be collected for removing CO_2 from the atmosphere. Part of these is the present CO_2 price that exists already today and will continue to be introduced. Another income is generated from treating and removing the waste being already today a sizable industry. This will have to change its products and also the waste separation and collection processes will partly have to be adapted. Despite of all these forms of income generated, the sales prize of the molecular products will likely be substantially higher than that of current fossil products of the same chemical structure. It is way too early to estimate the cost structure of such a process but it is evident that a substantial incentive not to waste the products made from these green molecules will result from their prizes. A veritable international transition strategy and change management will have to be put in place for manging such transition. All this is, however, inevitable if mankind takes it serious to defossilize their operations and to introduce circular economies for all relevant material flows of which carbon will remain likely the one with the largest volume.

A drawback in the non-oxidative carbon formation at desired “low” temperatures of about 1300K is the co-generation of molecular precursors, namely acetylene and ethylene as demonstrated in the Hüls process. The large scale of intended carbon deposition precludes isolation of acetylene for other potential important uses, but demands immediate selective hydrogenation to ethylene, being a safe and hydrogen-lean valuable product.

In concentrated gas streams, this exothermic reaction (ΔG_{398} = -129,85 kJ/mol) is much more demanding than the thermodynamically preferred full hydrogenation (ΔG_{398} = -218,72 kJ/mol. Consequently, the equilibrium constant $\ln K_{eq}$ at 398 K is 28 times larger for full- than

for semi-hydrogenation. In addition, the formation of polymer/carbon species (for $C_{18}H_{36}$ model polymer ΔG_{398} = -529,48 kJ/mol) must be supressed. The seemingly facile hydrogenation of concentrated streams of acetylene becomes so a highly demanding process with substantial safety issues attached if the continuous hydrogenation is interrupted for unexpected catalyst deactivation, and the acetylene stream reaches downstream devices. The safe solution of using excessively high temperatures for carbon formation is to be avoided for the energy economy of such large-scale process.

Supplementary Note 2:

Theory studies

Fig. 3a demonstrates the mechanistic role of subsurface C in the catalysis. This simplified picture[1,2] assumes two key reaction intermediates: the adsorbed acetylene (C_2H_2*) and ethylene (C_2H_4*) molecules. Weaker C_2H_4* binding suggests faster desorption of this intermediate and thus a higher selectivity towards partial hydrogenation. On the other hand, stronger C_2H_2* binding suggests faster acetylene activation from the gas phase and thus a higher overall turnover rate. The binding strengths of these two adsorbates though are not independent, i.e. they scale with one another, so that the resulting process is limited by a trade-off between catalytic activity and selectivity. This trade-off is controlled by changing the binding properties of the catalyst surface. Using DFT-computed heats of adsorption, **Fig. 3a** shows how the increasing concentration of subsurface C weakens adsorbate binding at Pd(111) so that the surface is less active, but more selective. This effect is consistent with a downshift of the Pd d-band center (cf. **Supplementary Fig. 2**) and qualitatively similar to that of alloying with Ag in the industrial catalyst.

Both experiment[3] and our DFT simulations agree that C favors octahedral sites within the first interstitial Pd(111) layer. The qualitative mechanism describing the effect of interstitial carbon atoms is not unique to the (111) facet surface. Specifically, **Supplementary Fig. 3** shows that subsurface carbon at a Pd(100) facet produces a similar qualitative scaling relationship between acetylene and ethylene adsorption energies.

Compared to carbon atoms, the presence of interstitial hydrogen has a much smaller effect on predicted adsorption energies as seen in **Supplementary Fig. 4**.

There is a clear downward shift in d-band center with increasing number of carbon atoms in the first interstitial layer. This effect has previously been reported in the literature[4] for a 1/4 ML coverage of Pd(111) in the subsurface. Conversely, hydrogen in same coverages does not noticeably affect the d-band center (**Supplementary Fig. 5**).

Supplementary Note 3:

Model LCC system with and without deposited carbon: Determination of electronic and morphological properties (XPS, SEM, STM)

In **Supplementary Fig. 7f** we studied the Pd:C LCC of approximately 2.5 nm by cross sectional analysis via STEM (Scanning Transmission Electron Microscopy). The sample was prepared by focused ion beam (FIB) and analyzed in STEM oriented to 101 zone axis direction of the Si substrate to resolve the interfaces sharply. **Supplementary Fig. 7g** shows Energy-Dispersive X-ray spectroscopy (EDX) elemental maps of Si (light blue), O (red), C (dark blue) and Pd (yellow) and their corresponding elemental profiles extracted from the EDX maps from top to bottom, integrated in horizontal direction. The dark blue and yellow peak in the profiles (see arrow in **Supplementary Fig. 7g**, right) point out the C and Pd elemental distribution across the Pd:C LCC.

Supplementary Fig. 8 illustrates the effect of annealing on the thin film catalysts (160°C for 30 min). Panels (**a-d**) show the effect for a film with a 0.4 nm thick interlayer between the SiO_2 and the Pd film [Si(100) – ~20 nm SiO_2 – ~0.4 nm C – ~3 nm Pd] and panels (**e-h**) the effect for a thin film catalyst without the C interlayer [Si(100) – ~20 nm SiO_2 – ~3 nm Pd]. The Si 2*p* intensity increases upon annealing due to dewetting of the layers upon more SiO_2 (or C/SiO_2) area is exposed after annealing and therefore the Si 2*p* becomes more intense. For the C 1*s*, the level at 283.9 eV gains intensity. This peak is due to carbon in Pd and consequently, the carbon-related Pd 3*d* level at 335.5 eV grows relative to the metal level at 335 eV**.** This means that part of the carbon from the interlayer diffuses into the palladium layer.

Supplementary Fig. 9 compares the spectra of a freshly prepared LCC Pd catalyst with spectra obtained after running the reaction for 20 hours. Panels (**a-d**) show the effect for a film with a 0.4 nm thick interlayer between the SiO_2 and the Pd film [Si(100) – ~20 nm SiO_2 – ~0.4 nm C – ~3 nm Pd] and panels (**e-h**) the effect for the thin film catalyst without the C interlayer [Si(100) – ~20 nm SiO_2 – ~3 nm Pd]. The two films behave very differently. After the reaction, the Si 2*p* level is strongly damped for the film with the C interlayer, which is due to the formation of a thick carbon layer. For the film without the C interlayer (panels **e-h**) the situation is different. Here much less carbon is formed. Consequently, the SiO_2 is not protected from interaction with the reaction gas, which reduces the oxide it is clear from the appearance of the small peak at low binding energy [98.53 eV] and the increased width of the main Si 2*p* level**.**

Supplementary Note 4:

Experiments to identify a suitable support and to verify the chemical nature of the active Pd species.

We verified that Si itself is not a suitable support. It occurs that Pd deposition results in smooth and dense films (**Supplementary Fig. 11a**). These films are independent of thickness catalytically fully inactive (**Supplementary Fig. 12**). Elemental and structural analysis of the cross section (**Supplementary Fig. 11b**) revealed a nm thick layer of Si penetrating through the Pd forming a dense SiO_2 film and the formation of Pd silicide. Attempts to disintegrate the inorganic overlayer by excessive hydrogen in the feed were unsuccessful indicating the possibility that SiO_2 can form a stable functional interface if we place it on the opposite side of the Pd.

In **Supplementary Fig. 11a**, a HRTEM cross-section micrograph shows a homogeneous thin film with a thickness of approximately 4 nm deposited on a Si wafer oriented at zone axis [110] (upper inset). To protect the integrity of the film during ion beam cutting a thin layer of carbon is applied. As seen from the image, the film is closed, homogeneous and polycrystalline. The local information obtained from the Fast Fourier Transform (FFT) diffraction pattern (lower inset in **Supplementary Fig. 11a**) reveals lattice planes which denote the formation of a palladium silicide phase Pd_xSi_y. The obtained reflections in circles highlighted in yellow, light blue and green colour indicate the presence of three lattice planes, which correspond to a lattice spacing of 2.451Å, 2.223Å, and 1.6067Å, respectively. Given that these crystal planes are common in various silicide phases (e.g. Pd_2Si, PdSi, Pd_3Si, Pd_9Si). The structural data were obtained from the Inorganic Crystal Structure Database (ICSD).[5] A detained phase analysis is not possible. The silicide formation clearly shows that Si has diffused from the silicon wafer to the film. Furthermore, silicon diffusion from the wafer to the top of the film was also observed. These phenomena are clearly presented in **Supplementary Fig. 11b** where the HAADF-STEM-EDS mapping image of the distribution of Si and Pd elements (inset: Pd distribution clearly evidencing its diffusion into the wafer as depicted by the yellow arrow) are displayed together with the corresponding EDS line scan profiles taken along the indicated magenta line. It is obvious that both Pd diffusion into the wafer (**Supplementary Fig. 11c**): EDS-B) and Si diffusion to the top of the thin film had occurred. Silicon on the top of the film is oxidized upon exposure to air, in turn, leading to the formation of silicon oxide (SiO_x).

Among the Pd films with nominal thicknesses of ~1 nm, ~3 nm, and ~11 nm deposited on silicon wafer, only the ~11 nm Pd film exhibits spectra of metallic nature. The palladium signal around 337 eV shows up in the Pd 3*d* region at RT for thinner palladium layers (~1 and ~3 nm) (**Supplementary Fig. 13b**). It is assigned as Pd silicide as justified with Pd L_3-edge in **Supplementary Fig. 14a.** Since there are several possible Pd silicides such as PdSi, Pd_2Si, Pd_3Si, and Pd_4Si and they all have slightly different binding energies in the Pd 3*d* spectrum, it is hard to identify the exact species formed. Nevertheless, the binding energy of the peak at 336.8 eV indicates that the dominating silicide is Pd_2Si.[6] For the ~11 nm thick Pd, heating at 100 °C in 0.1 mbar H_2 (also in 0.1 mbar O_2, not shown here) already triggers Pd silicide formation within minutes (**Supplementary Fig. 13c** and **d**).

Supplementary Fig. 13b and **d** show the Pd $3d_{5/2}$ spectrum for the ~11nm sample deconvoluted into the two peaks. The lower binding energy peak at 335.0 eV arises from

metallic Pd and the second peak at 335.5 eV is assigned to Pd with C dissolved in the Pd lattice (abbreviated as Pd:C)[7]. As the temperature increases (**Supplementary Fig. 13d**, ~11 nm Pd) in H_2, the Pd 3$d_{5/2}$ peak shifts to higher binding energies. At 100°C, the peak can be deconvoluted into two components at 336.3 and 336.8 eV, indicating a mixture of Pd_3Si and Pd_2Si[6]. When the temperature is increased further, the shift settles around 337 eV, indicating only Pd_2Si to be present[5]. The valence band spectra of ~11 nm Pd (**Supplementary Fig. 13c**) measured in UHV, RT and at elevated temperatures during in-situ heating experiments in 0.1 mbar H_2 are consistent with these phase transformations. The valence band of ~11 nm Pd in the as-prepared film shows a metallic Pd Fermi edge and two bands around 8 and 12 eV. They coincide with the one of the bare silicon oxide covered wafer, depicted as the blue curve in **Supplementary Fig. 13c**. The bands around 3, 8, and 12 eV belong to Si 2*p*-Si 2*p*, O 2*p*-O 2*p*, and Si 3*p*-O 2*p*, respectively[8]. This indicates that the film is not closed. Heating the sample above 100°C transforms the metallic Fermi edge into the Pd silicide valence band structure, in agreement with the Pd 3$d_{5/2}$ spectra[9]. For thinner layers of Pd (~1, ~3 nm), Pd silicide valence band features are observed already in UHV and RT (**Supplementary Fig. 13a**). The peaks around the Fermi edge and at 3 eV are formed by states resulting from the combination of Si 2*p* and Pd 4*d* bands. Moreover the peaks at 5 and 10 eV arise from Si 3*p* and 3*s* states[10].

Pd thicknesses of ~1 and ~3 nm resulted in non-metallic nanostructures (**Supplementary Fig. 13a, b**) and ~11 nm were required to deposit metallic Pd. From **Supplementary Fig. 13c** and **d** it occurs that even these Pd structures are highly reactive transforming under hydrogen into Pd compounds at mild reaction conditions. These observations underline the intended nonequilibrium character of sputtered Pd nanostructures.

As a consequence, the system containing no buffer layer (here shown for Si − ~11 nm Pd) shows neglectable catalytical activity at 125°C for acetylene hydrogenation as it is visible in **Supplementary Fig. 12a**. The remaining low activity (~ 6% in conversion) origins from the neglectable Pd contribution at 100°C (**Supplementary Fig. 13c, d**), while complete inactivity is found for Si − ~3 nm Pd (**Supplementary Fig. 12b**) due to the palladium silicide formation already at room temperature (**Supplementary Fig. 13a** and **b**).

We then reverted to Si wafers with pre-synthesized 20 nm thick SiO_2 and determined the nominal deposition thickness for metallic Pd. The target was to obtain the highest possible density of metallic Pd would result. Relevant data are reported in **Fig. 4**.

Supplementary Note 5:

Determination of electronic properties of XPS/NEXAFS spectra

The Pd 3$d_{5/2}$ shift of 337 eV (**Supplementary Fig. 13**) is characteristic of both Pd silicide in line with the conditions of hydrogen and elevated temperatures but unexpected from the overall mild conditions or of PdO in line with decomposing silicon oxides.

The strong overlap of Pd 3*p* XPS lines with O 1*s* lines renders the discrimination challenging. **Supplementary Fig. 15** and **16** evidence that we deal here with silicides and not with oxides. We further eliminated the idea that a Pd silicide or a Pd oxide may be formed at the functional interface of active nanostructures. We applied the unconventional Pd L_3 NEXAFS spectrum[11] with its high kinetic energy of 3.17 keV to probe the sub-surface volume (3-5 nm). As shown in **Supplementary Fig. 17** the spectrum of Pd on a functioning SiO_2 buffer layer (Pd metal by Pd 3*d* line) is that of Pd metal and not of a silicide which forms throughout the Pd layer (**Supplementary Fig. 11**) if the buffer layer is absent. The spectrum indicates further the absence of a hydride in the as measured state.

It cannot be excluded a priori that Pd oxides may play a role in the composition of LCC catalysts. The simple exclusion by photoemission is hampered by the strong overlap of O 1*s* lines with the Pd 3*p* spectrum. In the Pd 3p/O 1*s* spectra of Si − ~1nm Pd, Si − ~3nm Pd, and Si − ~11nm Pd samples, the Pd 3$p_{3/2}$ signals shown in **Supplementary Fig. 15b** are invisible due to the overlapping O 1*s* peaks of SiO_2 near 532 eV[12]. Reference experiments with a Pd foil allowed to locate the O1*s* features of PdO as shown in **Supplementary Fig. 15a**. A Pd foil is cleaned by Ar+ sputtering for 30 min, followed by in-situ heating the foil up to 600°C in 0.1 mbar O_2. **Supplementary Fig. 15a** shows the Pd 3*p*/O 1*s* acquired for the Pd foil after sputter cleaning, at 400°C, and 600°C in O_2. After the cleaning, only a single Pd 3$p_{3/2}$ peak is observed at 532.1 eV assigned to metallic Pd, accompanied by its plasmon loss peaks. At 400°C in 0.1 mbar O_2, bulk and surface Pd oxides are formed, with peaks emerging at 529.0 and 529.7 eV. A third peak at 530.9 eV in the O 1*s* region is also included in the fitting, which originates from the adsorbed O layer[13,14]. As the temperature is raised further to 600°C in O_2, bulk PdO is decomposed and surface Pd oxide peaks remain. Since the Pd 3*p*/O 1*s* spectra of the film sample Si − 20 nm SiO_2 – 3 nm Pd also displayed in **Supplementary Fig. 15a** match with that of the metallic Pd spectrum, one can eliminate the presence of Pd oxides.

A confirmation experiment that the spectra obtained after preparation and under acetylene synthesis conditions exhibit no contributions of oxide was made with the sample Si − 20 nm SiO_2 – 10 nm Pd. **Supplementary Fig. 16a** reveals the formation of surface oxide at 473 K in oxygen and of bulk oxide at 573 K. Whereas in the Pd 3*d* range oxide and silicide occur at similar binding energies the distinction between metallic and oxidic Pd is very clear in the spectral range displayed in **Supplementary Fig. 16b**.

Supplementary Fig. 14b shows a comparison of Pd L_3-edge NEXAFS spectra of a fresh Pd LCC (Si – 20 nm SiO_2 – 3 nm Pd) sample and Pd foil. The differences between the two spectra are the slight shift in the whiteline energy towards more positive values (0.2 eV) and the broadening of the whiteline peak in the Pd LCC sample as compared to that of the Pd foil. Both changes might result from a particle size effect, which was previously reported when comparing Pd L_3-edge spectra of a Pd foil with those from particles smaller than 10 nm[11]. Another explanation for the difference in the NEXAFS Pd L_3-edge spectra might be the different amounts of carbon incorporated into the Pd lattice. As it is demonstrated in the

manuscript, with increasing the amount of carbon in the Pd lattice, the whiteline shifts to higher energies and broadens (**Fig. 5b**). The absence of surface or bulk Pd oxide in the Pd LCC is already revealed by the XPS Pd 3*p*/O 1*s* core level fittings and comparison of the spectra with that of reference measurements performed with a Pd foil (**Supplementary Fig. 15a**). The absence of Pd oxide is also validated by the Pd L_3-edge spectrum of Pd LCC, since the whiteline of PdO has a significantly higher energy (by 1.3 eV) and the line shape is also more symmetric than that of metallic Pd, which is not the case for our Pd LCC sample[15].

Supplementary Fig. 17a shows comparison of Pd 3*d* spectra of fresh Si − 20 nm SiO_2 – 3 nm Pd, during in-situ acetylene hydrogenation, and the spent film after the in-situ measurements. The metallic Pd peak shifts slightly to lower binding energy when the reaction proceeds and afterwards, compared to the fresh sample. This might be resulting from the particle size increase during the reaction, which can lead to negative binding energy shifts in XPS[16-18].

Supplementary Note 6:

Microscopy on LCC Pd

Supplementary Fig. 20a shows the morphology of fresh LCC Pd and it is a homogeneous film with very condensed nanoparticles. **Supplementary Fig. 20b(i,ii)** displays HRTEM cross-sectional views of the fresh LCC with nominal thickness of 3 nm deposited on amorphous 20 nm SiO_2. The produced Pd thin film is homogeneous with an average thickness of ca. 4 nm. The thin film is polycrystalline evidenced by the ring FFT diffraction pattern of the lattice planes as shown in **Supplementary Fig. 20b(i)**. Palladium exhibits a cubic phase Fm-3m. The acquired results show the presence of two lattice planes, namely (111) and (200), which are assigned to interplanar spacing of 2.31 Å and 1.997 Å, respectively. The structural data were obtained from the Inorganic Crystal Structure Database (ICSD)[5].

The elemental distribution was observed using the HAADF-STEM-EDS map and line scanning profiles (**Supplementary Fig. 20b(iii)**). Based on the experimental results, the prepared LCC consists of Pd suggesting that no diffusion of either palladium or silicon occurred (**Fig. 4**), as opposed to the former Pd-Si system. This leads to the conclusion that the intermediate layer of SiO_2 between the Si wafer and the thin film Pd acts as a diffusion barrier layer.

The spent LCCs were also examined structurally and compositionally by means of TEM. In **Supplementary Fig. 20c(i)** displays the HRTEM cross-sectional micrographs of spent Si − 20 nm SiO_2 – 3 nm Pd. Thin layer Pd shows instability during the acetylene hydrogenation reaction, since the film is detached from the support resulting in regions where no Pd material is preserved on the substrate. In addition, the thickness of the film is inhomogeneous and increases after reaction compared to that of the fresh ones, with an average thickness of approximately 10 nm. However, the remaining thin film preserves both its phase and elemental properties. More analytically, the film phase is cubic Pd and the lattice spacing of 2.31 and 1.997 Å corresponds to the (111) and (220) lattice planes of cubic Pd, respectively. Based on the HAADF-STEM-EDS map and line scanning profiles for both samples as presented in **Supplementary Fig. 20c(iii)**, it is deduced that the LCC consists of Pd suggesting that no diffusion phenomena of either Pd or Si occurred, as already shown in the fresh samples. SEM images of the spent Pd based LCC after 3h, 10h and 18h TOS showing progressive agglomeration during the catalysis are presented in **Supplementary Fig. 21**.

LEEM, XPEEM and LEED on LCC Pd

We investigated the structure integrity of a 3 nm Pd supported on a 20 nm SiO_2/Si(100) substrate using low energy electron microscopy (LEEM), low energy electron diffraction (LEED) and X-ray photoemission electron microscopy (XPEEM). Sample transfer was performed under controlled atmosphere. **Supplementary Fig. 22a** shows a LEEM micrograph collected at room temperature of the Si − 20 nm SiO_2 − 3 nm Pd sample. As can be seen from the image, the surface presents a granulated structure thus suggesting the presence of a certain degree of roughness in the Pd film. The LEED pattern collected on the sample (see inset in **Supplementary Fig. 22a**) reveals the lack of two-dimensional ordering on the film, where a three-dimensional structuring of the film is anticipated. The dark feature located at the bottom at the structure corresponds to an occasional defect on the Pd film (not representative of the sample but included as a reference point).

Supplementary Fig. 22b shows an XPEEM image collected using the Pd 3*d* photoemission (see spectrum on **Supplementary Fig. 22c**) line at a photon energy of 405 eV. As can be seen, the distribution of Pd is homogeneous across the sample and no special domains are detected within the resolution of the microscope under the experimental conditions used (ca. 50 nm). The higher intensity in the centre of the XPEEM image corresponds to a homogeneous intensity profile of the focused X-ray beam.

Supplementary Note 7:

Catalytic activity, selectivity and stability of LCC Pd

In **Supplementary Fig. 23** the still significant loss of carbon to polymers for the LCC catalyst is partly explained by the high activity of the system. We have indications that with a modified pretreatment procedure avoiding PdH formation and filling the Pd correctly with carbon where the carbon balance may be substantially improved. We intend to perform the optimization by operando experimentation guided through DOE and are setting up technical infrastructure to do so.

As shown in **Supplementary Fig. 23e,f**, the mass loss and the TG-MS signal for CO_2 (m/z = 44) were compared for the as-prepared and the spent Pd LCC samples after 10h time on stream (TOS).The measurements were performed in synthetic air (21% O_2 in Ar). We attribute the initial mass increase recorded on both, the as-prepared and spent LCC catalysts in the temperature range 40-150°C to oxidation of the thin Pd film. While the as-prepared LCC shows no mass loss at higher temperatures, the mass loss of 0.01% up to 700°C is likely assigned to the decomposition and combustion of carbonaceous deposits on the spent LCC, in agreement with the MS signal for m/z = 44. The different maxima of the MS signal at 350°C, 500°C and 650°C indicate that different types of carbonaceous species are present on the surface. In agreement with the Raman spectra of the spent sample (**Supplementary Fig. 23c, d**), these species can be attributed to molecular carbonaceous species, deposited polyacetylene and perhaps to a minor extent to green oil[19]. The presence of graphitic carbon species was not detected. Temperature Programmed Desorption (TPD) performed in an Ar atmosphere on the spent Pd LCC sample (TOS = 10h) showed no signal for m/z = 27 (**Supplementary Fig. 23g**), which would elsewise be expected as a significant fragment in the mass spectra of adsorbed shorter hydrocarbons, like C2-C4. In contrast, the contribution of fragment 27 is much lower in the mass spectrum of green oil, which is composed of linear C12 – C30 hydrocarbons. Therefore, the absence of the m/z signal 27 suggests that the carbon deposits on the catalyst surface consist of heavy hydrocarbons or polymerized chains that can only be removed in an oxidizing atmosphere (**Supplementary Fig. 23e, f**).

We performed product detection using online mass spectrometry (**Supplementary Fig. 23b**). The following m/z intensities were measured: H_2 (m/z=2), N_2 (m/z=14 and 28), C_2H_2 (m/z=26), C_2H_4 (m/z=28), C_2H_6 (m/z=30), C_4H_6 (m/z=39, 54) C_4H_8 (m/z=41, 56), C_4H_{10} (m/z=43, 58). Other minor fragments/peaks were also found. As expected, heavier hydrocarbons were not detected in the gas phase. Higher hydrocarbons remain adsorbed on the catalyst surface, on the reactor walls and in the tubing. Raman spectroscopy was used to analyze the nature of the deposits on the spent catalyst. The Raman spectrum of the as-prepared LCC is essentially free of features due to carbon, although a weak broad band close to 1600 cm^{-1} suggests the presence of minor graphitic impurities in the fresh catalyst (**Supplementary Fig. 23d**). Two strong peaks at ca. 1100 cm^{-1} and ca. 1500 cm^{-1} can be detected in the spent catalysts, which are attributed to the characteristic modes of carbon-carbon single bond and carbon-carbon double bond stretching vibrations, respectively, in adsorbed *trans*-polyacetylene[20]. The frequency of the C=C stretching mode depends on the structure and the conjugation length of the polymer[21]. C-H stretching vibrations are also observed at 2920 cm^{-1}. The coverage of the surface with polyacetylene is also in agreement with the thermal analysis in air (**Supplementary Fig. 23e, f**) as polyacetylene undergoes a phase transition and oxidative

degradation at temperatures above 300°C[22]. Images taken from the camera of the Raman microscope show also different light refraction for the sample after 49 and 260 hours TOS, suggesting the presence of different thicknesses of organic material on the surface of these two samples. As the composition of the organic films and therefore the refractive index is not precisely known, the color cannot be used to make an estimation of the film thickness. The Raman spectrum of an oily liquid ("green oil") recovered from the cell walls of the Raman cell in an operando acetylene hydrogenation experiment is shown in **Supplementary Fig. 23c**. The spectrum differs from the spectrum of the carbonaceous deposits on the used catalysts, so that it can be assumed that the organic layer on the used catalysts consist mainly of polyacetylene and not of the green oil.

The Si − 20 nm SiO_2 − 3 nm Pd was tested at both 150 and 80°C for long-time stability and was compared with Pd powder and foil catalysts. Both cases the LCC Pd has a very high conversion of 98% however deactivates with time on stream (**Supplementary Fig. 24a, b**). The lifetime of LCC Pd is longer at 150°C than 80°C. LCC Pd can still obtain a conversion of 30% after running for 180 hours at 150°C, while it deactivates at 30th hours when operated at 80°C. LCC Pd favors C2 coupling to form C_4H_{10} and higher hydrocarbons at 80°C as the C_4H_{10} selectivity is higher than that at 150°C (**Supplementary Fig. 24g**). At 150°C, the desorption of C_2H_4 and C_2H_6 on Pd surface is faster than that at 80°C, hence less C_2H_4 and C_2H_6 are coupled to form C_4H_{10}. Higher hydrocarbons or even oligomer and carbon deposits can block some active centers therefore deactivate the catalyst (see Supplementary Note 8). The deactivation could be also from the Pd aggregation during catalysis with time on stream as already shown in **Fig. 4**. Normally, there is formation phase before a selectivity-steady phase during acetylene hydrogenation. The beginning to the 60th hour around is the formation phase due to the LCC Pd still forming itself (**Supplementary Fig. 24a**). This period for Pd powder catalyst is from the beginning to the 30th hour (**Supplementary Fig. 24c**). Semi-hydrogenation reaction competes with full-hydrogenation reaction, but it dominates with time on stream in the formation phase, after which is the selectivity-steady phase. The selectivity to C_2H_4 can stabilize at 93% for LCC which is much higher than 80% for Pd powder catalyst (150°C) (**Supplementary Fig. 24f**). This is also observed at 80°C. Nealy no full-hydrogenation reaction was observed after 60th hour in LCC indicating a steady formation of C_2H_4 and few C_4H_{10} (**Supplementary Fig. 24a**). The full hydrogenation is still there even in selectivity-steady phase for Pd powder catalyst but with a low selectivity (**Supplementary Fig. 24c, d**). The long-time stability was compared between LCC Pd and Pd catalysts as shown in the productivity in **Supplementary Fig. 24e**. The productivity C_2H_4 is higher in Pd powder catalyst than LCC Pd in the early 40 hours, however less competitive later for long-time stability till 180 hours. The deactivation is faster in Pd powder than LCC Pd. One reason for this could be that the selectivity to higher hydrocarbons (≥C4) in powder catalyst is higher than LCC. C2 and C4 are probably more easily decoupled from Pd in LCC with less barrier.

The 3 nm, 100 nm and foil Pd were measured under acetylene hydrogenation using identical catalytic conditions. Due to the very high amount of Pd in the ~0.2 mm thick Pd foil and 100 nm thick Pd, they have high conversion (**Supplementary Fig. 25a**) but are less selective (**Supplementary Fig. 25b**) in comparison with the 3 nm Pd. However, they have a very close selectivity vs. conversion behavior (**Supplementary Fig. 25c**), which is much different when compared to Pd powder (**Supplementary Fig. 24f**).

In order to make the comparison more straightforward, the data are also presented in form of C_2H_4 productivity where the yield of C_2H_4 was normalized to mass of palladium in the

catalyst and the area of the catalyst. The 3 nm Pd on 20nm SiO_2 has much higher productivity by four magnitudes than the Pd foil and 100 nm Pd (**Supplementary Fig. 25d**). When normalized to the area of catalyst, the difference is minor between 3 nm Pd and 100 nm Pd but they have about twice productivity of the Pd foil (**Supplementary Fig. 25e**). Note that here Pd foil is with Pd on double side while either 3 nm or 100 nm Pd are single side coated.

In **Supplementary Fig. 25f**, the selectivity and activity of the LCCs Pd are further compared to reported monometallic Pd/Al_2O_3 and bimetallic PdGa and PdAg powder catalysts[23]. The above-mentioned Pd powder and Pd foil are also compared. More comparison at various experiment conditions is summarized in the **Supplementary Table 1**. The used literature data were obtained under different catalytic conditions (Pd loading, support, catalyst size, reaction temperature, gas feed) - therefore the comparison is quite challenging. In this paper, the productivity calculated from the catalytic data was used as a standard descriptor. As it can be observed in **Supplementary Fig. 25f**, **Table 1**, the studied LCCs Pd have higher productivity than reported other catalysts such as the Pd nano powder[24,25] and PdM (M: metal) on various supports[26-30]. The conventional catalyst, Pd/Al_2O_3 powder shows a pronounced activity, however, exhibits very low selectivity (black dot in **Supplementary Fig. 25f**). Enhancement of the selectivity for ethylene is obtained by adding a second metal for a bimetallic PdGa or PdAg systems[30]. From our represented comparison it is clear that the monometallic Pd in LCC reveals beneficial selectivity and activity (**Supplementary Fig. 25f**, **Table 1**). It is also obvious that thick Pd, e.g. Pd foil, 100nm Pd and high loading of palladium in powder catalyst does not necessarily constitute a prerequisite.

The turnover frequency (TOF, in s^{-1}) of LCC was calculated as the acetylene consumption rate per accessible Pd center. LCC Pd have a higher TOF than reported single atom Pd catalysts[31-33] (**Supplementary Fig. 25g**).

By comparing different catalyst pre-treatments we were able to gain insight into whether the source or the way we introduce carbon into the Pd LCC influences its activity or selectivity. In particular, different pre-treatments are expected to result in a different content of carbon incorporated into Pd or deposited on the Pd surface, which would either contribute to the stabilization of the material or to the blocking of active sites. Such different starting pre-catalysts conditions were then compared to draw conclusions on the optimum pre-catalyst composition and state. Here we observed that an improvement in the catalytic performance arises when the gas composition during the activation process is adjusted such as to maximize the Pd:C content (**Supplementary Fig. 26**).

Supplementary Note 8:

Volumetric adsorption and PM-IRAS spectroscopy

For the quantitative determination of the total accessible Pd surface sites, CO was used as the standard probe molecule at a standard adsorption temperature of 40°C. However, for the determination of the catalytically relevant Pd surface sites, the synthesis gas mixture (C_2H_2:H_2=1:10) was used as the probe molecule at 40°C.

Supplementary Fig. 28a shows the isotherms of adsorption of CO and the syngas mixture (C_2H_2:H_2=1:10) on Si − 20 nm SiO_2 − 3 nm Pd at 40°C. From these isotherms, the adsorption capacity can be read, which corresponds to the saturation concentration when the adsorbed amount remains almost constant with further increasing partial pressure. A slight increase in the adsorbed quantity after reaching saturation (monolayer) is due to multi-layer adsorption. This Pd film provides an adsorption capacity of about 70 $mmol_{CO}/g_{Pd}$ for CO and much less about 30 $mmol_{C2H2:H2}/g_{Pd}$ for the synthesis gas mixture. This observation clearly shows that only roughly half of the available Pd surface sites are also catalytically interesting. This trend was to be expected.

Supplementary Fig. 28c shows the CO adsorption capacity of the LCC Pd compared to the Pd powder catalyst and a commercial Pd foil, normalized to the total Pd amount of the respective sample. Here, the high catalytic potential of the LCC Pd becomes visible. The CO absorption capacity is many times higher than that of the powder catalyst or even the Pd foil. This fact can also be safely transferred to the synthesis gas mixture and thus confirms the measurably proven better catalytic performance of the LCC Pd compared to the reference materials.

In conclusions the LCC Pd has much higher amount of the accessible Pd than that of Pd powder and foil (**Supplementary Fig. 28c**). The accessible Pd in real reaction gas is lower than in CO (**Supplementary Fig. 28a**). The adsorption of C_2H_2:H_2 is reversible while the adsorption of CO is irreversible.

To better understand the deactivation of LCC Pd, CO and C_2H_2 adsorption was performed to determine the amount of accessible Pd surface sites on a fresh and a used film. **Supplementary Fig. 8d,e** shows very clearly that Pd surface sites are lost (blocked, altered, etc.) over the course of the reaction.

Supplementary Fig. 29 compares the adsorption of CO on a Pd foil (Goodfellow) and a Si − 20 nm SiO_2 − 3 nm Pd after pretreatment for one hour in 300 mbar H_2 at 398 K. The spectra were recorded at 313 K after treatment with CO at an equilibrium pressure of 322 mbar CO (LCC) and 84 mbar CO (foil) and subsequent evacuation. The spectrum of the foil shows CO adsorbed on-top at ca. 2054 cm^{-1}, bridge-bonded at 1928 cm^{-1} and at hollow sites at ca. 1822 cm^{-1}. The peak positions are at comparatively low energy, which suggests a strong binding of CO to a comparatively electron-rich Pd surface. However, due to the strong dependence of the peak positions on the degree of coverage and the polycrystalline nature of the foil, the latter statement is not very reliable and should be treated with caution.

Interestingly, only bridge-bonded CO is observed on the LCC. The formation of only this single type of configuration and the band positions between ca. 1960 and 1970 cm^{-1} indicate the presence of a defect-rich[34], and stepped surface[35] at which adsorption of CO in a bridged coordination on defects or over step edges dominates. The surface structure of the LCC is therefore clearly different from the foil.

Supplementary Note 9:

Design of Experiments (DoE)

We illustrate the robustness of the novel thin-film catalysts by analyzing how an additional extended heat treatment at 125°C for 16 hours affects their time-averaged selectivity. Hereby, we not only consider key factors controlling the initial deposition of the $Si(O_x)$ buffer layer and Pd films like the applied total pressure or the plasma or sputter power. Equally important for the catalyst design process are factors characterizing a synthetic conditioning step within the reactor like the presence or absence of carbon sources or the temperature profile during this conditioning.

The finally selected combined set of deposition and chemical synthesis control factors resulted in a 10-dimensional design of experiment (DoE) control factor space. Supplementary experimental sections E1,2 and section D1 extensively discuss these control factors. An efficient and robust screening design (**Supplementary Table 9**) was then derived over maximally varying settings over all control factors. It finally included 70 (10 batches), fully conditioned catalysts prepared from 35 uniquely deposited film. The design was computationally constructed as described in section D5, also incorporating the batch requirements of the parallel reactor.

The purpose of the designed experiment is to identify the small subset of control factors that significantly influences the catalytic stability of these samples (following the "sparsity-of-effects principle")[36]. Catalytic stability of each sample is here specifically judged by the proxy property $\bar{S}_2 - \bar{S}_1$, where $\bar{S}_1$ and $\bar{S}_2$ denote averages over ethylene selectivities (S_{C2H4}) measured in early and late TOS periods, respectively. For two selected samples, this is illustrated in **Supplementary Fig. 32 (i)**: The stability of the studied catalysts can vary strongly over time-on-stream and can be influenced by control factor settings. Such variations in catalytic observables are indicative of catalyst formation. The synthetic conditioning step within the reactor is thus deliberately included in the catalyst design process to gain further control over such processes. The conditions applied during the conditioning step could then also be varied significantly from regular TOS conditions, e.g. to target long-term stability objectives rather than regular catalytic conversion.

Such strong variations in catalytic observables are also needed for the ensuing statistical analysis of influential synthesis factors. Reassuringly, under the DoE experimental conditions, strong variations are observed over the whole dataset: Advantageously, all samples could be catalytically characterized in early and late TOS periods with observed acetylene conversions ranging around 33 to 100% (the latter seen in roughly 60 % of samples). All samples could therefore enter analysis and their average selectivities for ethylene then overall ranged around 1-85 %. The resulting variation in our target property, $\bar{S}_2 - \bar{S}_1$ is illustrated for all samples in **Supplementary Fig. 32 (ii)**. Based on this data, the statistical analysis of the designed experiment could be carried out by means of a factor screening model. Sections D2 and D3 describe this workflow in full detail. Encouragingly, predictions of this statistical model already show a reasonable correlation with measured data, see **Supplementary Fig. 32 (iii)**.

The model then pinpoints influential control factors by first assessing how changes in their settings linearly affect catalytic stability. This approach of screening for linear effects first is justified, as they likely present the strongest sources of variation for catalytic stability (as established in the empirical "hierarchy-of-effects principle"). The analysis finally yields a clear

indication of the most influential design factors “Sputter Power”, “Hydrogen” and “Acetylene” by uncovering their strong linear effects (largest deviation from dashed 0-line in **Supplementary Fig. 32 (iv)**). In brief, this indicates that the Pd film thickness as well as its exposure to different gas feed compositions during conditioning have a significant influence on the catalytic stability of the thin film catalysts. A more detailed discussion of the potential factor influences on the interface formation is provided in section D1. Following these strong variations with characterization studies along prototypical samples might elucidate the modes of action further.

We also found, that active pairwise interactions or quadratic effects lead to deviations from simple linear behavior. As a larger number (combinatorially n(n-1)/2 for n factors) of potential interactions exists, we here non-exhaustively test for the strongest ones, following the rationale described in section D3. The two most influential pairwise interactions “Sputter Power x Hydrogen” and “Sputter Power x Acetylene” have been included in the statistical model. Both describe situations in which the impact of the Pd film thickness on catalytic stability varies depending on the absence or presence of these gases in the conditioning feed.

Overall, this systematic, quantitative analysis reveals how catalytic stability can be influenced by single or jointly applied changes to in distinct factors. This included a first investigation into how catalytic behavior can be significantly influenced through a conditioning phase. This knowledge might also be used to provide relevant synthetic attack angles for improvements of the hitherto studied catalysts, and for further enquiry by use of targeted characterization studies.

On the design side, we also note, that our screening experiment was so far equipped to identify the most influential (linear) effects. As commonly encountered in screening designs it might thus fail to fully unravel the complexity of the underlying response surface, leaving some ambiguity about the full set of model terms that govern the process, see also sections D3 and D6 for further discussion. Adaptive DoE steps could now be applied to elucidate the situation further, to explore (e.g. to avoid shortcomings observed in the screening), to extend (e.g. by dopants) the catalyst phase space and to identify ideal catalyst / TOS conditions.

Supplementary Figures 1-32

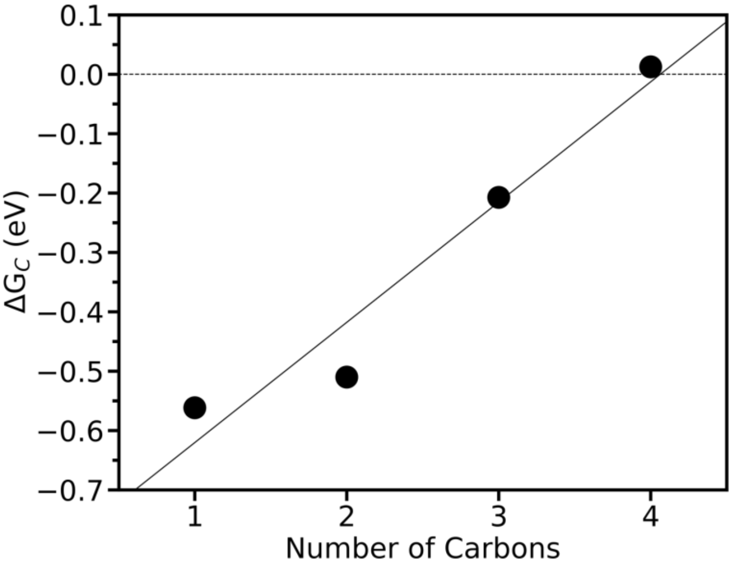

Supplementary Fig. 1 | Per-carbon change in free energy as a function of the total number of sub-surface carbon atoms simulated in a (3x3) surface unit cell of Pd(111) using DFT. The free energy of carbon is referenced to gas-phase acetylene. The solid line is a linear fit across all data points and the dashed line indicates zero free-energy, beyond which further carbon intercalation becomes endothermic.

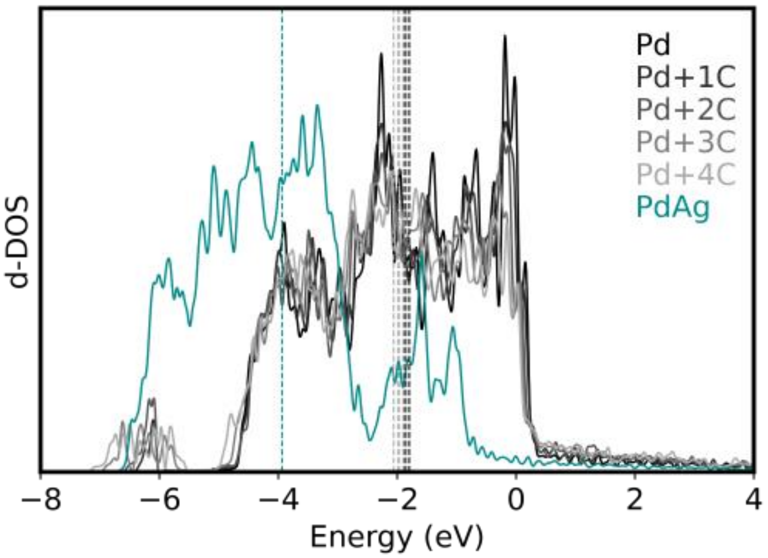

Supplementary Fig. 2 | Calculated projected density of states of the *d*-orbital for Pd(111) and PdAg(111) with varying numbers of carbon atoms in the first interstitial layer of a 3x3x4 unit cell. All energies are plotted relative to the Fermi energy of the corresponding surface. Dotted lines mark the d-band centers.

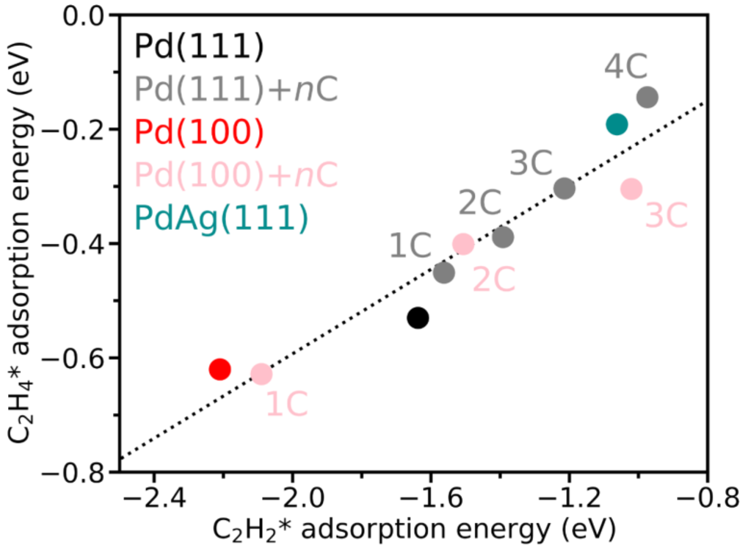

Supplementary Fig. 3 | Scaling relation between DFT-calculated adsorption energies of C_2H_2 and C_2H_4, supplemented with data for a clean Pd(100) surface (red) with an increasing concentration of subsurface C atoms within the first interstitial layer (pink) of a 3x3x4 unit cell. The dotted line is a linear fit to guide the eye.

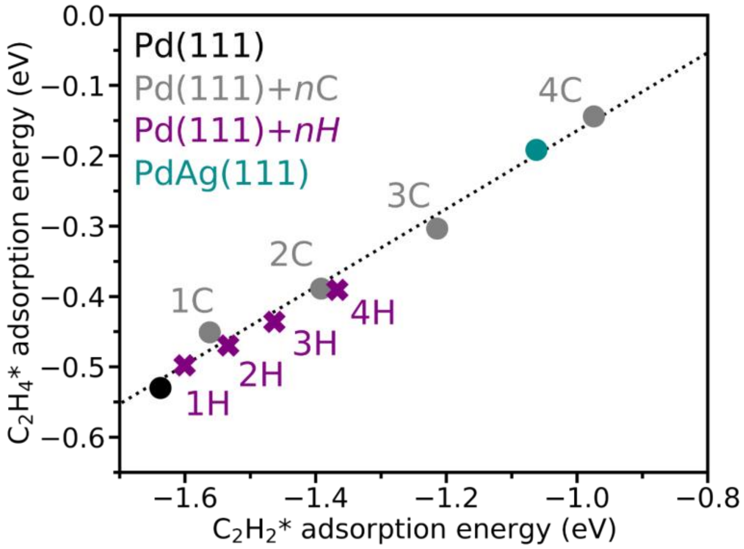

Supplementary Fig. 4 | Scaling relation between DFT-calculated adsorption energies of C_2H_2 and C_2H_4 on a clean Pd(111) surface (black) with an increasing concentration of subsurface C atoms (grey) or H atoms (purple) within the first interstitial layer of a 3x3x4 unit cell. The dotted line is a linear fit to guide the eye.

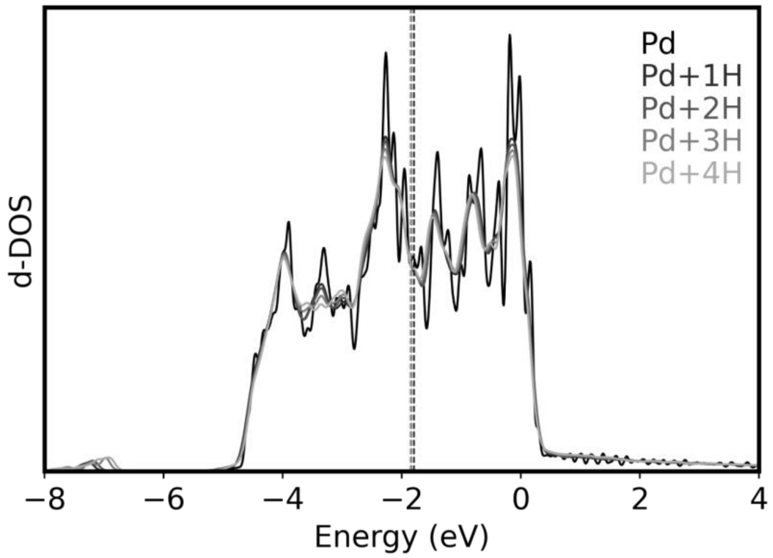

Supplementary Fig. 5 | Calculated projected density of states of the d-orbital for Pd(111) with a varying number of hydrogen atoms in the first interstitial layer of a 3x3x4 unit cell. All energies are plotted relative to the Fermi energy of the corresponding surface. Dotted lines mark the d-band centers.

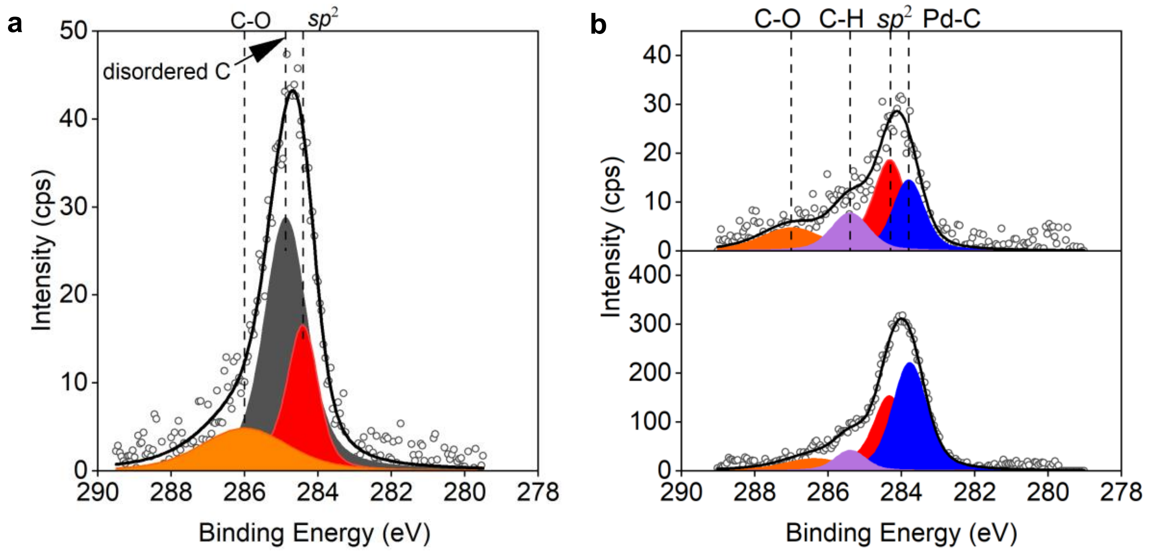

Supplementary Fig. 6 | **a** XPS C 1*s* spectrum of Si(100) – ~20 nm SiO_2 – ~0.4 nm C and **b** C 1*s* spectrum of fresh Si(100) – ~20 nm SiO_2 – ~0.4 nm C – ~3 nm Pd (top) and after annealing at 150°C for 30min in UHV (2 x 10^{-9} mbar) (bottom). XPS employing monochromatized $Al_{k\alpha}$ radiation and a Specs electron energy analyzer (Phoibos 200).

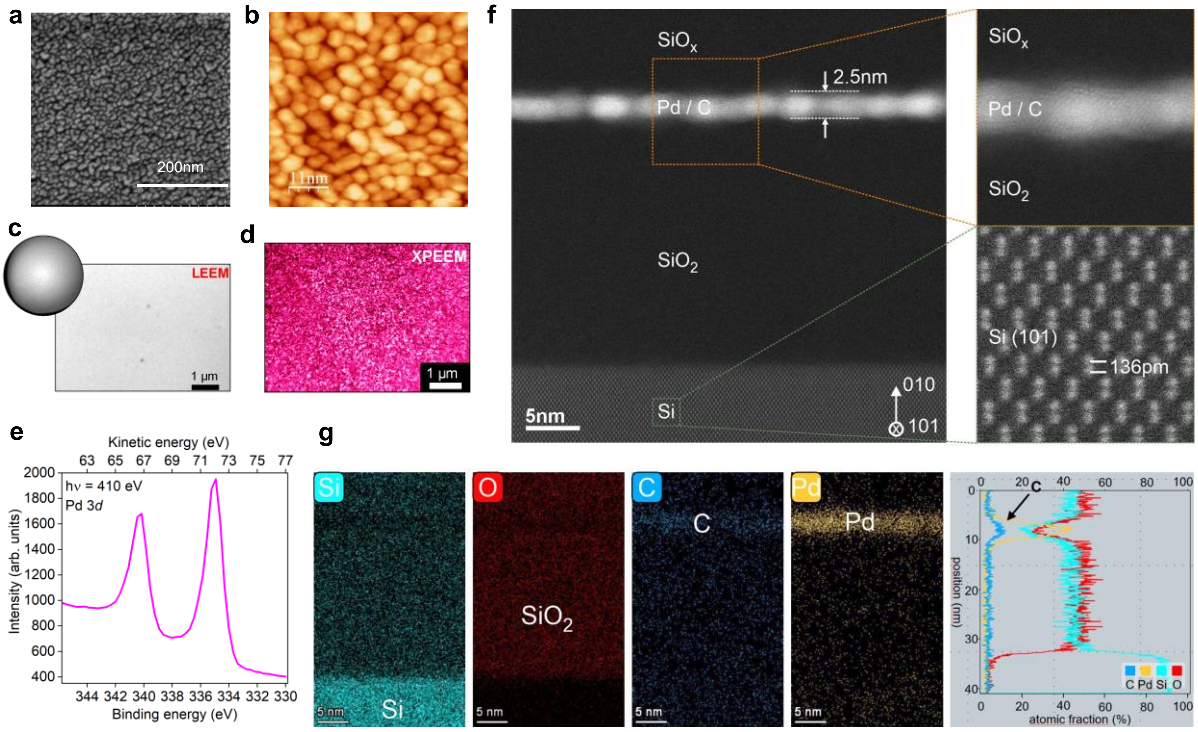

Supplementary Fig. 7 | **a** Images for as prepared Si(100) – ~20 nm SiO_2 – ~3 nm Pd by SEM. **b** Si – ~20nm SiO_2 – ~0.4nm C – ~3nm Pd by STM (V_{Bias}=3eV, I_T=nA, $P_{Chamber}$= 5.0 x 10^{-10} mbar). **c** Low energy electron microscopy image of Si(100) – ~20nm SiO_2 – ~2nm C – ~3nm Pd sample at room temperature. Inset: LEED pattern. **d** X-ray photoemission electron microscopy image normalized by the background intensity and collected at room temperature using the Pd 3*d* photoemission line. **e** Pd 3*d* photoemission spectrum collected at a photon energy of 410 eV. **f** Cross-section analysis of as prepared Pd:C thin film by STEM (Si – ~20nm SiO_2 – ~0.4nm C – ~3nm Pd). STEM-HAADF image of a Pd:C thin film on top of SiO_2 and Si (left image). The Si substrate was oriented to 101 zone axis direction. An additional electron beam-deposited SiOx layer on top of the Pd:C surface was used as protection layer for the cross section preparation by FIB. Magnified view of the Pd:C thin film (right, top) and Si substrate (bottom, right). **g** STEM-EDX maps of Si, O, C and Pd of the Pd:C thin film on top of the SiO_x and Si, and the according elemental profiles extracted from the EDX maps.

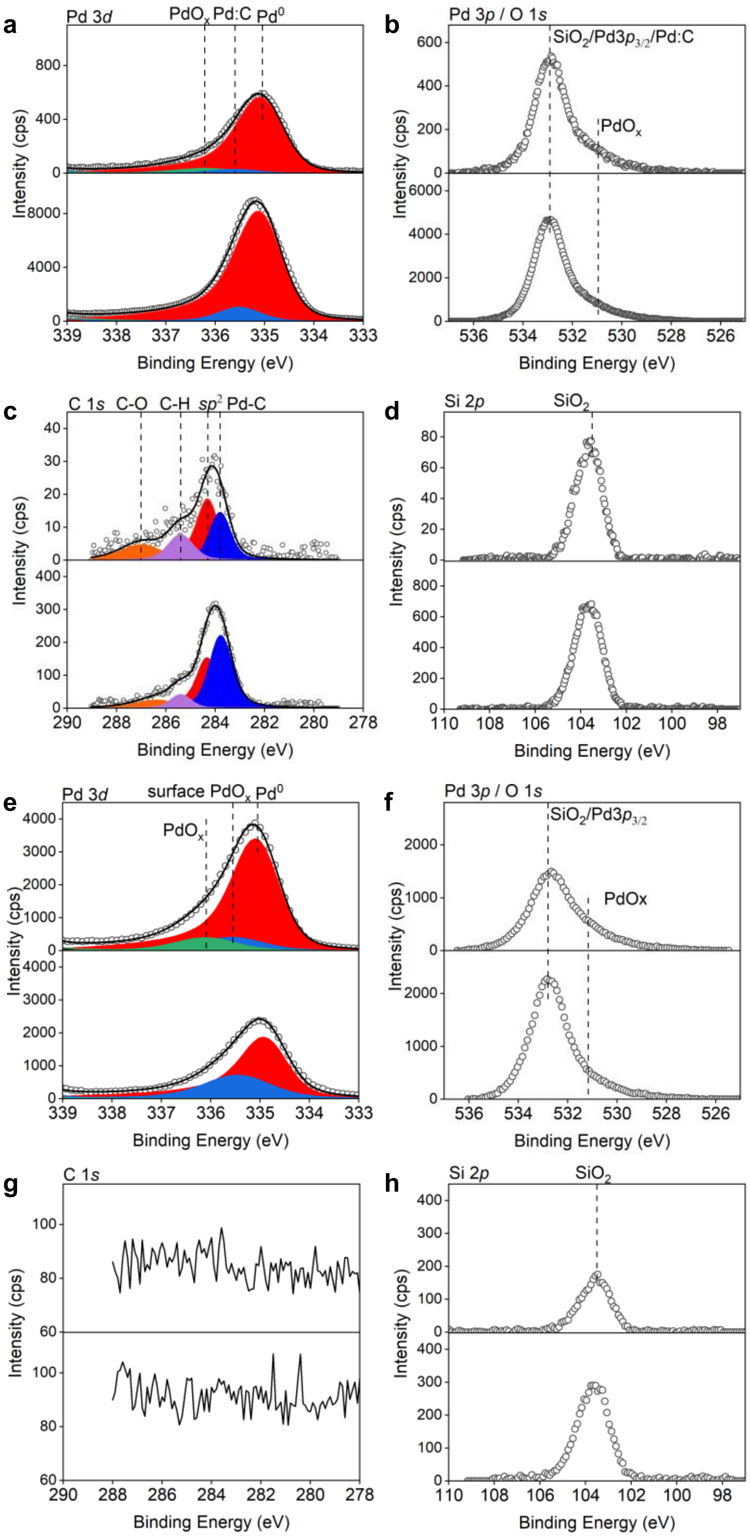

Supplementary Fig. 8 | XPS Pd 3*d* (**a**), Pd 3*p* / O 1*s* (**b**), C 1*s* (**c**) and Si 2*p* (**d**) spectra of Si − ~20nm SiO_2 − ~0.4nm C − ~3nm Pd fresh on the top and after thermal treatment on the bottom. The increased intensity in bottom panels might be caused by monochromator in X-Ray misalignment. XPS Pd 3*d* (**e**), Pd 3*p* / O 1*s* (**f**), C 1*s* (**g**) and Si 2*p* (**h**) spectra of Si − ~20nm SiO_2 − ~3nm Pd fresh on the top and after thermal treatment on the bottom. XPS employing monochromatized $Al_{k\alpha}$ radiation and a Specs electron energy analyzer (Phoibos 200).

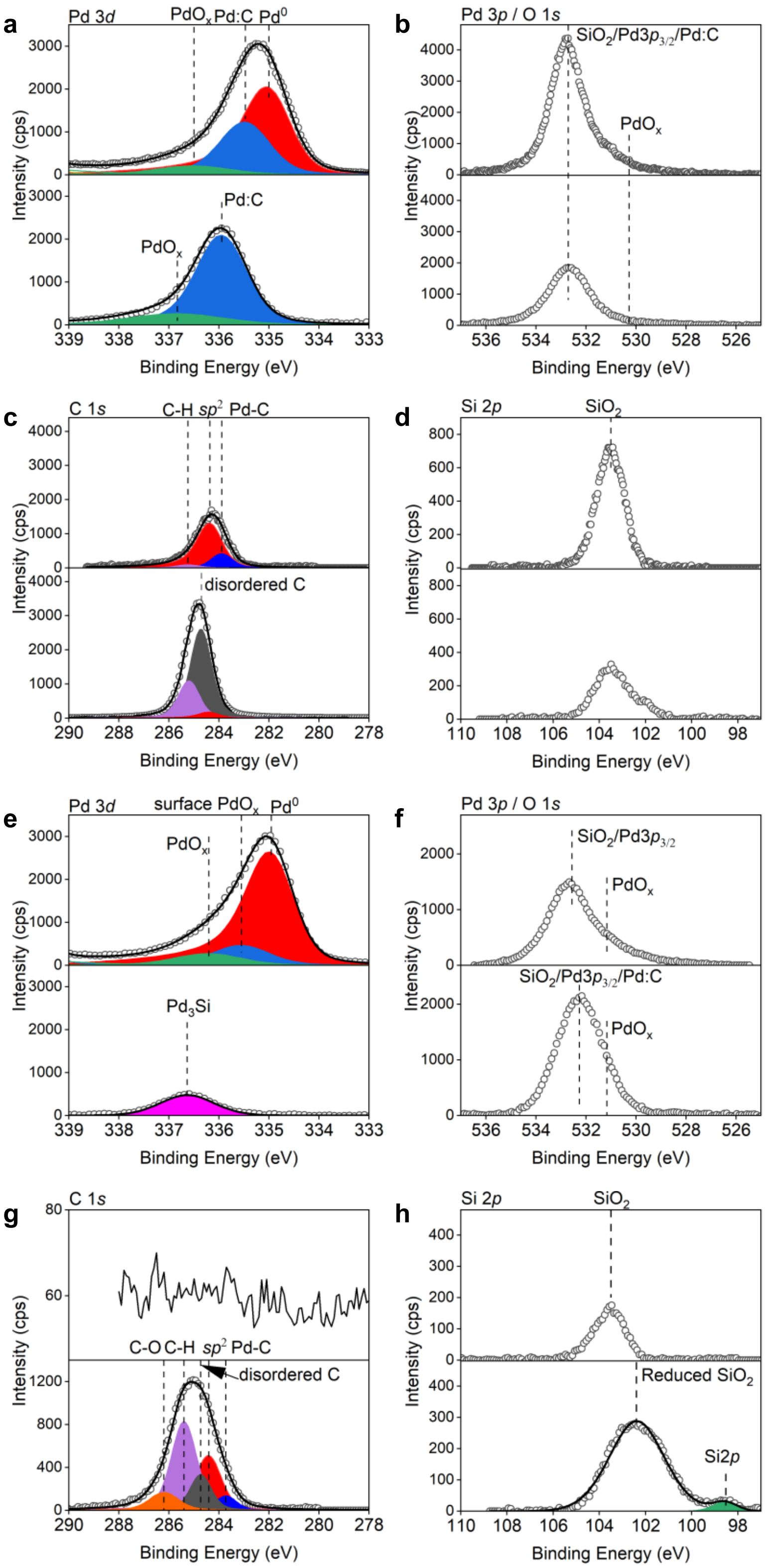

Supplementary Fig. 9 | XPS Pd 3*d* (**a**), Pd 3*p* / O 1*s* (**b**), C 1*s* (**c**) and Si 2*p* (**d**) spectra of Si – ~20nm SiO_2 – ~0.4nm C – ~3nm Pd fresh on the top and spent after catalysis on the bottom. XPS Pd 3*d* (**e**), Pd 3*p* / O 1*s* (**f**), C 1*s* (**g**) and Si 2*p* (**h**) spectra of Si – ~20nm SiO_2 – ~3nm Pd fresh on the top and spent after catalysis on the bottom. The spent samples were transported/stored in air. XPS employing monochromatized $Al_{k\alpha}$ radiation and a Specs electron energy analyzer (Phoibos 200).

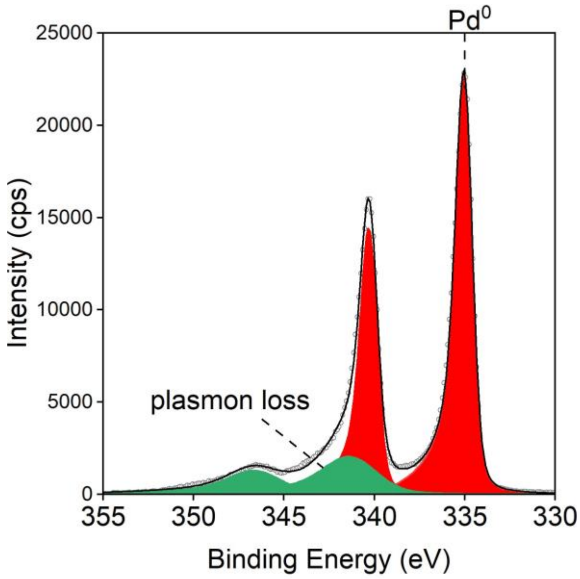

Supplementary Fig. 10 | XPS spectra of clean (3 cycles of sputtering and annealing 600°C in UHV (2.0 x 10^{-9} mbar)) Pd foil measurement with XPS employing monochromatized $Al_{k\alpha}$ radiation and a Specs electron energy analyzer (Phoibos 200).

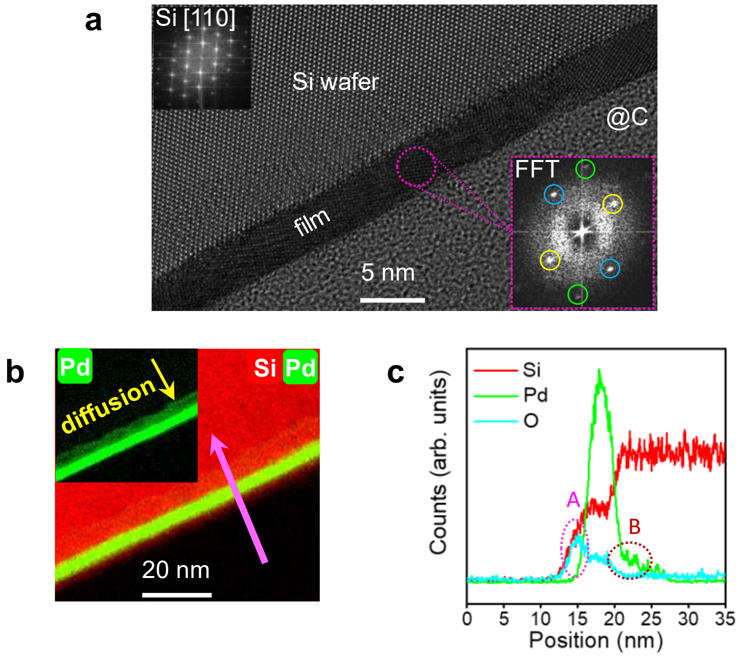

Supplementary Fig. 11 | **a** HRTEM micrograph of thin film Pd on Si [100] substrate and corresponding FFT pattern suggesting the formation of silicide (inset image). **b** EDS mapping (elements: Si, Pd) and **c** linescanning (elements: Si, Pd, O) of (**b**) showing the Pd diffusion in the substrate (EDS-B) and the formation of SiO_x on the top of film (EDS-A).

a

b

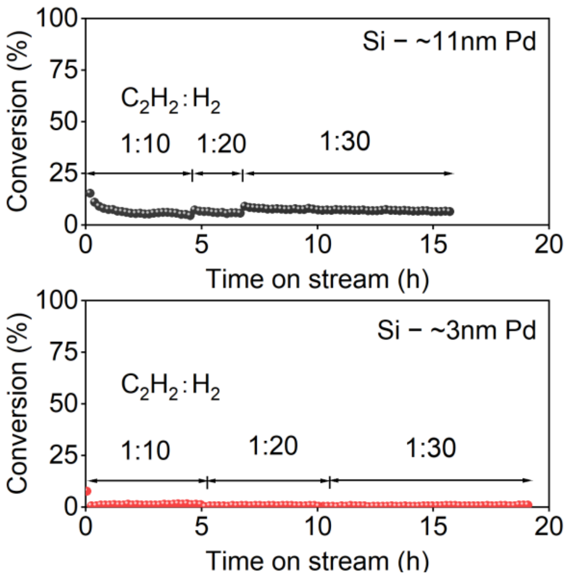

Supplementary Fig. 12 | Catalytic conversion of (**a**) Si − ~11nm Pd and (**b**) Si − ~3nm Pd. Reaction condition: catalyst 3 mm x 20 mm; C_2H_2: 0.9 ml/min; H_2 from 9, 18, to 27 ml/min; N_2 balance; T = 125°C.

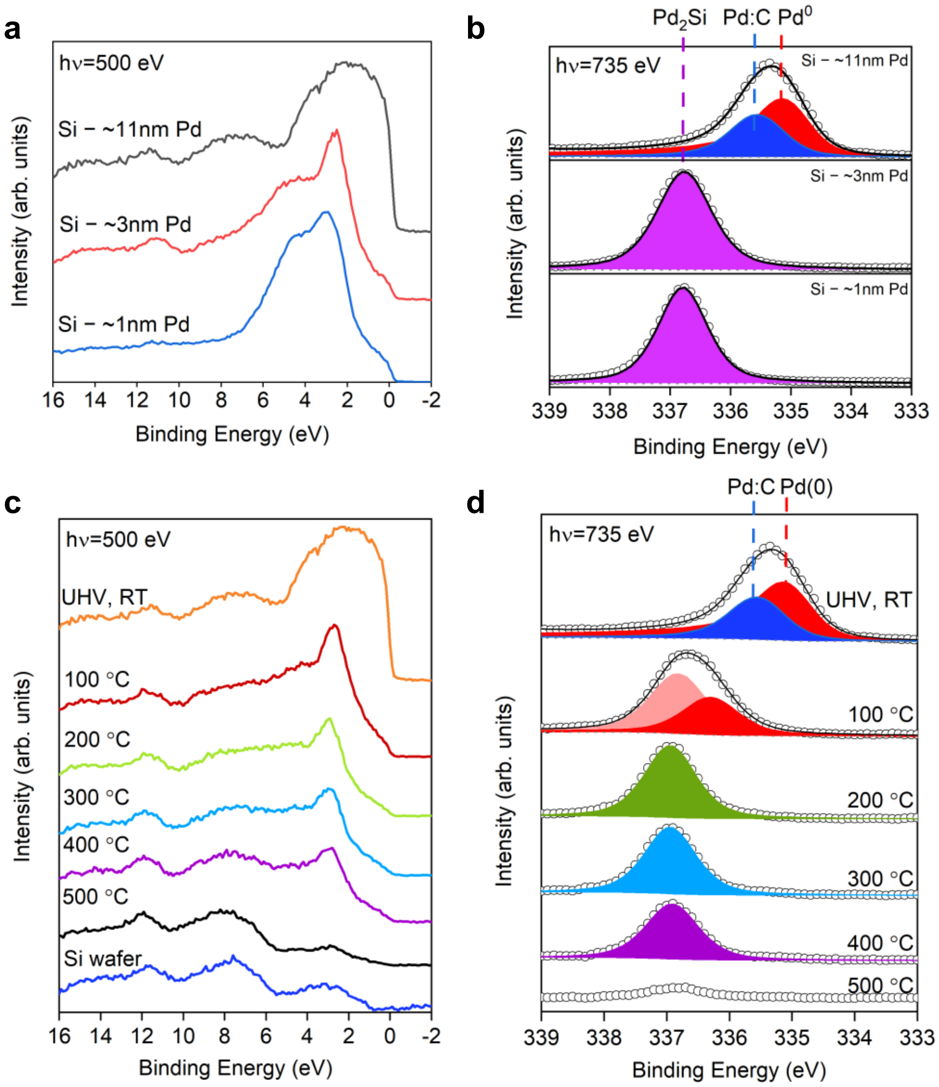

Supplementary Fig. 13 | **a** Valence band and **b** Pd 3$d_{5/2}$ spectra of Si – ~1nm Pd, Si – ~3nm Pd, and Si – ~11nm Pd. **c** Valence band and **d** Pd 3$d_{5/2}$ spectra of Si – ~11nm Pd in 0.1 mbar H_2. XPS was measured at the synchrotron BESSY II.

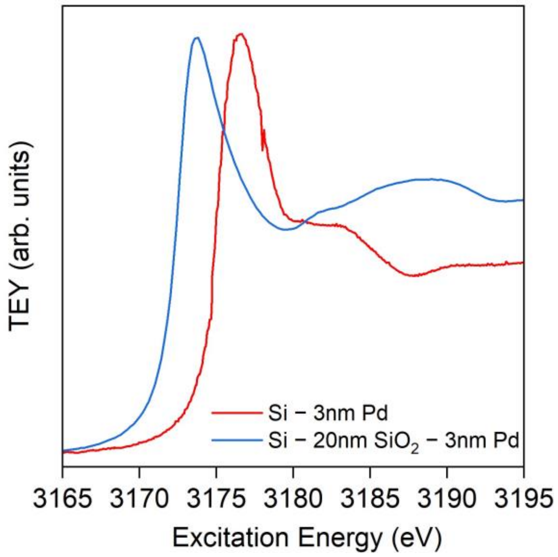

Supplementary Fig. 14a | Pd L_3-edge spectra of Si – 3 nm Pd and Si – 20 nm SiO_2 – 3 nm Pd LCC measured at room temperature in UHV. The NEXAFS spectrum of Si – 20 nm SiO_2 – 3 nm Pd exhibits a white line at 3174.2 eV typical of metallic Pd[11]. The white line of Si – 3 nm Pd is shifted around 3 eV towards higher energies, which matches with the Pd silicide structure[37]. The observations of Pd silicide and metallic Pd are in agreement with the XPS and TEM results presented above. NEXAFS was measured at the synchrotron BESSY II.

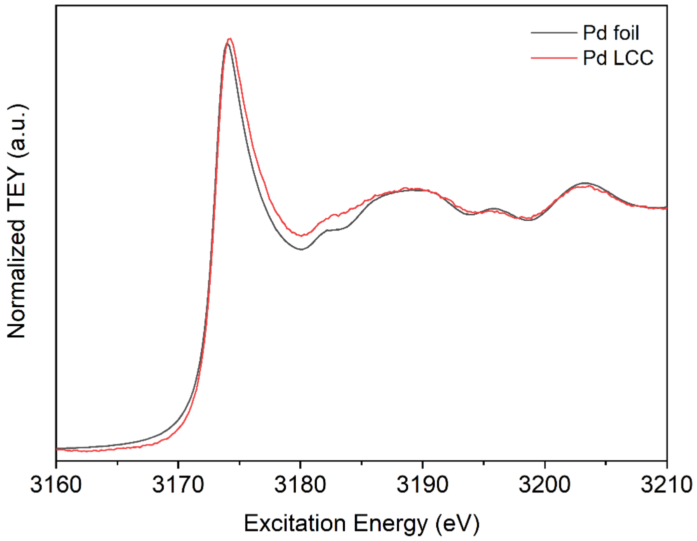

Supplementary Fig. 14b | Pd L_3-edge spectra of Si – 20 nm SiO_2 – 3 nm Pd (Pd LCC) and Pd foil measured at room temperature and in UHV. NEXAFS was measured at the synchrotron BESSY II.

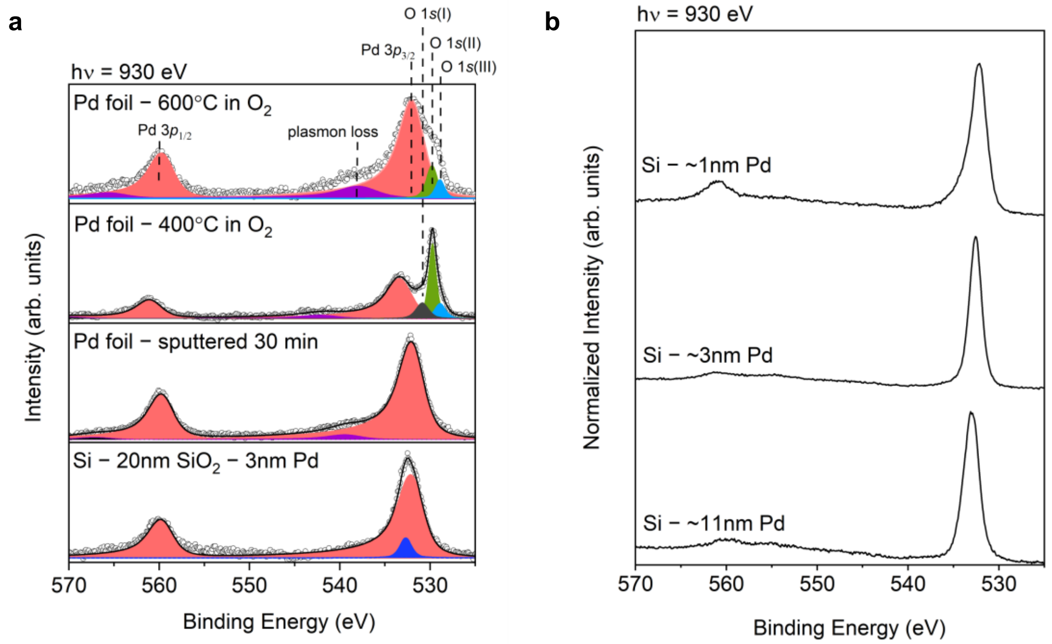

Supplementary Fig. 15 | **a** XPS Pd 3*p*/O 1*s* spectra of Si – 20 nm SiO_2 – 3 nm Pd, and sputter-cleaned Pd foil in UHV and RT. The spectra Pd foil at 400°C and 600°C are acquired in 0.1 mbar O_2. O 1*s*(I), O 1*s*(II), and O 1*s*(III) respectively belong to adsorbed oxygen layer, surface plus bulk Pd oxide, and surface Pd oxide. **b** XPS Pd 3*p*/O 1*s* spectra of Si – ~1 nm Pd, Si – ~3 nm Pd, and Si – ~11 nm Pd samples in UHV and RT. XPS was measured at the synchrotron BESSY II.

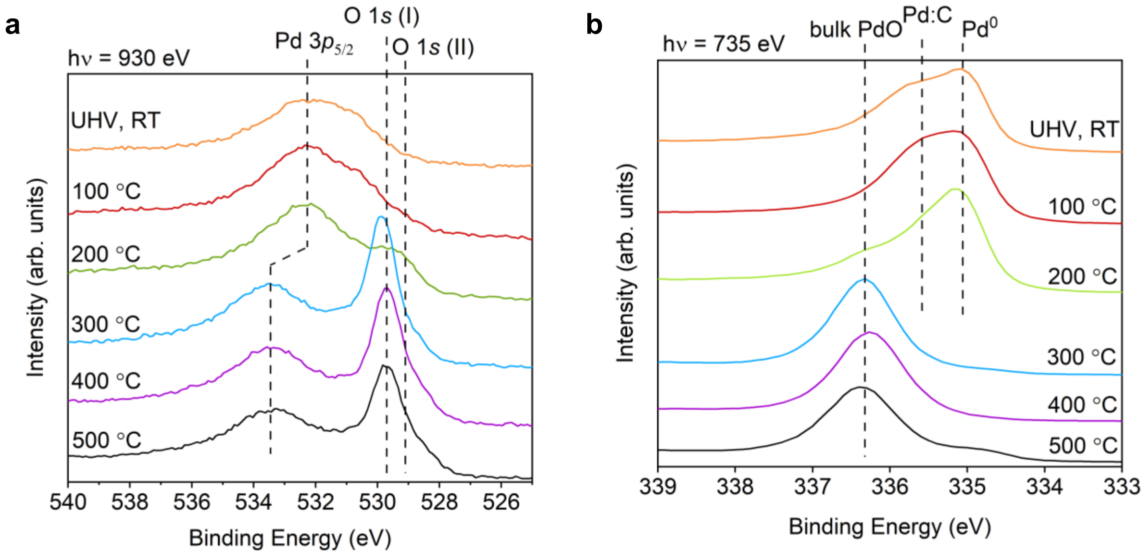

Supplementary Fig. 16 | (**a**) XPS Pd 3*p*/O 1*s* and (**b**) Pd $3d_{5/2}$ spectra of Si − 200 nm SiO_2 − 10 nm Pd measured in UHV and RT, and during the in-situ heating experiment in 0.1 mbar O_2. The vertical dashed lines are a guide to the eye for easier following of the peaks. O 1*s* (I) belongs to the contributions from bulk and surface Pd oxide, whereas O 1*s* (II) belongs to the contribution from only surface Pd oxide[13,14]. XPS was measured at the synchrotron BESSY II.

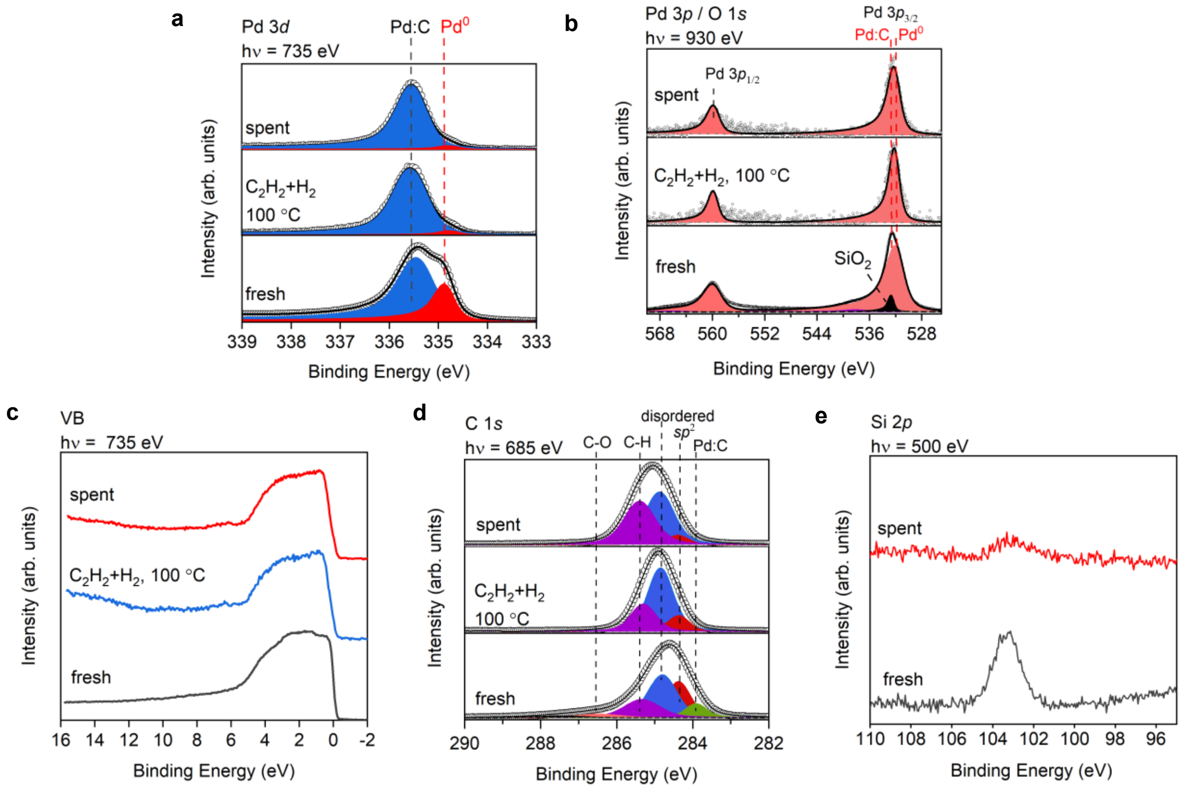

Supplementary Fig. 17 | XPS Pd 3*d* (**a**), Pd 3*p* / O 1*s* (**b**), valence band (**c**), C 1*s* (**d**) and Si 2*p* (**e**) spectra of Si − 20 nm SiO_2 – 3 nm Pd before (fresh) under the operando acetylene hydrogenation at 1 mbar and after (spent). Kinetic energy of photoelectrons is 400 eV. Pd 3$p_{3/2}$ peaks are observed at 532.2 eV indicating metallic Pd, and the black peak observed in the fresh sample at 532.7 eV belongs to O 1*s* of SiO_2. The conversion and selectivity values at 100°C in 1 mbar C_2H_2+H_2 are respectively 0.24 % and 95.0 %. XPS was measured at the synchrotron BESSY II.

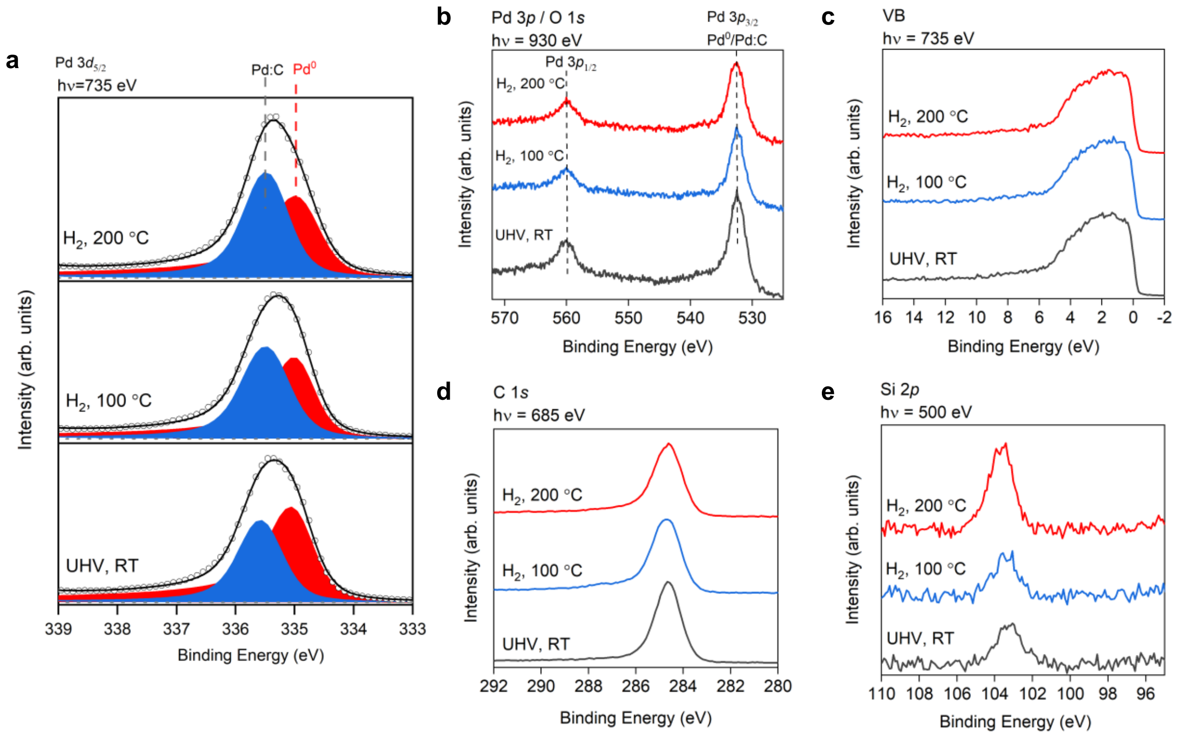

Supplementary Fig. 18 | XPS Pd 3*d* (**a**), Pd 3*p* / O 1*s* (**b**), valence band (**c**), C 1*s* (**d**) and Si 2*p* (**e**) of Si – 20 nm SiO_2 – 3 nm Pd during heating in 0.1 mbar H_2. The metallic Pd peak is observed at 335 eV and the Pd:C peak is observed at 335.5-335.6 eV. XPS was measured at the synchrotron BESSY II.

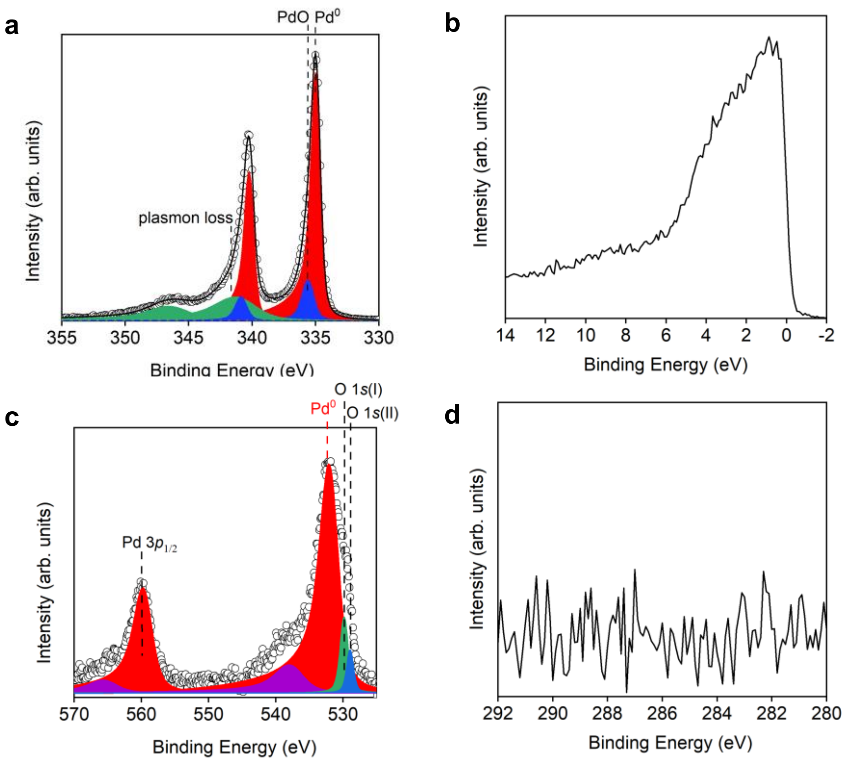

Supplementary Fig. 19 | XPS Pd 3*d* (**a**), valence band (**b**), Pd 3*p* / O 1*s* (**c**) and C 1*s* (**d**) spectrum of clean Pd foil in 3E-2 mbar O_2 at 600°C. Kinetic energy of photoelectrons is 400 eV. XPS was measured at the synchrotron BESSY II. The Pd 3*d* spectrum is deconvoluted into two peaks at 335.0 eV for metallic Pd and 355.6 eV for surface Pd oxide, in agreement with the literature[7]. The surface oxide peaks are also visible in Pd 3*p*/O 1*s* spectrum as O 1*s*(I) and O 1*s*(II). The doublets at higher binding energies (342-347 eV) belongs to the plasmon loss peaks of metallic Pd. The Pd 3$p_{3/2}$ / O 1*s* spectra includes peaks at 529.8 - O 1*s*(I) and 528.9 eV - O 1*s*(II) indicating surface Pd oxide contributions. Pd 3$p_{3/2}$ peak is positioned at 532.1 eV, accompanied with plasmon loss peak at 538.1 eV. Since the measurement is performed in-situ in O_2, there is no carbon signal in C 1*s*.

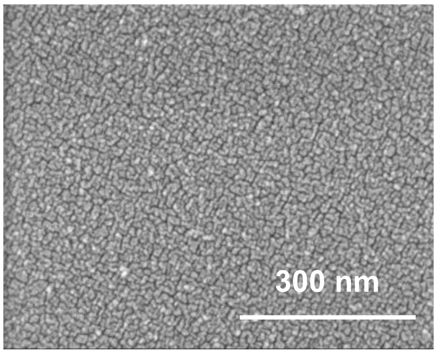

Supplementary Fig. 20a | Surface morphology of as-prepared sputtered LCC Si – 20 nm SiO_2 – 3 nm Pd measured by SEM.

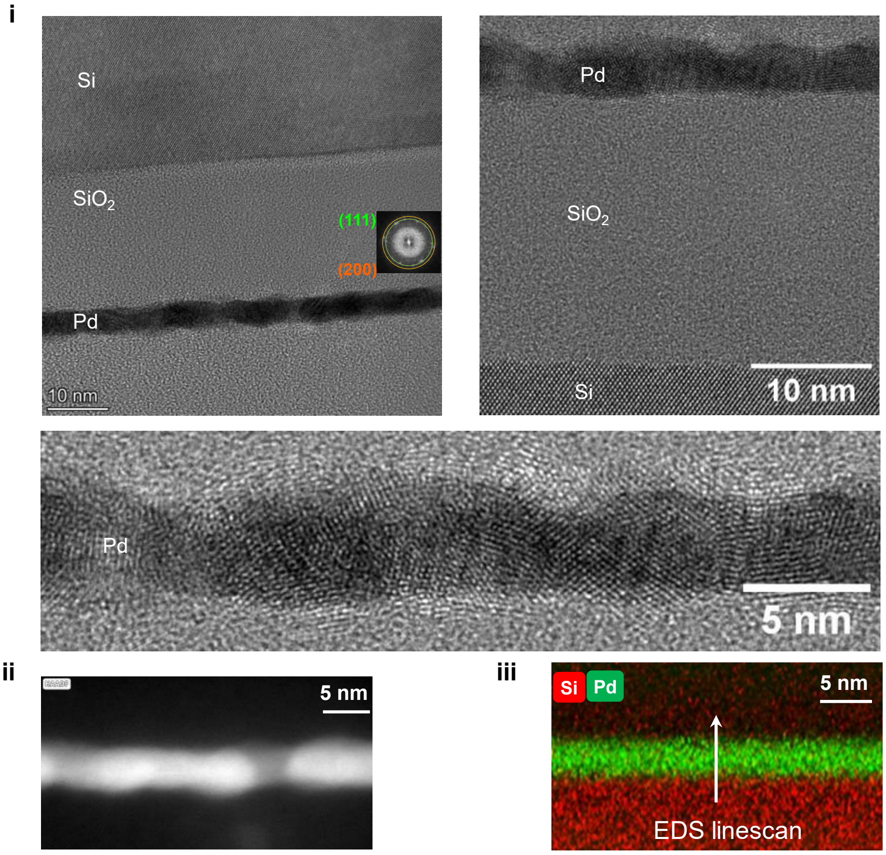

Supplementary Fig. 20b | **Microscopy on as-prepared LCC Pd. i** Cross-section HRTEM micrograph of Si – 20 nm SiO_2 – 3 nm Pd and corresponding FFT pattern showing the polycrystallinity and the cubic Pd phase of the film (inset image). Images at higher magnification are also shown. **ii** HAADF image of the LCC. **iii** Cross-section EDS elemental mapping by linescanning (elements: Si, Pd, O).

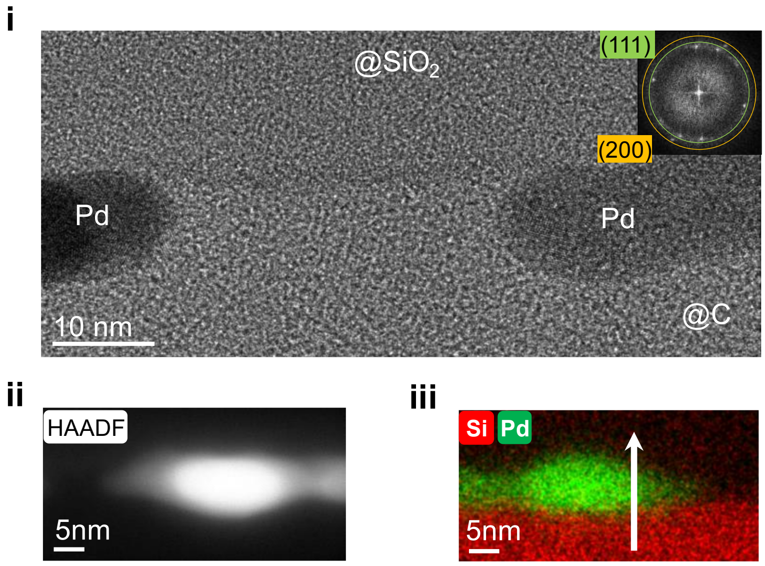

Supplementary Fig. 20c | Microscopy on spent LCC Pd. i HRTEM micrograph of a spent Si – 20 nm SiO_2 – 3 nm Pd depicting the partial removal of nanostructures after catalytic reaction. The corresponding FFT pattern reports metallic Pd of polycrystalline cubic structure. The presence of C cannot be detected by the limited precision of the measurement of the d-spacing. **ii** HAADF image of the LCC. **iii** EDS elemental mapping (elements: Si, Pd).

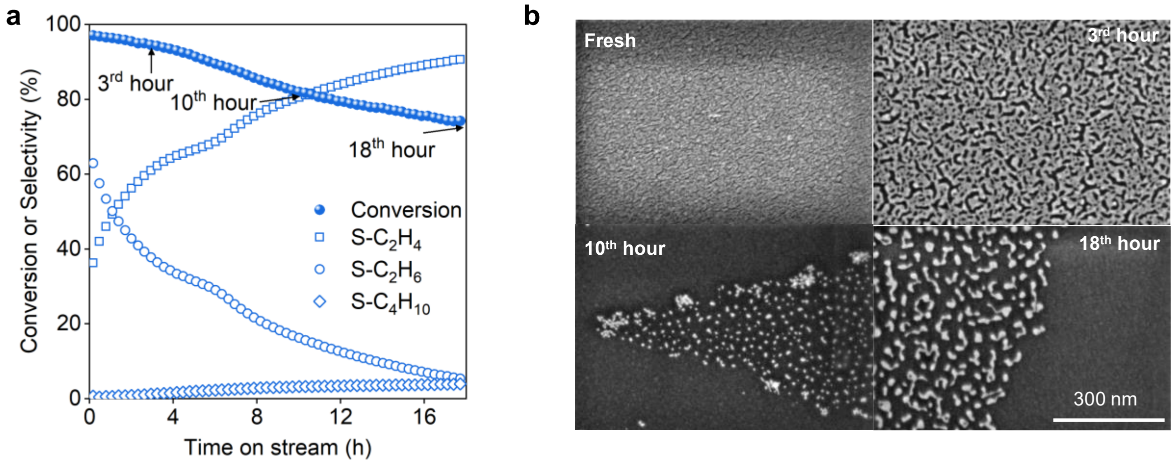

Supplementary Fig. 21 | **a** Time on stream stability of LCC Pd: Si – 20 nm SiO_2 – 3 nm Pd under reaction condition. **b** SEM surface morphology of fresh and spent LCC at 3rd, 10th and 18th hour (see arrows in **a**). Reaction condition: C_2H_2: 0.9 ml/min; H_2: 27 ml/min; N_2: 5.8 ml/min; T = 150°C.

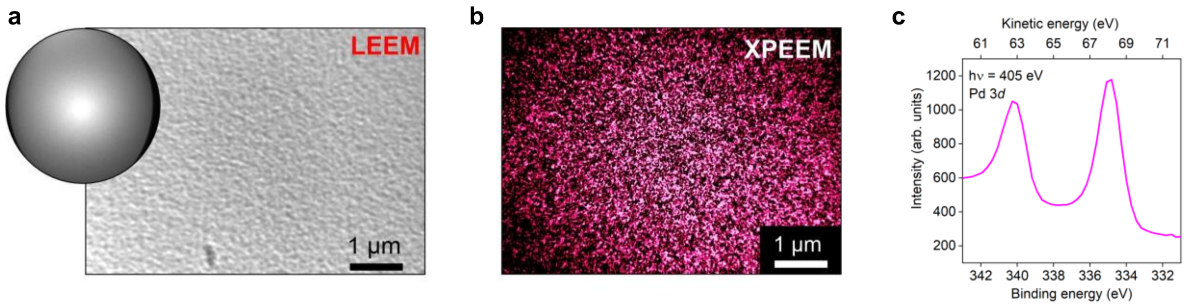

Supplementary Fig. 22 | **a** Low energy electron microscopy image of Si − 20 nm SiO_2 − 3 nm Pd sample collected at an electron energy of 2.4 eV and at room temperature. Inset: LEED pattern collected at an electron energy of 42 eV. **b** X-ray photoemission electron microscopy image normalized by the background intensity and collected at room temperature using the Pd 3*d* photoemission line. **c** Pd 3*d* photoemission spectrum collected at a photon energy of 405 eV.

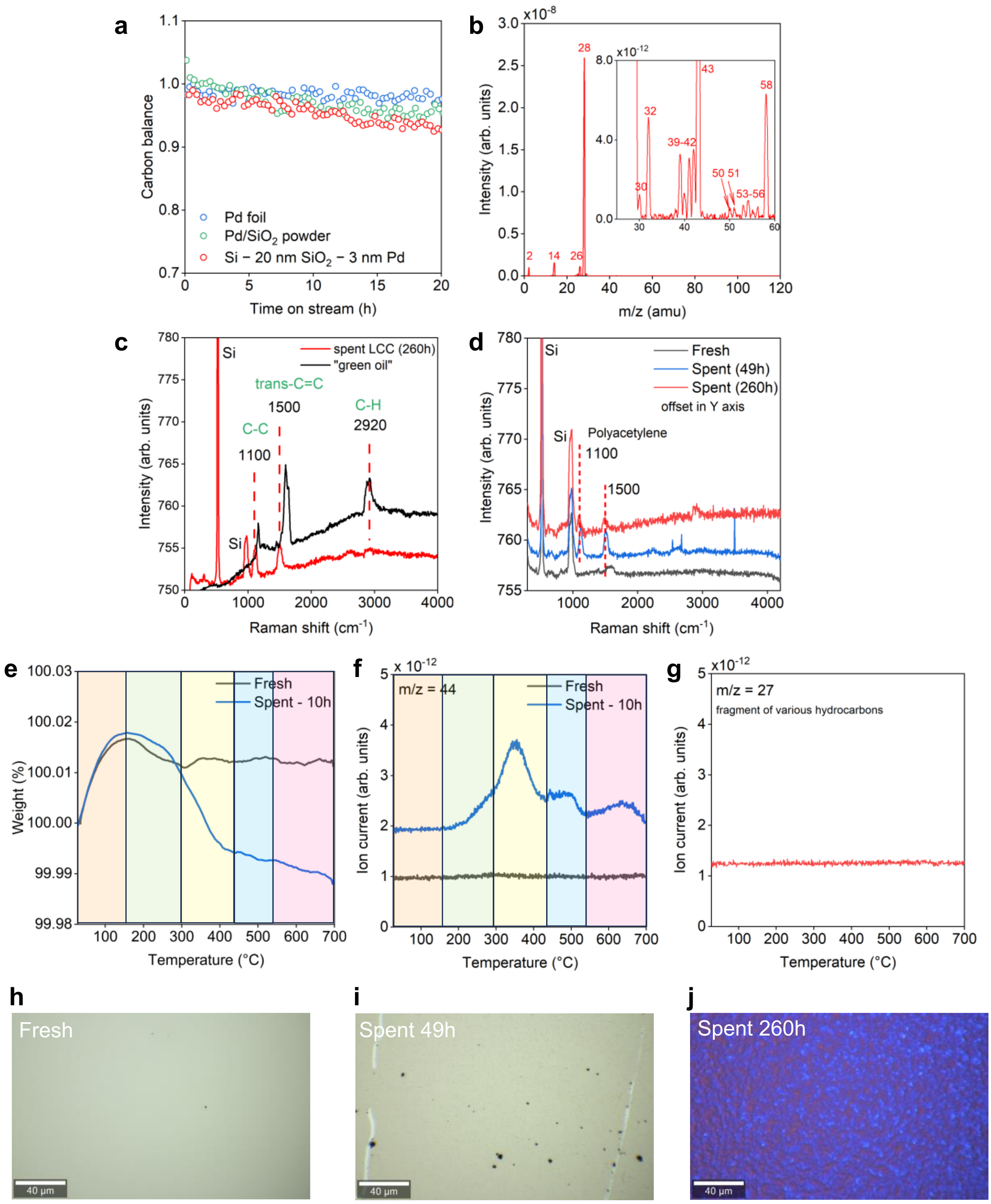

Supplementary Fig. 23 | Observation of carbon deposits. **a** Carbon balance of the catalytic acetylene hydrogenation on LCC Pd, Pd powder and Pd foil. **b** Mass spectra (analog scan) on the product gas on LCC Pd at TOS = 66h. **c** In-situ Raman spectra of "green oil" produced in the Raman cell (black, TOS=27h) and carbon deposits on spent LCC Pd after TOS=260h (red). **d** Carbon deposits observed by Raman spectroscopy for fresh, spent Pd LCCs (TOS= 49h, 260h). **e** TG and **f** MS observation on fresh and spent LCC Pd in synthetic air (21% O_2 in Ar) and **g** inert Ar. Temperature was increased from RT to 700 °C at rate of 10°C/min in TG-MS. **h-j** Light microscopy images obtained by Raman for the fresh, spent Pd LCCs (TOS= 49h, 260h). Reaction condition: (a) C_2H_2: 0.9 ml/min, H_2: 27 ml/min, N_2: 5.8 ml/min, T = 150°C and the spent LCC in (c-g, i, j) for Raman; (b) C_2H_2: 0.9 ml/min, H_2: 0.9 ml/min, N_2: 31.9 ml/min, T = 150°C; and (c) C_2H_2: 1.5 ml/min, H_2: 1.5 ml/min, N_2: 27 ml/min, T = 150°C for in-situ Raman.

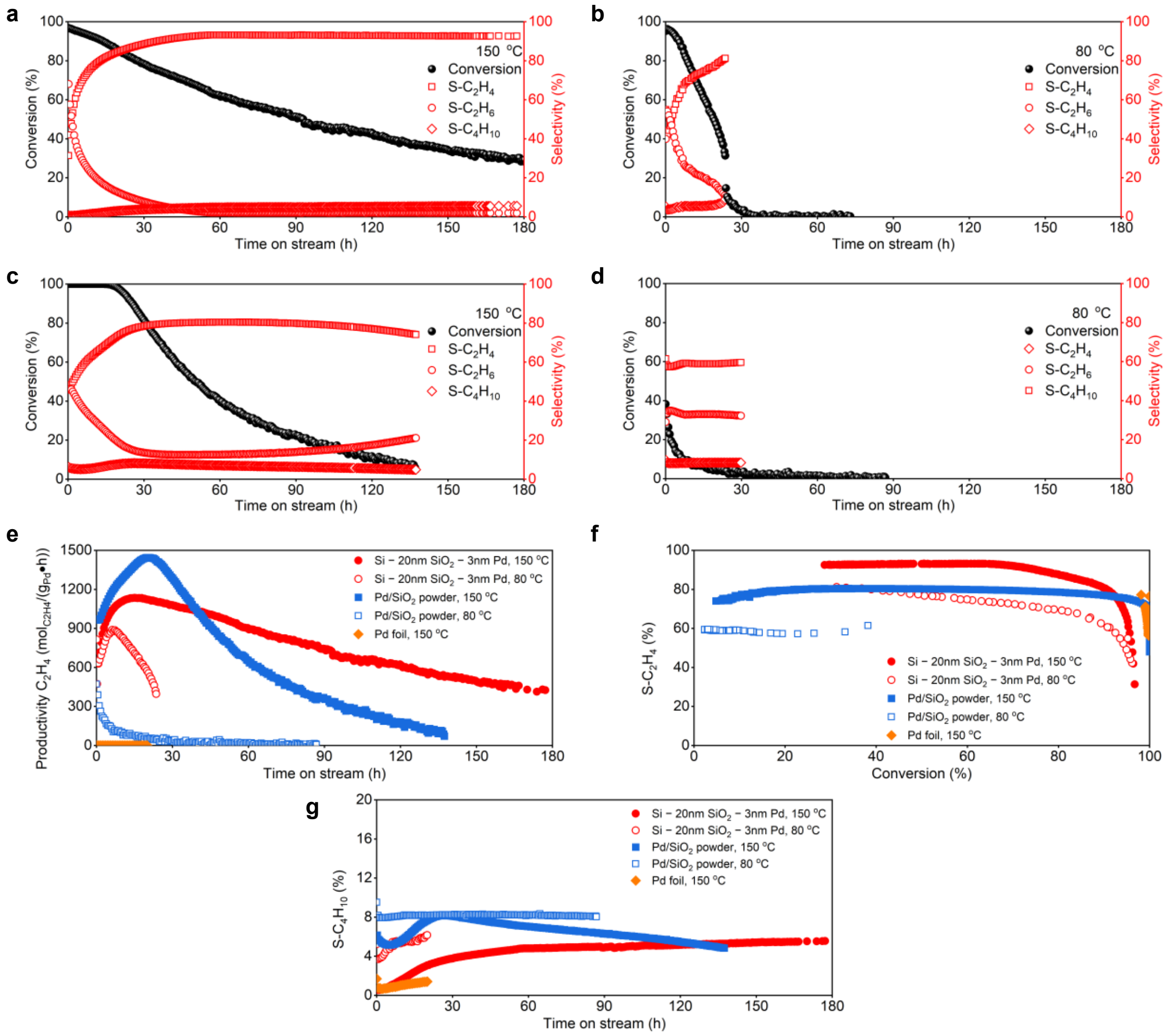

Supplementary Fig. 24 | Catalytic long-time stability of LCC, powder, foil Pd. Catalytic activity and selectivity: **a** Si – 20 nm SiO_2 – 3 nm Pd, 150°C, 180 hours; **b** Si – 20 nm SiO_2 – 3 nm Pd, 80°C, 73 hours; **c** Pd/SiO_2 powder, 150°C, 138 hours; **d** Pd/SiO_2 powder, 80°C, 87 hours. The comparison of the productivity (**e**), selectivity (**f,g**) between LCC, powder and foil Pd. Reaction condition: C_2H_2: 0.9 ml/min; H_2: 27 ml/min; N_2: 5.8 ml/min. Samples in the experiment: LCC (3 mm x 20 mm, 1.63 μg Pd) and Pd foil (3 mm x 20 mm, ~0.2 mm thick), Pd/SiO_2 powder (1.26 μg Pd loading in 0.5 g SiO_2).

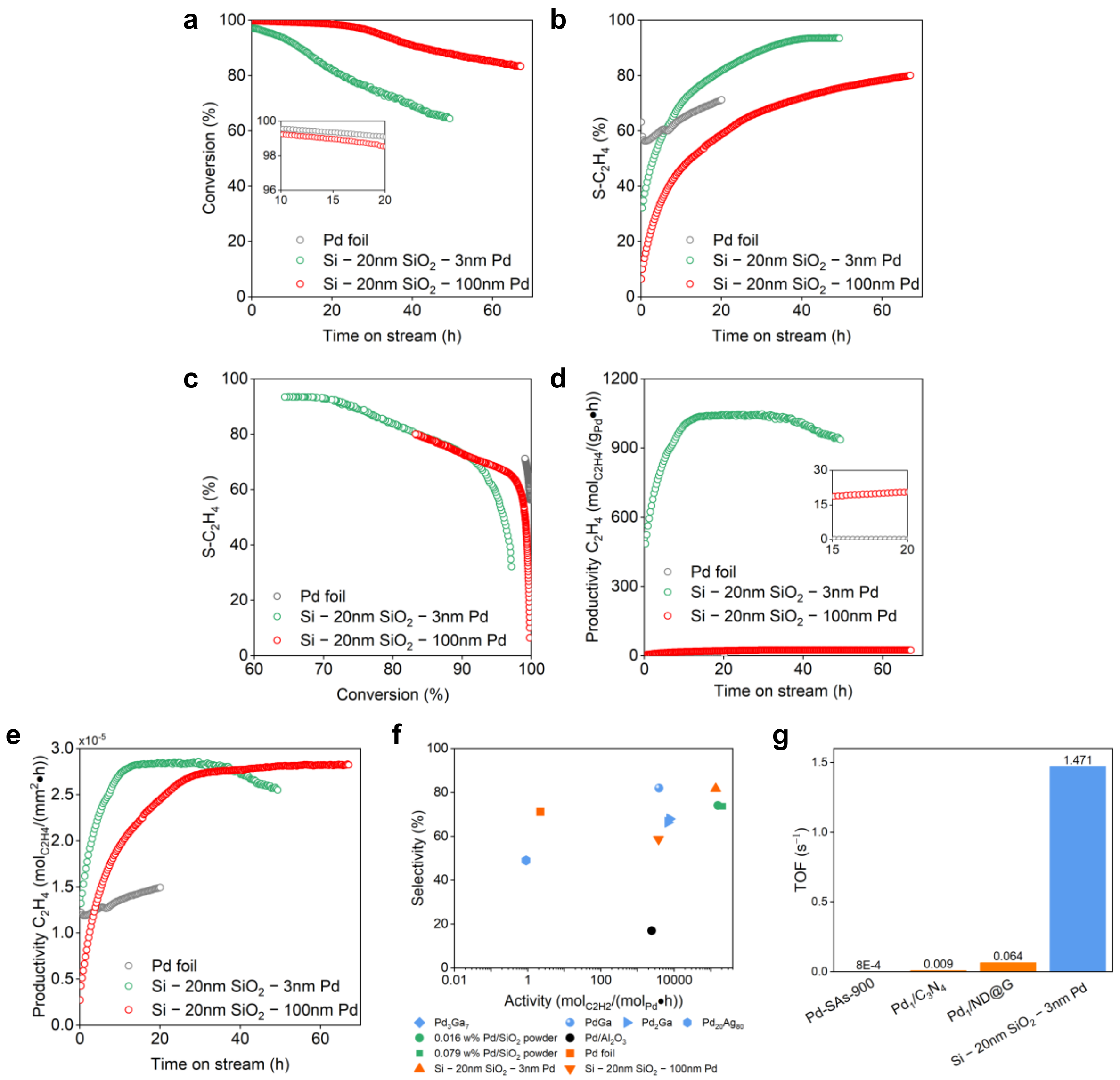

Supplementary Fig. 25 | Comparisons between 3 nm, 20 nm, 100nm, thin films, Pd foil and literature data. Catalytic conversion (**a**) and selectivity (S-C_2H_4) (**b,c**) of Si – 20 nm SiO_2 – 3 nm Pd, Si – 20 nm SiO_2 – 100 nm Pd, and Pd foil. Productivity of C_2H_4 with time on stream during acetylene hydrogenation reactions, normalized either by mass Pd (**d**) or surface area of Pd layer (**e**). **f** Catalytic selectivity-activity plot on LCC Pd and the reported monometallic Pd and bimetallic PdGa and PdAg catalysts[23], data at 20h time on stream from acetylene hydrogenation. **g** Turn over frequency of Si – 20 nm SiO_2 – 3 nm Pd and the comparison to reported single-atom Pd catalysts[31-33]. Reaction condition: C_2H_2: 0.9 ml/min; H_2: 27 ml/min; N_2: 5.8 ml/min at T = 150°C (**a-f**) and T = 80°C (**g**).

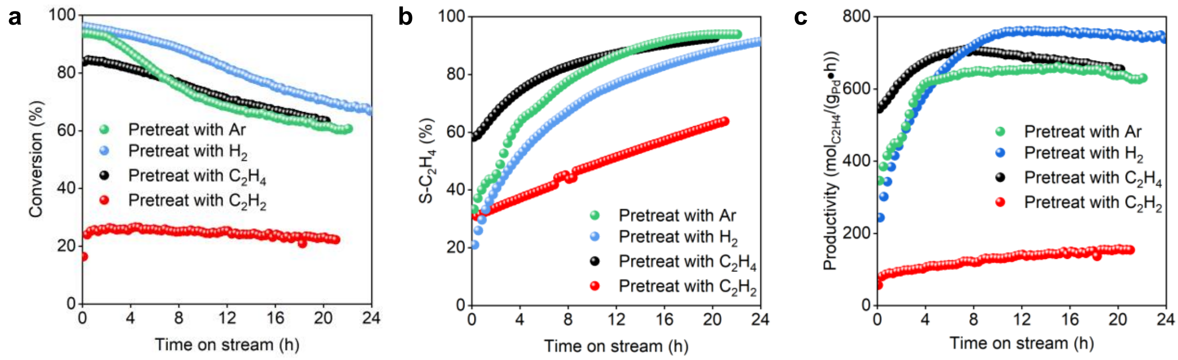

Supplementary Fig. 26 | Catalytic conversion (**a**), selectivity (**b**) and productivity (**c**) of Si − 200 nm SiO_2 − 3 nm Pd. LCC was pretreated with either Ar, 30% H_2, 30% C_2H_4, or 30% C_2H_2 before catalysis (total flow 30 ml/min). Reaction condition: C_2H_2: 0.9 ml/min; H_2: 27 ml/min; N_2: 5.8 ml/min; T = 150°C.

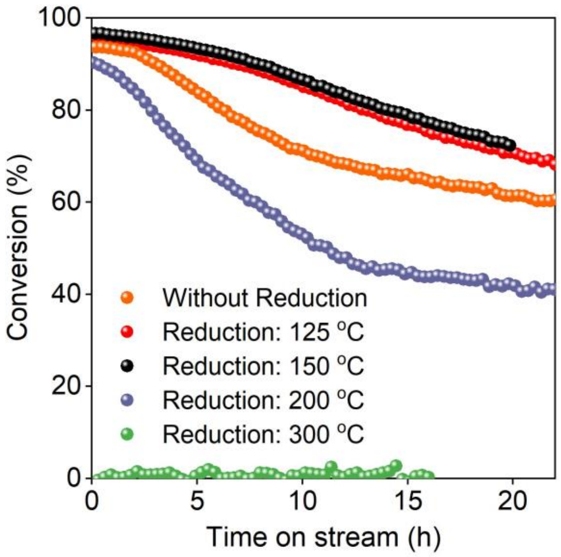

Supplementary Fig. 27 | Catalytic conversion of Si − 200 nm SiO_2 − 3 nm Pd after reduced at different temperatures. LCC was pretreated 30% H_2 at 125, 150, 200, 300°C or without reduction (100% Ar) before catalysis (total flow 30 ml/min, 30 mins). Reaction condition: C_2H_2: 0.9 ml/min; H_2: 27 ml/min; N_2: 5.8 ml/min; T = 150°C.

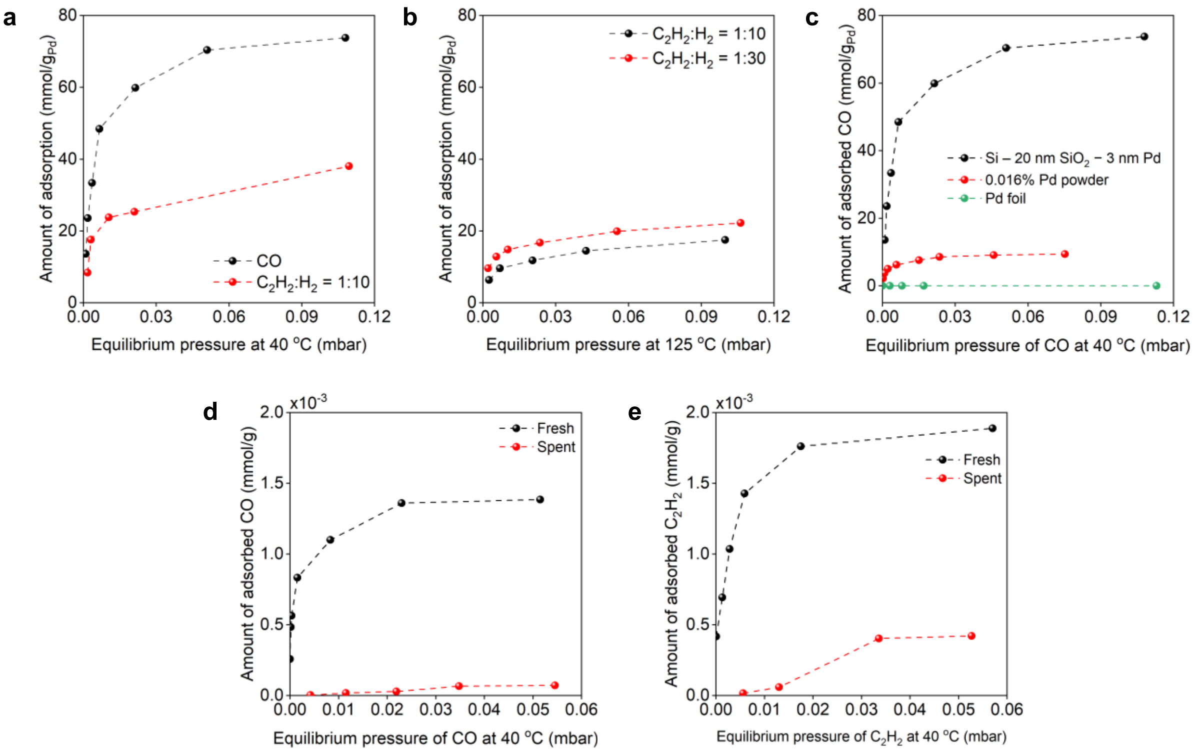

Supplementary Fig. 28 | **a** CO and real reaction gas mixture C_2H_2:H_2 volumetric adsorption on Si − 20 nm SiO_2 − 3 nm Pd. **b** Reaction gas mixture C_2H_2:H_2 with ratios of 1:10 and 1:30 volumetric adsorption on Si − 20 nm SiO_2 − 3 nm Pd. **c** CO volumetric adsorption on Si − 20 nm SiO_2 − 3 nm Pd, Pd foil and powder catalysts. **d** CO volumetric adsorption and **e** C_2H_2 volumetric adsorption on both fresh and spent Si − 20 nm SiO_2 − 3 nm Pd.

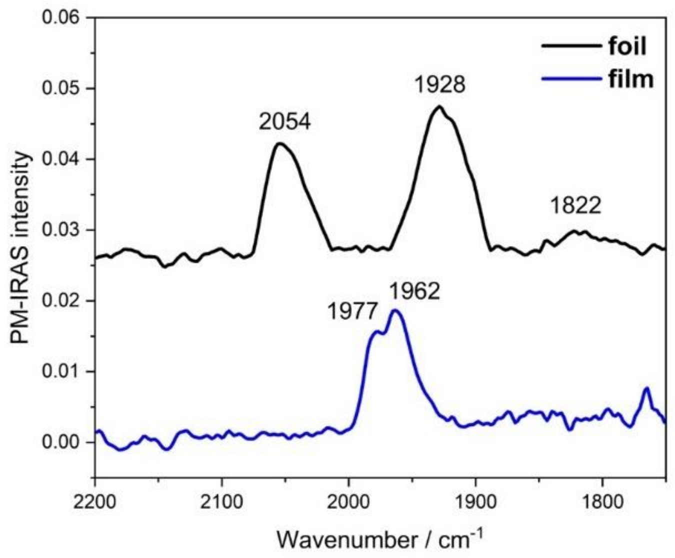

Supplementary Fig. 29 | PM-IRAS spectroscopy of CO adsorption on Pd foil and Si − 20 nm SiO_2 − 3 nm Pd at 40°C (vacuum).

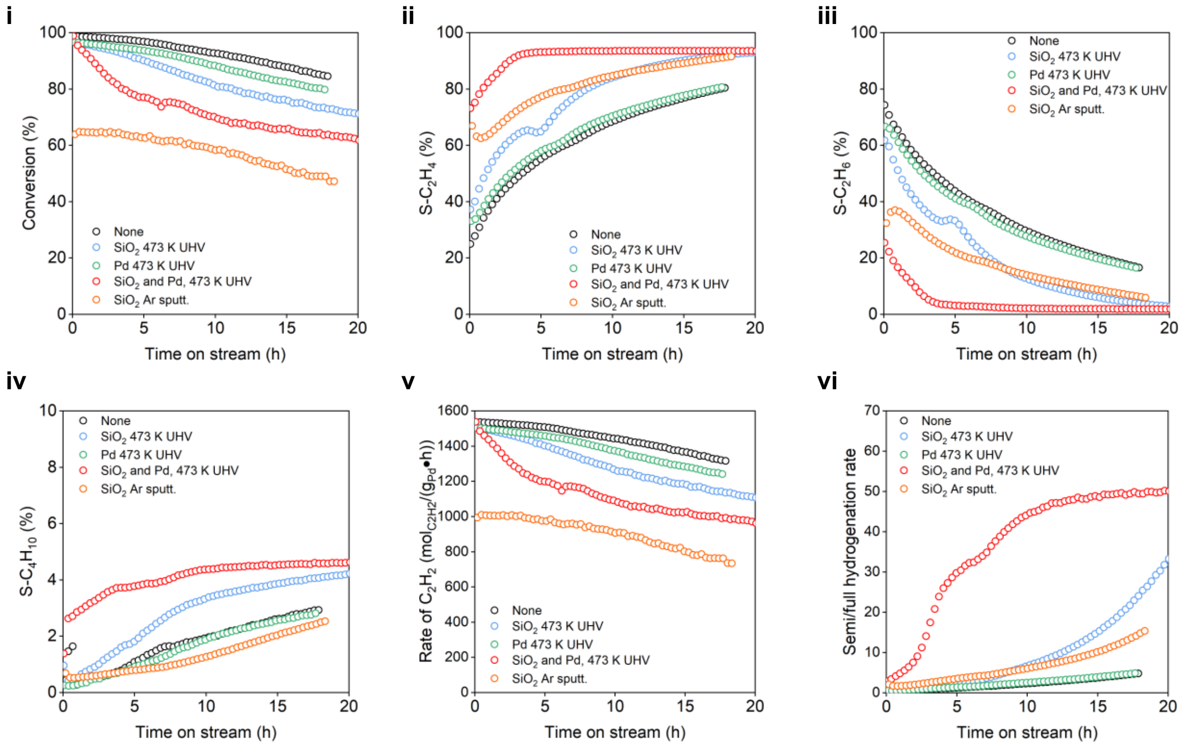

Supplementary Fig. 30a | Comparison on the LCCs Pd (Si − 20 nm SiO_2 − 3 nm Pd) with annealing pretreatments on different layers. (**i**) Conversion, (**ii**) selectivity S-C_2H_4, (**iii**) selectivity S-C_2H_6, (**iv**) selectivity S-C_4H_{10}, (**v**) C_2H_2 consumption rate, (**vi**) semi/full hydrogenation rate. Si − 20 nm SiO_2 − 3 nm Pd were pretreated (None; SiO_2 473 K, UHV; Pd 473 K, UHV; SiO_2 and Pd 473 K, UHV; SiO_2 Ar sputtering.) Reaction condition: C_2H_2: 0.9 ml/min; H_2: 27 ml/min; N_2: 5.8 ml/min; T = 150°C.

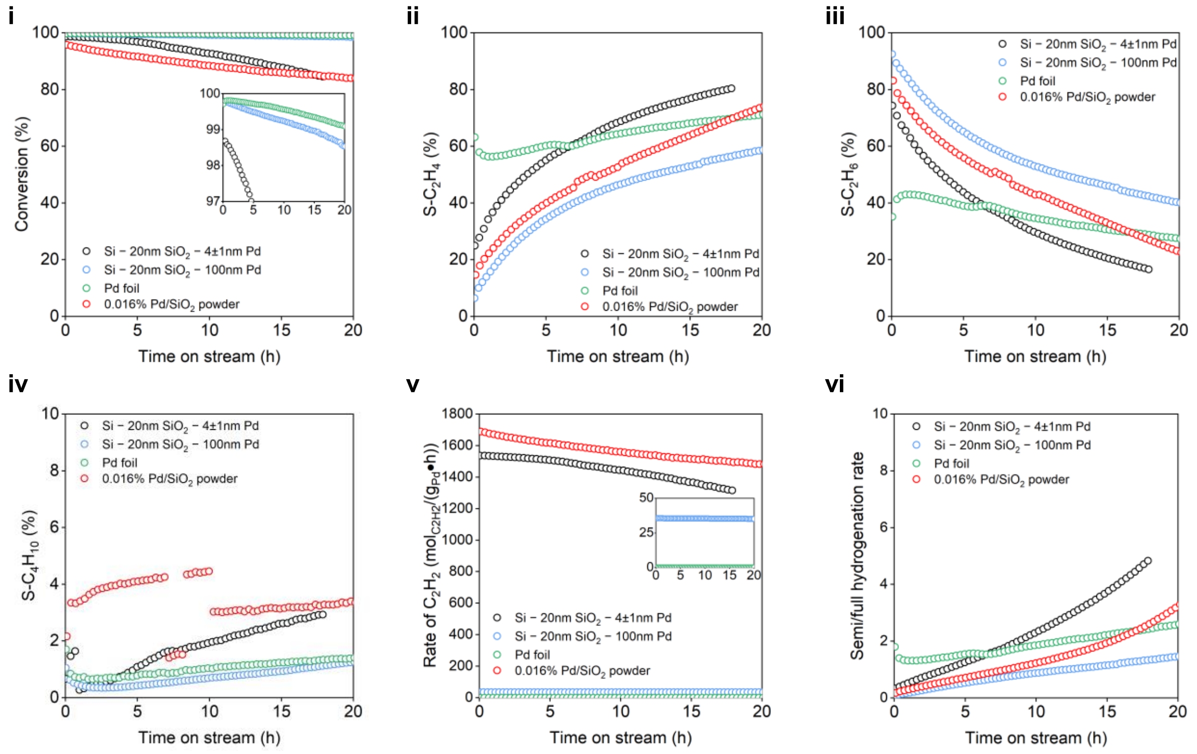

Supplementary Fig. 30b | Comparison of 3 nm Pd, 100nm Pd, Pd foil and Pd/SiO2 powder. (**i**) Conversion, (**ii**) selectivity S-C_2H_4, (**iii**) selectivity S-C_2H_6, (**iv**) selectivity S-C_4H_{10}, (**v**) C_2H_2 consumption rate, (**vi**) semi/full hydrogenation rate. Reaction condition: C_2H_2: 0.9 ml/min; H_2: 27 ml/min; N_2: 5.8 ml/min; T = 150°C.

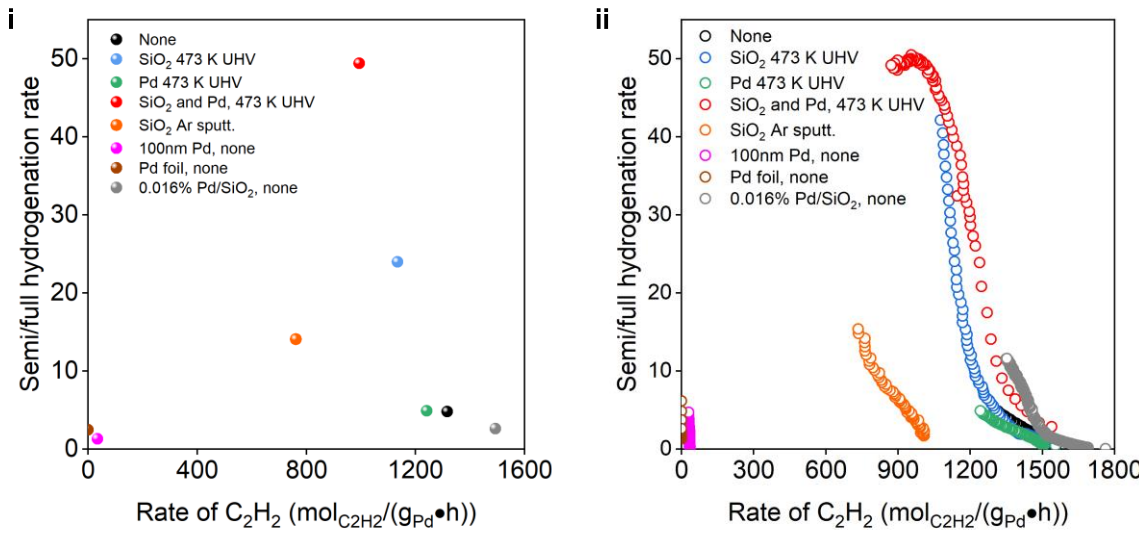

Supplementary Fig. 30c | Comparison on semi/full hydrogenation rate vs. acetylene consumption rate. (**i**) data at 18th hour of catalysis; (**ii**) data from the beginning of the catalysis up to 20 hours. Si − 20 nm SiO_2 − 3 nm Pd were pretreated (None; SiO_2 473 K, UHV; Pd 473 K, UHV; SiO_2 and Pd 473 K, UHV; SiO_2 Ar sputtering.) while 100nm Pd, Pd foil and Pd/SiO_2 powder are without any annealing. Reaction condition: C_2H_2: 0.9 ml/min; H_2: 27 ml/min; N_2: 5.8 ml/min; T = 150°C.

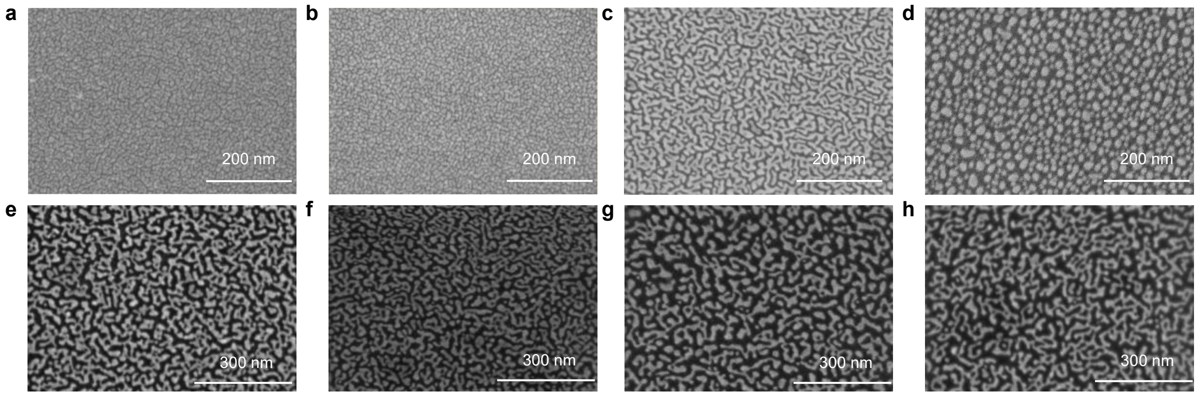

Supplementary Fig. 31 | SEM image of Si − 20 nm SiO_2 − 3 nm Pd with annealing pretreatments on different layers: (**a-d**) fresh samples; (**e-h**) spent samples. (**a, e**) without pretreatment; (**b, f**) SiO_2 473 K, UHV; (**c, g**) Pd 473 K, UHV; (**d, h**) SiO_2 and Pd 473 K, UHV.

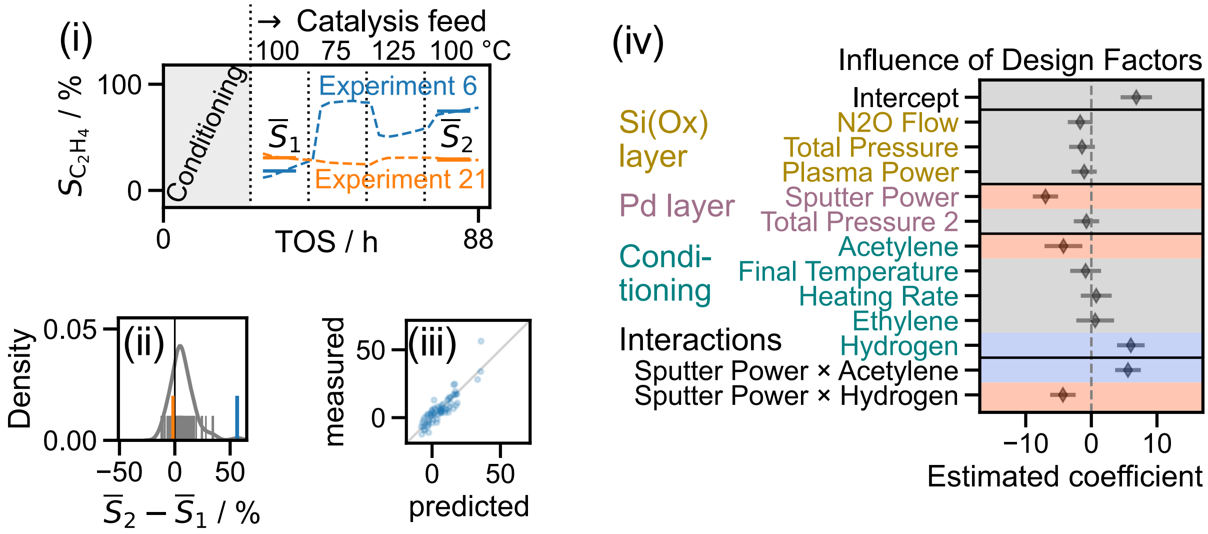

Supplementary Fig. 32 | Design of experiments (DoE) based assessment of catalytic stability of thin film catalysts.

(i) Measurement protocol illustrated for two selected samples (with expt.-IDs 6 and 21). Factor settings are given in **Supplementary Table 9**.

(ii) Distribution of average selectivity differences $\bar{S}_2 - \bar{S}_1$.

(iii) Correlation plot of statistical model predictions of $\bar{S}_2 - \bar{S}_1$ with measured values. A (marginal) coefficient of determination R_m^2 of 0.73 indicates that a major part of the experimental variation can be explained by the model.

(iv) Results of statistical modeling for factor screening. The intercept coefficient of the statistical model (black diamond) provides an estimated value for $\bar{S}_2 - \bar{S}_1$ when all experimental factor settings are set to the central setting over their assessed experimental range. For "Acetylene", "Ethylene", "Hydrogen" (assessed on two levels) we note for completeness that this tentative setting hasn't been realized in practice, see below. The influence of control factors related to Si-/SiO_x intermediate layer formation (3 factors), Pd layer formation (2 factors), and a conditioning phase (5 factors) is further assessed. Factors with negligible influence display 95 % coefficient confidence intervals (gray bars) containing 0 and are indicated by a gray background. Blue/red backgrounds indicate that increases/decreases in $\bar{S}_2 - \bar{S}_1$ can be expected when changing this factor's setting to a higher value. Two active pairwise interaction effects have been identified following the rationale in the SI text. Blue/red backgrounds here indicate that increases/decreases in $\bar{S}_2 - \bar{S}_1$ can be expected when the settings of the involved factors are increased simultaneously. Random intercept contributions occurring in the designed experiment are also discussed in the SI.

Supplementary Tables 1-9

Supplementary Table 1 | Comparison on catalytic results on acetylene hydrogenation reaction between Si − 20 nm SiO_2 − 3 nm Pd and the reported Pd powder catalysts in literature.

Catalysts	Temperature	Flow rate	Feed			TOS	Cat. Mass	Pd loading	Pd mass	Conversion	Selectivity	Productivity	Ref
	℃	ml/min	C_2H_2 (%)	H_2 (%)	C_2H_4 (%)	h	mg	%	mg	%	%	$mol_{C2H4}/(g_{Pd}.h)$	
$InPd_2$	200	30	0.5	5	50	18	28.5	65.0	18.5	90	78	0.014	[26]
$GaPd_2$	200	30	0.5	5	50	18	10.0	75.3	7.53	95	75	0.034	[26]
GaPd	200	30	0.5	5	50	20	40.0	60.4	24.16	86	75	0.009	[30]
Ga_7Pd_3	200	30	0.5	5	50	20	100.0	39.5	39.5	99	71	0.007	[30]
$Pd_{20}Ag_{80}$	200	30	0.5	5	50	20	200.0	20.1	40.2	83	49	0.004	[30]
$GaPd_2$/CNT	200	30	0.5	5	50	20	0.24	4.57	0.01	90	58	19.1	[27]
Mco-PdCu/MgAl-cHT	85	167.5	0.3	0.6	32.9	48	100.0	0.38	0.38	90	85	9.6	[28]
I-PdCu/MgAl-cHT	85	167.5	0.3	0.6	32.9	48	100.0	0.39	0.39	55	75	5.4	[28]
Pd-^{int}B/C	75	50	5	5	-	6.7	5.0	5.0	0.25	82	78	39.5	[24]
Pd-^{int}Li/C	75	50	5	5	-	6.7	5.0	5.0	0.25	85	79	40.9	[24]
PdZn/ZnO	80	30	2	20	-	20	10.0	1.0	0.10	100	96	151.4	[38]
Pd/HT	55	167.5	0.35	0.6	-	30	100.0	0.83	0.83	92	75	135.8	[25]
Pd/MgO	55	167.5	0.35	0.6	-	30	100.0	0.81	0.81	35	69	47.5	[25]
Pd/Al_2O_3	55	167.5	0.35	0.6	-	30	100.0	0.80	0.80	25	65	26.9	[25]
$GaPd/Al_2O_3$	200	30	0.5	5	50	20	75.0	0.0123	0.009	84	82	31.9	[29]
$GaPd_2/Al_2O_3$	200	30	0.5	5	50	20	100.0	0.0055	0.0055	88	72	48.2	[29]
$Pd_2Ga/MgO/MgGa_2O_4$	200	30	0.5	5	50	20	0.047	2.34	0.0011	98	70	188.2	[39]

Pd-In/Al_2O_3	120	40	0.87	3.1	73	12	50.0	0.48	0.24	99	77	303.2	[40]
Pd/MgO	225	50	1	2	-	14	100	0.05	0.05	98	87	22.84	[41]
$AgPd_{0.01}/SiO_2$	160	30	1	20	20	20	30	0.0463	0.0139	68	84	33.02	[42]
$AuPd_{0.025}/SiO_2$	160	30	1	20	20	20	30	0.1016	0.0305	46	65	7.88	[42]
$PdCu_{10}$/MMO	130	220	0.45	0.9	45	14	500	0.06	0.3	64.7	78	4.46	[43]
$PdCu_{68}$/MMO	150	220	0.45	0.9	45	14	500	0.064	0.32	65.5	77	4.18	[43]
$PdCu_{129}$/MgAl-MMO	200	220	0.45	0.9	45	14	500	0.064	0.32	71	98.8	5.81	[43]
Pd_1@Cu-SiW	110	20	0.5	5	50	12	300	0.41	1.23	92	92	0.18	[44]
Pd_1Ni/SiO_2	75	30	1	10	20	12	20	0.038	0.0076	91	88	84.67	[45]
Pd/C	160	50	0.33	0.66	32.8	20	280	0.97	2.716	93	40	0.06	[46]
Pd_2Sn/C	160	50	0.33	0.66	32.8	20	280	0.97	2.716	98.5	91	0.146	[46]
Pd/$MgAl_2O_4$	120	40	1	5	20	20	10	0.1	0.01	88.5	84	79.65	[47]
Pd/SiO_2	100	68.6	2	4.6	-	20	30	6.21	1.863	53	95	0.99	[48]
Pd@SiO_2	100	68.6	2	4.6	-	16	30	6.53	1.959	7	90	0.12	[48]
$Pd_1/Bi_2O_3/TiO_2$	40	60	1	20	20	20	30	4.9	1.47	63	78	0.54	[49]
Pd/TiO_2	120	45	1	10	20	20	15	0.149	0.0224	96	85	43.91	[50]
Pd_1Cu_1/ND@G	90	30	1	10	20	20	30	0.09	0.027	70	92	19.17	[51]
$AuPd_{0.01}/SiO_2$	160	30	1	20	20	20	30	0.0225	0.0068	18	79	16.8	[32]
$AuPd_{0.025}/SiO_2$	160	30	1	20	20	20	30	0.066	0.0198	48	64	12.47	[32]
$AuPd_{0.1}/SiO_2$	160	30	1	20	20	20	30	0.3353	0.1006	65	50	2.60	[32]
Pd/Al_2O_3	200	30	0.5	5	50	20	20	5	1	44	14	0.025	[52]
Pd_1-Fe/Fe_2O_3	80	90	1	10	20	20	200	0.48	0.96	98.5	97.5	2.41	[53]
Pd_1/ND@G	180	30	1	10	20	20	30	0.11	0.033	99	91	21.94	[33]
Pd_3P/TiO_2	225	60	0.6	1.2	5.4	20	50	1.84	0.92	100	78	0.818	[54]
PdP_2/TiO_2	225	60	0.6	1.2	5.4	20	50	1.90	0.95	100	82	0.832	[54]
Pd/$MgAl_2O_4$	90	120	0.5	5	50	20	25	2.2	0.55	98	15	0.43	[55]
$Pd_3In/MgAl_2O_4$	90	120	0.5	5	50	20	25	2.0	0.5	99	19	0.60	[55]
PdIn/$MgAl_2O_4$	90	120	0.5	5	50	20	25	2.2	0.55	97.5	90	2.56	[55]
**Si – 20 nm SiO_2 – 3 nm Pd**	**150**	**33.7**	**2.67**	**80**	**-**	**20**	**60mm^2**	**3 nm**	**0.00163**	**82**	**76**	**966.1**	**This work**

Supplementary Table 2 | Images of (111) slabs calculated for this study from the top looking-down perspective.

Slab	Clean Pd	Pd+C_2H_2	Pd+C_2H_4
Pd(111)	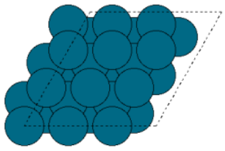	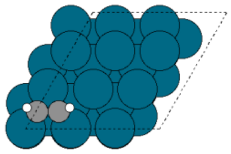	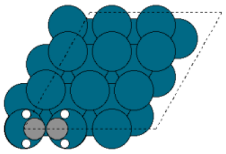
Pd+1C	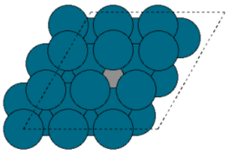	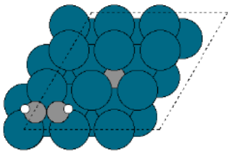	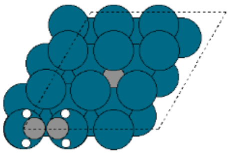
Pd+2C	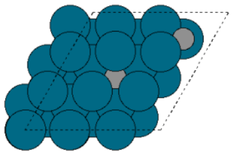	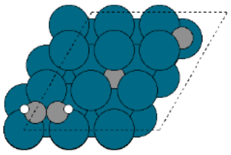	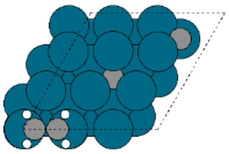
Pd+3C	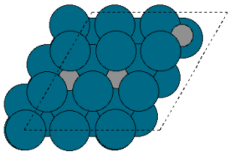	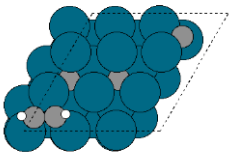	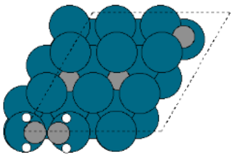
Pd+4C	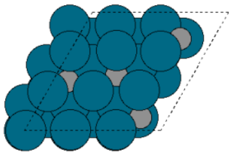	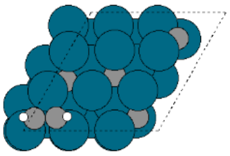	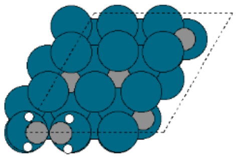
PdAg	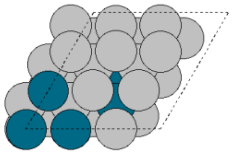	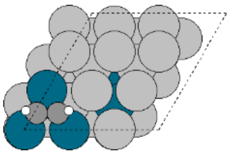	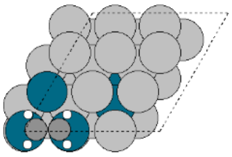

Supplementary Table 3 | Images of (100) slabs calculated for this study from the top looking-down perspective.

Slab	Clean Pd	Pd+C_2H_2	Pd+C_2H_4
Pd	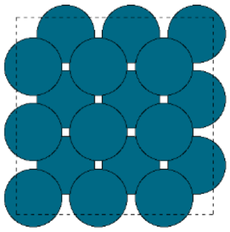	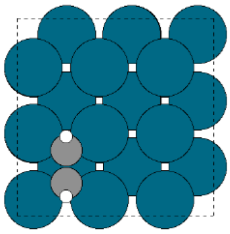	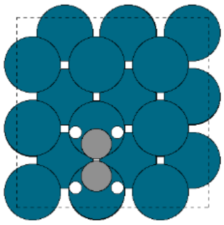
Pd+1C	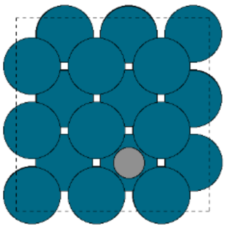	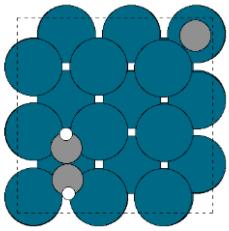	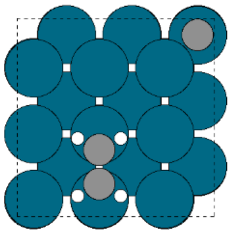
Pd+2C	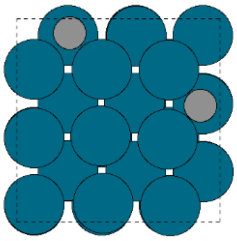	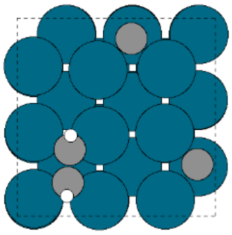	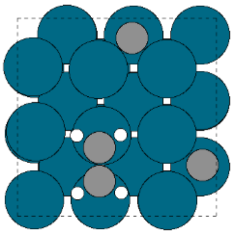
Pd+3C	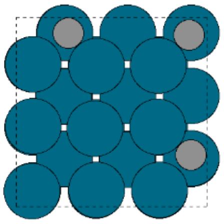	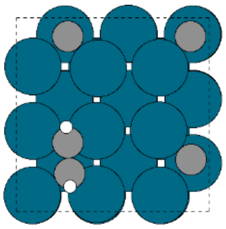	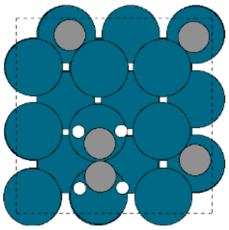

Supplementary Table 4 | Images of (111) slabs calculated for this study from the top looking-down perspective.

Slab	Clean Pd	Pd+C_2H_2	Pd+C_2H_4
Pd+1H	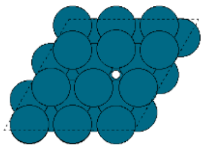	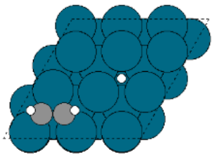	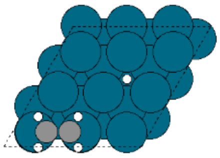
Pd+2H	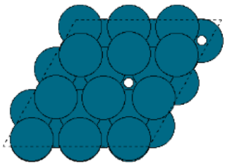	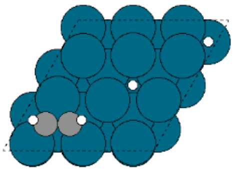	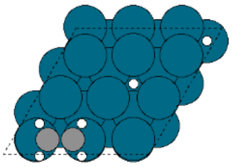
Pd+3H	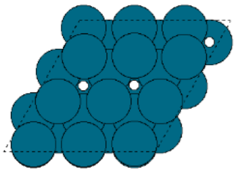	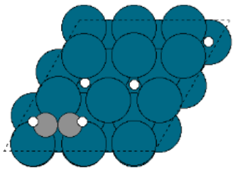	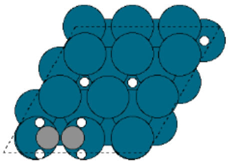
Pd+4H	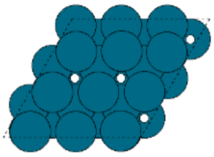	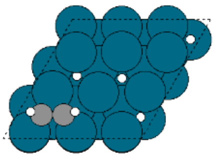	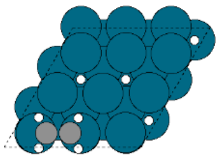

Supplementary Table 5 | Summary of calculated d-band centers relative to the Fermi levels of surfaces used in this study with varying amounts of interstitial carbon and hydrogen atoms. All surfaces are 3x3x4 unit cells in the (111) facet structure. All carbon and hydrogen atoms are in the first interstitial layer.

**Slab**	**Fermi level (eV)**	**d-band center (eV)**
Pd(111)	1.745	-1.797
Pd+1C	0.581	-1.851
Pd+2C	0.679	-1.889
Pd+3C	0.774	-1.977
Pd+4C	0.847	-2.059
PdAg	1.416	-3.939
Pd+1H	1.828	-1.842
Pd+2H	1.850	-1.843
Pd+3H	1.878	-1.847
Pd+4H	1.908	-1.860

Supplementary Table 6a | XPS binding energies and FWHM (given in parentheses) of fitted components used in this study.

Sample	Pd 3*d*	Pd 3*p*	Si 2*p*	C 1*s*
Fig. S6a<br>Si – ~20 nm SiO_2 – ~0.4 nm C				*sp*^{2}= 284.40 eV (0.99)<br>Disordered= 284.88 eV (1.36)<br>C-O= 286.0 eV (2.82)
Fig. S8(a-d) / Fresh Si – ~20 nm SiO_2 – ~0.4 nm C – ~3 nm Pd	Pd^0 = 335.04 eV (1.11)<br><br>Pd:C = 335.60 eV (1.09)<br><br>PdO_x= 336.21 eV (1.48)	O 1*s*: 532.90 eV (SiO_2)<br><br>PdO_x/ PdC_x: 530.95 eV	103. 5 eV	*sp*^{2}= 284.30 eV (1.04)<br>Pd-C=283.80 eV (1.00)<br>C-H = 285.40 eV (1.20)<br>C-O= 287.0 eV (2.00)
Fig. S8(a-d) / After thermal Stability Test<br>Si – ~20 nm SiO_2 – ~0.4 nm C – ~3 nm Pd	Pd^0 = 335.08 eV (1.11)<br><br>Pd:C = 335.5 eV (1.00)	O 1*s*: 532.94 eV (SiO_2)<br><br>PdO_x/ PdC_x: 531.04 eV	103.5eV	*sp*^{2} = 284.31 eV (1.00)<br><br>Pd-C=283.77 (1.09)<br>C- H = 285.40 eV (1.00)<br>C-O=286.38 eV (2.00)
Fig. S8(e-h) / Fresh Si – ~20 nm SiO_2 – ~3 nm Pd	Pd^0 = 335.05 eV (1.11)<br><br>Surface PdO_X = 335.55 eV (1.49)<br><br>PdO_X= 336.09 eV (1.46)	O 1*s*: 532.79 eV (SiO_2/Pd3*p*)<br><br>PdO_X: 531.16 eV	103.5eV	No C1*s* observed
Fig. S8(e-h) / After thermal Stability Test<br><br>Si – ~20 nm SiO_2 – ~3 nm Pd	Pd^0 = 334.90 eV (1.11)<br><br>Surface PdO_X = 335.45 eV (1.60)	O 1*s*: 532.86 eV (SiO_2/Pd3*p*)<br><br>PdOx: 531.19eV	103.5eV	No C1*s* observed
Fig. S9(a-d) / Fresh Si – ~20 nm SiO_2 – ~0.4 nm C – ~3 nm Pd	Pd = 335.00 eV (1.11)<br><br>Pd:C= 335.47 eV (1.18)<br><br>PdO_X= 336.50 eV (1.80)	O 1*s*: 532.7 eV (SiO_2)<br><br>PdO_x: 530.26 eV	103.5eV	Pd-C= 283.90 eV (1.00)<br>*sp*^{2} = 284.36 eV (1.20)<br>C- H = 285.24 eV (1.17)
Fig. S9(a-d) / Spent Si – ~20 nm SiO_2 – ~0.4 nm C – ~3 nm Pd	Pd:C = 335.94 eV (1.18)<br><br>PdO_X= 336.83 eV (2.26)	O 1*s*: 532.7 eV (SiO_2/Pd3*p*)<br><br>PdO_x: 530.26 eV	103.41eV	*sp*^{2} = 284.40 eV (1.00)<br>Disordered = 284.69 eV (1.00)<br>C- H = 285.20 eV (1.00)

Fig. S9(e-h) / Fresh Si – ~20 nm SiO_2 – ~3 nm Pd	Pd^0 = 334.95 eV (1.11)<br>Surface PdO_X= 335.55 eV (1.35)<br>PdO= 336.20 eV (1.57)	Pd 3$p_{3/2}$: 533.3 eV<br>O 1*s*: 532.56 eV (SiO_2/Pd3*p*)<br>PdO_x: 531.17 eV	103.5eV	No C1*s* observed
Fig. S9(e-h) Spent / Si – ~20 nm SiO_2 – ~3 nm Pd	Pd_3Si = 336.63 eV (1.23)	PdO_x/ PdC_x : 532.26 eV	Reduced SiO_2:102.40eV<br>Si2*p*: 98.53	Pd-C= 283.72 eV (1.00)<br>*sp*^{2}= 284.40 eV (1.20)<br>Disordered = 284.73 eV (1.00)<br>C- H = 285.40 eV (1.20)<br>C-O=286.20 eV (1.40)
Fig. S10 / Pd foil	Pd^0= 335.0 eV (1.11)			
Fig. S15a / Si – 20 nm SiO_2 – 3 nm Pd		Pd 3$p_{3/2}$: 532.1 eV (Pd^0) (3.27)<br>O 1*s*: 532.7 eV (SiO_2) (1.09)		
Fig. S15a / Pd foil sputtered 30 min		Pd 3$p_{3/2}$: 532.1 eV (Pd^0) (3.27)		
Fig. S15a / Pd foil, 400°C in O_2		Pd 3$p_{3/2}$: 533.4 eV (PdO) (3.27)<br>O 1*s*(I): 530.8 eV (adsorbed O layer) (1.66)<br>O 1*s*(II): 529.7 eV (surface+bulk oxide) (1.02)<br>O 1*s*(III): 528.9 eV (surface oxide) (1.45)		
Fig. S15a / Pd foil, 600°C in O_2		Pd 3$p_{3/2}$: 532.04 eV (Pd^0) (3.27)<br>O 1*s*(II): 529.7 eV (surface + bulk oxide) (1.40)<br>O 1*s*(III): 528.9 eV (surface oxide) (1.18)		
Fig. S17 / Fresh<br>Si – 20 nm SiO_2 – 3 nm Pd	Pd^0 = 334.9 eV (0.45)<br>Pd:C = 335.4 eV (1.03)	Pd 3$p_{3/2}$: 532.1 eV (Pd^0) (3.27)<br>O 1*s*: 532.7 eV (SiO_2)	103. 4 eV (SiO_2)	Defects:283.9 eV (0.67)<br>*sp*^{2}: 284.4 eV (0.74)<br>Disordered: 284.8 eV (0.90)<br>C-H: 285.3 eV (1.13)<br>C-O: 286.5 eV (1.37)

				C=O: 287.5 eV (1.49)<br>COOH: 289.0 eV (2.00)
Fig. S17 / In-situ<br><br>Si – 20 nm SiO_2 – 3 nm Pd	Pd^0 = 334.7 eV (0.45)<br><br>Pd:C = 335.5 eV (0.83)	Pd $3p_{3/2}$: 532.2 eV (Pd^0) (1.89)	NA	*sp*^{2}: 284.4 eV (0.65)<br><br>Disordered: 284.8 eV (0.80)<br>C- H: 285.3 eV (0.78)
Fig. S17 / Spent<br><br>Si – 20 nm SiO_2 – 3 nm Pd	Pd^0 = 334.7 eV (0.45)<br><br>Pd:C = 335.4 eV (0.75)	Pd $3p_{3/2}$: 532.3 eV (Pd^0) (2.29)	103.4 eV (SiO_2)	*sp*^{2}: 284.4 eV (0.66)<br><br>Disordered: 284.9 eV (0.90)<br><br>C-H: 285.4 eV (0.97)
Fig. S18 / UHV, RT<br><br>Si – 20 nm SiO_2 – 3 nm Pd	Pd^0 = 335.05 eV (0.87)<br>Pd:C = 335.58 eV (0.87)	Pd $3p_{3/2}$: 532.5 eV (Pd^0)	103.2 eV (SiO_2)	284.65 eV
Fig. S18 / H_2, 100°C<br><br>Si – 20 nm SiO_2 – 3 nm Pd	Pd^0 = 335.0 eV (0.87)<br>Pd:C = 335.5 eV (0.87)	Pd $3p_{3/2}$: 532.5 eV (Pd^0)	103.4 eV (SiO_2)	284.65 eV
Fig. S18 / H_2, 200°C<br><br>Si – 20 nm SiO_2 – 3 nm Pd	Pd^0 = 335.0 eV (0.92)<br>Pd:C = 335.5 eV (0.92)	Pd $3p_{3/2}$: 532.5 eV (Pd^0)	103.6 eV (SiO_2)	284.65 eV
Fig. S19 / Pd foil	Pd^0 = 335.0 eV (0.86)<br><br>Surface Pd oxide = 335.5 eV (1.17)	Pd $3p_{3/2}$ = 532.1 eV (Pd^0) (3.27)<br><br>O 1*s* = 528.9 (1.18), 529.8 eV (1.40) (surface Pd oxide)		

Supplementary Table 6b | XPS fitting parameters used in this study.

**Species**	**Lineshape**	**Binding Energy (eV)**
Pd^0 in Pd 3*d*	DS(0.18, 400, 40)	335.0
Pd:C in Pd 3*d*	DS(0.06, 400, 40)	335.5-335.6
Pd 3*p*	DS(0.18, 200,20)	532.2
sp^2 in C 1*s*	DS(0.095, 300, 20)	284.4
Pd:C in C 1*s*	DS(0.005, 300, 20)	283.8-283.9
Disordered C in C 1*s*	DS(0.005, 300, 20)	284.80
C-H in C 1*s*	DS(0.005, 300, 20)	285.3
C-O in C 1*s*	DS(0.005, 300, 20)	286.5
C=O in C 1*s*	DS(0.005, 300, 20)	287.5

Supplementary Table 6c | Atomic percentages of Pd and C in Si − 20 nm SiO_2 − 3 nm Pd before and after operando measurements in 1 mbar C_2H_2+H_2 at T = 100 °C.

Sample	**Pd^0 %**	**Pd:C %**	**C %**	**Si %**
Fresh	5.70	27.6	65.9	0.8
Operando	0.20	3.90	95.9	NA
Spent	0.13	2.77	96.9	0.2

Supplementary Table 6d | Chemical compositions of the samples presented in this study, calculated from XPS measurements in UHV and RT. Kinetic energy of the photoelectrons is 700 eV.

**Sample**	**Pd_2Si %**	**Pd^0 %**	**Pd:C %**	**C %**	**Si %**
Si − ~1 nm Pd	26.0	x	x	16.3	57.7
Si − ~3 nm Pd	22.5	x	x	16.4	61.1
Si − ~11 nm Pd	x	18.9	9.3	40.7	31.1
Si − 20 nm SiO_2 – 3 nm Pd	x	42.4	14.1	41.4	2.1

Supplementary Table 7 | Acetylene hydrogenation rate and productivity of Si – 20 nm SiO_2 – 3 nm Pd (LCC Pd) and reference materials. Consumption rate of C_2H_2 and productivities of C_2H_4, C_2H_6 and C_4H_{10} were measured after 18 hours time-on-stream. Material system: LCC Pd: 3mm x 20mm, 100nm Pd: 3mm x 20mm, Pd foil: 3mm x 20mm, double side, 0.016% Pd@SiO_2 powder: 9 mg, 0.079% Pd@SiO_2 powder: 1.6 mg. Reaction condition: C_2H_2: 0.9 ml/min; H_2: 27 ml/min; N_2: 5.8 ml/min; T = 150°C.

Sample	C_2H_2	C_2H_4	C_2H_6	C_4H_{10}	Semi/full hydrogenation	C_2H_2	C_2H_4	C_2H_6	C_4H_{10}
	mol/g_{Pd}/h	mol/g_{Pd}/h	mol/g_{Pd}/h	mol/g_{Pd}/h	-	mol/m^2/h	mol/m^2/h	mol/m^2/h	mol/m^2/h
LCC Pd (without SiO_2)	negligible	negligible	negligible	negligible	-	negligible	negligible	negligible	negligible
LCC Pd	1316	1059	219	19	4.8	35.8	28.8	6.0	0.5
LCC Pd (SiO_2 473 K, UHV)	1135	1045	44	23	24.0	30.9	28.4	1.2	0.6
LCC Pd (Pd 473 K, UHV)	1242	1002	205	18	4.9	33.8	27.3	5.6	0.5
LCC Pd (SiO_2 473 K, UHV, Pd 473 K, UHV)	993	929	19	23	49.4	27.0	25.3	0.5	0.6
LCC Pd (SiO_2 573 K, H_2)	1188	1072	71	22	15.0	32.3	29.2	1.9	0.6
LCC Pd (SiO_2, Ar sputtering)	762	695	49	9	14.1	20.7	18.9	1.3	0.3
100 nm Pd	35.1	19.8	14.9	0.2	1.3	41.8	23.6	17.7	0.2
Pd foil	0.02	0.015	0.006	0.0001	2.5	20.2	15.2	6.1	0.1
0.016% Pd@SiO_2	1493	1044	400	25	2.6	-	-	-	-
0.079% Pd@SiO_2	1993	1428	418	73	3.4	-	-	-	-

Supplementary Table 8 | Acetylene hydrogenation rate and productivity of Si − 20 nm SiO_2 − 3 nm Pd (LCC Pd) and reference materials. Consumption rate of C_2H_2 and productivities of C_2H_4, C_2H_6 and C_4H_{10} were measured over 18 hours time-on-stream. Material system: LCC Pd: 3mm x 20mm, 100nm Pd: 3mm x 20mm, Pd foil: 3mm x 20mm, double side, 0.016% Pd@SiO_2 powder: 9 mg, 0.079% Pd@SiO_2 powder: 1.6 mg. Reaction condition: C_2H_2: 0.9 ml/min; H_2: 27 ml/min; N_2: 5.8 ml/min; T = 150°C.

Sample	C_2H_2	C_2H_4	C_2H_6	C_4H_{10}	Semi/full hydrogenation	C_2H_2	C_2H_4	C_2H_6	C_4H_{10}
	mol/g_{Pd}	mol/g_{Pd}	mol/g_{Pd}	mol/g_{Pd}	-	mol/m^2	mol/m^2	mol/m^2	mol/m^2
LCC Pd (without SiO_2)	negligible	negligible	negligible	negligible	-	negligible	negligible	negligible	negligible
LCC Pd	26048	16133	9471	222	1.7	708.5	438.8	257.6	6.0
LCC Pd (SiO_2 473 K, UHV)	23542	17739	5185	309	3.4	640.3	482.5	141.0	8.4
LCC Pd (Pd 473 K, UHV)	24913	15952	8573	194	1.9	677.6	433.9	233.2	5.3
LCC Pd (SiO_2 473 K, UHV, Pd 473 K, UHV)	20633	18804	1011	409	18.6	561.2	511.5	27.5	11.1
LCC Pd (SiO_2 573 K, H_2)	25089	18153	6504	216	2.8	682.4	493.8	176.9	5.9
LCC Pd (SiO_2, Ar sputtering)	16342	13138	3002	101	4.4	444.5	357.4	81.7	2.7
100 nm Pd	635	259	372	2	0.7	755.7	308.2	442.7	2.4
Pd foil	0.374	0.240	0.130	0.002	1.8	378.1	242.6	131.4	2.0
0.016% Pd@ SiO_2	28376	13667	13735	487	1.0	-	-	-	-
0.079% Pd@ SiO_2	36152	22128	11911	1063	1.9	-	-	-	-

Supplementary Table 9 | Experimental Design in Factors A-I.2 and response property $\overline{S}_2 - \overline{S}_1$[%]. For each collected datapoint, we further list the Run-ID for the run in the multireactor setup and the order in which these runs were executed. Each deposited thin film catalyst is identified by a Film-ID. If thin film catalysts were jointly processed in the same Si-/SiOx interlayer deposition step or Pd deposition step, every other Film-ID is listed in columns "Joint Si(Ox)" and "Joint Pd" respectively. Column "Sample number" contains the sample number of the spent sample in the FHI-Database (from which all further information is accessible).

Expt-ID	Sample number	$\overline{S}_2 - \overline{S}_1$ [%]	A	B	C	D	E	F	G	H	I.1	I.2	Run-ID	Order of measurment	Reactor	Film-ID	Joint Si(O_x)	Joint Pd
1	S36940	28.09	0	0	1	0	-1	-1	-1	1	-1	1	1	3	7	13		12
2	S36939	6.11	0	0	-1	0	1	-1	-1	1	-1	1	1	3	6	14		15
3	S36938	8.96	1	0	-1	0	0	-1	-1	1	-1	1	1	3	5	06		02
4	S36937	6.93	0	0	0	-1	1	-1	-1	1	-1	1	1	3	4	38	41	
5	S36936	6.62	0	0	1	1	0	-1	-1	1	-1	1	1	3	3	11	09	10
6	S36935	56.33	0	-1	0	0	-1	-1	-1	1	-1	1	1	3	2	24	22	25
7	S36841	10.76	-1	1	0	0	0	-1	-1	1	-1	1	1	3	1	17		16
8	S37507	-11.00	-1	0	0	0	1	1	-1	-1	-1	1	2	10	7	34	32	
9	S37506	-1.26	0	-1	1	0	0	1	-1	-1	-1	1	2	10	6	01		07
10	S37505	12.84	0	1	0	0	-1	1	-1	-1	-1	1	2	10	5	25	23	24
11	S37504	1.99	1	0	0	1	0	1	-1	-1	-1	1	2	10	4	31	29	
12	S37503	-3.53	0	0	1	-1	0	1	-1	-1	-1	1	2	10	3	09	11	28
13	S37502	-5.41	0	0	-1	0	1	1	-1	-1	-1	1	2	10	2	14		15
14	S37501	10.47	0	0	-1	0	-1	1	-1	-1	-1	1	2	10	1	12	10	13
15	S36739	-6.67	0	1	0	0	-1	-1	-1	-1	1	-1	3	1	7	25	23	24
16	S36740	-8.9	0	-1	0	0	-1	-1	-1	-1	1	-1	3	1	6	24	22	25
17	S36741	-2.02	1	0	0	1	0	-1	-1	-1	1	-1	3	1	5	31	29	
18	S36742	-2.19	0	0	1	0	1	-1	-1	-1	1	-1	3	1	4	15		14
19	S36743	2.21	-1	0	1	0	0	-1	-1	-1	1	-1	3	1	3	05		41
20	S36744	3.02	-1	0	-1	0	0	-1	-1	-1	1	-1	3	1	2	04		40
21	S36745	-1.67	0	0	-1	0	1	-1	-1	-1	1	-1	3	1	1	14		15
22	S37059	10.04	-1	0	0	1	0	-1	1	-1	-1	1	4	5	7	30	28	
23	S37058	5.82	1	-1	0	0	0	-1	1	-1	-1	1	4	5	6	18		19
24	S37057	7.08	0	1	0	1	0	-1	1	-1	-1	1	4	5	5	23	25	22
25	S37056	5.72	0	-1	0	0	1	-1	1	-1	-1	1	4	5	4	26	20	27
26	S37055	19.32	0	0	-1	0	-1	-1	1	-1	-1	1	4	5	3	12	10	13
27	S37054	4.74	0	1	0	-1	0	-1	1	-1	-1	1	4	5	2	21	27	20
28	S37053	5.04	0	0	1	-1	0	-1	1	-1	-1	1	4	5	1	09	11	28
29	S36812	16.86	1	0	0	-1	0	1	1	1	-1	1	5	2	7	29	31	
30	S36811	33.98	0	-1	0	0	-1	1	1	1	-1	1	5	2	6	24	22	25
31	S36810	24.16	0	1	0	1	0	1	1	1	-1	1	5	2	5	23	25	22
32	S36809	17.16	-1	0	1	0	0	1	1	1	-1	1	5	2	4	05		41
33	S36808	12.89	0	0	0	-1	1	1	1	1	-1	1	5	2	3	38	41	
34	S36807	17.53	0	-1	0	1	0	1	1	1	-1	1	5	2	2	22	24	23
35	S36806	16.93	0	1	0	1	0	1	1	1	-1	1	5	2	1	23	25	22
36	S37226	-4.60	1	-1	0	0	0	1	1	-1	1	-1	6	7	7	18		19
37	S37225	-3.91	0	0	-1	0	1	1	1	-1	1	-1	6	7	6	14		15
38	S37224	-3.82	1	1	0	0	0	1	1	-1	1	-1	6	7	5	19		18
39	S37223	-3.70	0	0	1	0	1	1	1	-1	1	-1	6	7	4	15		14
40	S37222	-6.54	-1	0	0	1	0	1	1	-1	1	-1	6	7	3	30	28	
41	S37221	-6.62	0	0	0	-1	-1	1	1	-1	1	-1	6	7	2	36	40	
42	S37220	-9.33	-1	0	0	0	-1	1	1	-1	1	-1	6	7	1	32	34	33
43	S37323	2.68	0	-1	0	1	0	0	0	-1	1	1	7	9	7	22	24	23
44	S37322	0.82	0	0	1	-1	0	0	0	-1	1	1	7	9	6	09	11	28
45	S37321	1.10	-1	-1	0	0	0	0	0	-1	1	1	7	9	5	16		17
46	S37320	-12.24	1	0	0	0	-1	0	0	-1	1	1	7	9	4	33		32
47	S37319	3.41	-1	0	-1	0	0	0	0	-1	1	1	7	9	3	04		40
48	S37318	-0.01	1	1	0	0	0	0	0	-1	1	1	7	9	2	19		18
49	S37317	1.69	0	1	0	0	1	0	0	-1	1	1	7	9	1	27	21	26
50	S36970	7.22	0	0	1	1	0	-1	1	1	1	-1	8	4	7	11	09	10
51	S36969	6.09	0	-1	0	-1	0	-1	1	1	1	-1	8	4	6	20	26	21
52	S36968	13.84	-1	0	0	-1	0	-1	1	1	1	-1	8	4	5	28	30	09
53	S36967	6.08	1	0	1	0	0	-1	1	1	1	-1	8	4	4	07		01
54	S36966	8.02	-1	0	-1	0	0	-1	1	1	1	-1	8	4	3	04		40
55	S36965	6.50	0	0	-1	1	0	-1	1	1	1	-1	8	4	2	10	12	11
56	S36964	4.01	1	1	0	0	0	-1	1	1	1	-1	8	4	1	19		18
57	S37177	4.90	1	0	0	-1	0	1	-1	1	1	-1	9	6	7	29	31	
58	S37176	3.30	0	-1	0	0	1	1	-1	1	1	-1	9	6	6	26	20	27
59	S37175	5.00	0	0	0	1	1	1	-1	1	1	-1	9	6	5	39		
60	S37174	8.05	0	0	1	1	0	1	-1	1	1	-1	9	6	4	11	09	10
61	S37173	14.68	0	0	-1	0	-1	1	-1	1	1	-1	9	6	3	12	10	13
62	S37172	9.08	-1	1	0	0	0	1	-1	1	1	-1	9	6	2	17		16
63	S37171	15.15	0	0	0	-1	-1	1	-1	1	1	-1	9	6	1	36	40	
64	S37247	3.43	1	1	0	0	0	0	0	1	1	1	10	8	7	19		18
65	S37246	3.70	0	1	-1	0	0	0	0	1	1	1	10	8	6	02		06

66	S37245	5.46	0	-1	0	1	0	0	0	1	1	1	10	8	5	22	24	23
67	S37244	24.81	-1	0	0	0	-1	0	0	1	1	1	10	8	4	32	34	33
68	S37243	3.94	-1	-1	0	0	0	0	0	1	1	1	10	8	3	16		17
69	S37242	3.65	1	0	1	0	0	0	0	1	1	1	10	8	2	07		01
70	S37241	2.17	0	0	0	-1	1	0	0	1	1	1	10	8	1	38	41	

Supplementary Sample Table

Supplementary Table 10 | Samples used in this work. (Sample numbers are registered in FHI data archive https://ac.archive.fhi.mpg.de)

Sample number (article)	Sample number (FHI Database)	Composition	Preparation/treatment	Figure/Table	Panel/row
S1	S37046	Si - ~20nm SiO_2 - ~0.4nm C - ~3nm Pd	SiO_2 – thermal oxidation;<br>C – Thermal Evaporation;<br>Pd – Thermal evaporation.	Fig. 3	b, XPS
S1	S37356	Si - ~20nm SiO_2 - ~0.4nm C - ~3nm Pd	SiO_2 – thermal oxidation;<br>C – Thermal Evaporation;<br>Pd – Thermal evaporation.	Fig. 3	d, catalysis<br>e, catalysis
S2	S38086	Si - ~20nm SiO_2 - ~2nm C - ~3nm Pd	SiO_2 – thermal oxidation;<br>C – Thermal evaporation;<br>Pd – Thermal evaporation.	Fig. 3	c, XPEEM, XPS
S3	S37354	Si - ~20nm SiO_2 - ~3nm Pd	SiO_2 – thermal oxidation;<br>Pd Thermal evaporation.	Fig. 3	b, XPS<br>d, catalysis
S4	S38055	Si - 20nm SiO_2 - ~3nm Pd	SiO_2 – PECVD;<br>Pd – sputtering.	Fig. 4	a, XPS<br>b, XPS
S4	S35512	Si - 20nm SiO_2 - ~3nm Pd	SiO_2 – PECVD;<br>Pd – sputtering.	Fig. 4	c, XPEEM<br>d, XPS
S4	S35673	Si - 20nm SiO_2 - ~3nm Pd	SiO_2 – PECVD;<br>Pd – sputtering.	Fig. 4	e, EDS<br>g, TEM
S4	S35699	Si - 20nm SiO_2- ~3nm Pd	SiO_2 – PECVD;<br>Pd – sputtering;<br>After C_2H_2 hydrogenation	Fig. 4	f, EDS<br>g, TEM
S4	S35652	Si - 20nm SiO_2 - ~3nm Pd	SiO_2 – PECVD;<br>Pd – sputtering.	Fig. 4	h, catalysis<br>i, catalysis
S4	S38055	Si - 20nm SiO_2 - ~3nm Pd	SiO_2 – PECVD;<br>Pd – sputtering.	Fig. 5	a, XPS<br>b, XPS
S4	S35268	Si - 20nm SiO_2 - ~3nm Pd	SiO_2 – PECVD;<br>Pd – sputtering.	Table 1	1, catalysis

S4	S35289	Si - 20nm SiO_2 - ~3nm Pd	SiO_2 – PECVD, 473K UHV; Pd – sputtering.	Table 1	2, catalysis
S4	S35247	Si - 20nm SiO_2 - ~3nm Pd	SiO_2 – PECVD; Pd – sputtering, 473K UHV.	Table 1	3, catalysis
S4	S35252	Si - 20nm SiO_2 - ~3nm Pd	SiO_2 – PECVD, 473K UHV; Pd – sputtering, 473K UHV.	Table 1	4, catalysis
S4	S35291	Si - 20nm SiO_2 - ~3nm Pd	SiO_2 – PECVD, Ar sputter; Pd – sputtering.	Table 1	5, catalysis
S5	S37378	Si - 20nm SiO_2 - 100nm Pd	SiO_2 – PECVD; Pd – sputtering.	Table 1	6, catalysis
S6	S35009	Pd foil	Good Fellow, commercial.	Table 1	7, catalysis
S7	S36386	0.016% Pd@SiO_2 powder	Wetness impregnation	Table 1	8, catalysis
S8 and more	See DoE	Si - Xnm SiO_2 - Xnm Pd	SiO_2 – PECVD; Pd – sputtering; Various pretreatments.	Fig. 6	DoE

Samples in Supporting Information

S9	S38085	Si - ~20nm SiO_2 – ~0.4nm C	SiO_2 – thermal oxidation; C – Thermal Evaporation.	Fig. S6	a, XPS
S1	S37046	Si - ~20nm SiO_2 - ~0.4nm C - ~3nm Pd	SiO_2 – thermal oxidation; C – Thermal Evaporation; Pd – Thermal evaporation.	Fig. S6	b, XPS
S1	S38596	Si - ~20nm SiO_2 - ~0.4nm C - ~3nm Pd	SiO_2 – thermal oxidation: C Thermal Evaporation; Pd – Thermal evaporation, 423K, UHV;	Fig. S6	c, XPS
S3	S37354	Si - ~20nm SiO_2 - ~3nm Pd	SiO_2 – thermal oxidation; Pd Thermal evaporation.	Fig. S7	a, SEM
S1	S37046	Si - ~20nm SiO_2 - ~0.4nm C - ~3nm Pd	SiO_2 – thermal oxidation; C – Thermal evaporation; Pd – Thermal evaporation.	Fig. S7	b, STM

S2	S38086	Si - ~20nm SiO_2 - ~2nm C - ~3nm Pd	SiO_2 – thermal oxidation;<br>C – Thermal evaporation;<br>Pd – Thermal evaporation.	Fig. S7	c, LEEM<br>d, XPEEM<br>e, XPS
S1	S38596	Si - ~20nm SiO_2 - ~0.4nm C - ~3nm Pd	SiO_2 – thermal oxidation;<br>C – Thermal evaporation;<br>Pd – Thermal evaporation, 423K, UHV.	Fig. S8	top: XPS
S3	S38595	Si - ~20nm SiO_2 - ~3nm Pd	SiO_2 – thermal oxidation;<br>Pd Thermal evaporation;<br>Thermal stability.	Fig. S8	bottom: XPS
S1	S37046	Si - ~20nm SiO_2 - ~0.4nm C - ~3nm Pd	SiO_2 – thermal oxidation;<br>C – Thermal evaporation;<br>Pd – Thermal evaporation.	Fig. S9	top: XPS
S3	S37354	Si - ~20nm SiO_2 - ~3nm Pd	SiO_2 – thermal oxidation;<br>Pd Thermal evaporation.	Fig. S9	bottom: XPS
S6	S37907	Pd foil	commercial	Fig. S10	XPS
S10	S34906	Si - ~3nm Pd	Pd – sputtering	Fig. S11	TEM, EDS
S11	S34909	Si - ~10 nm Pd	Pd – sputtering	Fig. S12	a, catalysis
S10	S34906	Si - ~3nm Pd	Pd – sputtering	Fig. S12	b, catalysis
S10	S34906	Si - ~3nm Pd	Pd – sputtering	Fig. S13	a,b, XPS
S11	S34909	Si - ~10 Pd	Pd – sputtering	Fig. S13	a,b,c,d, XPS
S12	S34934	Si - ~1nm Pd	Pd – sputtering	Fig. S13	a,b, XPS
S4	S38055	Si - 20nm SiO_2 - ~3nm Pd	SiO_2 – PECVD;<br>Pd – sputtering.	Fig. S14	XPS
S10	S34906	Si - ~3nm Pd	Pd – sputtering	Fig. S14	XPS
S6	S35009	Pd foil	Good Fellow, commercial.	Fig. S15	a, XPS
S4	S38055	Si - 20nm SiO_2 - ~3nm Pd	SiO_2 – PECVD;<br>Pd – sputtering.	Fig. S15	a, XPS
S10	S34906	Si - ~3nm Pd	Pd – sputtering	Fig. S15	b, XPS
S11	S34909	Si - ~10nm Pd	Pd – sputtering	Fig. S15	b, XPS
S12	S34934	Si - ~1nm Pd	Pd – sputtering	Fig. S15	b, XPS
S13	S34908	Si - 200nm SiO_2 - ~10nm Pd	SiO_2 – PECVD;	Fig. S16	XPS

			Pd – sputtering.		
S4	S38055	Si - 20nm SiO_2 - ~3nm Pd	SiO_2 – PECVD;<br>Pd – sputtering.	Fig. S17	XPS
S4	S38055	Si - 20nm SiO_2 - ~3nm Pd	SiO_2 – PECVD;<br>Pd – sputtering.	Fig. S18	XPS
S6	S35009	Pd foil	Good Fellow, commercial.	Fig. S19	XPS
S4	S38055	Si - 20nm SiO_2 - ~3nm Pd	SiO_2 – PECVD;<br>Pd – sputtering.	Table S6c	XPS
S12	S34934	Si - ~1nm Pd	Pd – sputtering	Table S6d	1, XPS
S10	S34906	Si - ~3nm Pd	Pd – sputtering	Table S6d	2, XPS
S11	S34909	Si - ~10nm Pd	Pd – sputtering	Table S6d	3, XPS
S4	S38055	Si - 20nm SiO_2 - ~3nm Pd	SiO_2 – PECVD;<br>Pd – sputtering.	Table S6d	4, XPS
S4	S35290	Si - 20nm SiO_2 - ~3nm Pd	SiO_2 – PECVD;<br>Pd – sputtering.	Fig. S20a	SEM
S4	S35673	Si - 20nm SiO_2 - ~3nm Pd	SiO_2 – PECVD;<br>Pd – sputtering.	Fig. S20b	TEM
S4	S35699	Si - 20nm SiO_2- ~3nm Pd	SiO_2 – PECVD;<br>Pd – sputtering;<br>After C_2H_2 hydrogenation	Fig. S20c	TEM
S4	S35673	Si - 20nm SiO_2 - ~3nm Pd	SiO_2 – PECVD;<br>Pd – sputtering.	Fig. S21	a, catalysis<br>b, SEM
S4	S35512	Si - 20nm SiO_2 - ~3nm Pd	SiO_2 – PECVD;<br>Pd – sputtering.	Fig. S22	a, LEEM<br>b, XPEEM<br>c, XPS
S6	S35009	Pd foil	Good Fellow, commercial.	Fig. S23	catalysis
S7	S36386	0.016% Pd@SiO_2 powder	Wetness impregnation	Fig. S23	catalysis
S4	S35652	Si - 20nm SiO_2 - ~3nm Pd	SiO_2 – PECVD;<br>Pd – sputtering.	Fig. S23	catalysis
S6	S35009	Pd foil	Good Fellow, commercial.	Fig. S24	e,f,g, catalysis
S4	S37784	Si - 20nm SiO_2 - ~3nm Pd	SiO_2 – PECVD;<br>Pd – sputtering.	Fig. S24	a,b,e,f,g, catalysis

S14	S38113	0.079% Pd@SiO_2 powder	Wetness impregnation	Fig. S24	c,d,e,f,g, catalysis
S6	S35009	Pd foil	Good Fellow, commercial.	Fig. S25	a,b,c,d,e,f, catalysis
S7	S36386	0.016% Pd@SiO_2 powder	Wetness impregnation	Fig. S25	f, catalysis
S4	S35652	Si - 20nm SiO_2 - ~3nm Pd	SiO_2 – PECVD;<br>Pd – sputtering.	Fig. S25	a,b,c,d,e,f,g, catalysis
S14	S38113	0.079% Pd@SiO_2 powder	Wetness impregnation	Fig. S25	f, catalysis
S5	S37378	Si - 20nm SiO_2 - 100nm Pd	SiO_2 – PECVD;<br>Pd – sputtering.	Fig. S25	a,b,c,d,e,f, catalysis
S15	S35065	Si - 200nm SiO_2 - ~3nm Pd	SiO_2 – PECVD;<br>Pd – sputtering.	Fig. S26	catalysis
S15	S35065	Si - 200nm SiO_2 - ~3nm Pd	SiO_2 – PECVD;<br>Pd – sputtering.	Fig. S27	catalysis
S4	S38055	Si - 20nm SiO_2 - ~3nm Pd	SiO_2 – PECVD;<br>Pd – sputtering.	Fig. S28	a,b, adsorption
S6	S35009	Pd foil	Good Fellow, commercial.	Fig. S28	b, adsorption
S7	S36386	0.016% Pd@SiO_2 powder	Wetness impregnation	Fig. S28	b, adsorption
S6	S35009	Pd foil	Good Fellow, commercial.	Fig. S29	PM-IRAS
S4	S35268	Si - 20nm SiO_2 - ~3nm Pd	SiO_2 – PECVD;<br>Pd – sputtering.	Fig. S29	PM-IRAS
S4	S35268	Si - 20nm SiO_2 - ~3nm Pd	SiO_2 – PECVD;<br>Pd – sputtering.	Fig. S30a	catalysis
S4	S35289	Si - 20nm SiO_2 - ~3nm Pd	SiO_2 – PECVD, 473K UHV;<br>Pd – sputtering.	Fig. S30a	catalysis
S4	S35247	Si - 20nm SiO_2 - ~3nm Pd	SiO_2 – PECVD;<br>Pd – sputtering, 473K UHV.	Fig. S30a	catalysis
S4	S35252	Si - 20nm SiO_2 ~3nm Pd	SiO_2 – PECVD, 473K UHV;<br>Pd – sputtering, 473K UHV.	Fig. S30a	catalysis
S4	S35291	Si - 20nm SiO_2 - ~3nm Pd	SiO_2 – PECVD, Ar sputter;<br>Pd – sputtering.	Fig. S30a	catalysis
S6	S35009	Pd foil	Good Fellow, commercial.	Fig. S30b	catalysis
S7	S36386	0.016% Pd@SiO_2 powder	Wetness impregnation	Fig. S30b	catalysis
S4	S35652	Si - 20nm SiO_2 - ~3nm Pd	SiO_2 – PECVD;	Fig. S30b	catalysis

			Pd – sputtering.		
S5	S37378	Si - 20nm SiO_2 - 100nm Pd	SiO_2 – PECVD;<br>Pd – sputtering.	Fig. S30b	catalysis
S4	S35268	Si - 20nm SiO_2 - ~3nm Pd	SiO_2 – PECVD;<br>Pd – sputtering.	Fig. S30c	catalysis
S4	S35289	Si - 20nm SiO_2 - ~3nm Pd	SiO_2 – PECVD, 473K UHV;<br>Pd – sputtering.	Fig. S30c	catalysis
S4	S35247	Si - 20nm SiO_2 - ~3nm Pd	SiO_2 – PECVD;<br>Pd – sputtering, 473K UHV.	Fig. S30c	catalysis
S4	S35252	Si - 20nm SiO_2 - ~3nm Pd	SiO_2 – PECVD, 473K UHV;<br>Pd – sputtering, 473K UHV.	Fig. S30c	catalysis
S4	S35291	Si - 20nm SiO_2 - ~3nm Pd	SiO_2 – PECVD, Ar sputter;<br>Pd – sputtering.	Fig. S30c	catalysis
S6	S35009	Pd foil	Good Fellow, commercial.	Fig. S30c	catalysis
S7	S36386	0.016% Pd@SiO_2 powder	Wetness impregnation	Fig. S30c	catalysis
S5	S37378	Si - 20nm SiO_2 - 100nm Pd	SiO_2 – PECVD;<br>Pd – sputtering.	Fig. S30c	catalysis
S4	S35268	Si - 20nm SiO_2 - ~3nm Pd	SiO_2 – PECVD;<br>Pd – sputtering.	Fig. S31	a, SEM
S4	S35289	Si - 20nm SiO_2 - ~3nm Pd	SiO_2 – PECVD, 473K UHV;<br>Pd – sputtering.	Fig. S31	b, SEM
S4	S35247	Si - 20nm SiO_2 - ~3nm Pd	SiO_2 – PECVD;<br>Pd – sputtering, 473K UHV.	Fig. S31	c, SEM
S4	S35252	Si - 20nm SiO_2 - ~3nm Pd	SiO_2 – PECVD, 473K UHV;<br>Pd – sputtering, 473K UHV.	Fig. S31	d, SEM
S4	S35612	Si - 20nm SiO_2 - ~3nm Pd	SiO_2 – PECVD;<br>Pd – sputtering;<br>After C_2H_2 hydrogenation.	Fig. S31	e, SEM
S4	S35613	Si - 20nm SiO_2 - ~3nm Pd	SiO_2 – PECVD, 473K UHV;<br>Pd – sputtering;<br>After C_2H_2 hydrogenation.	Fig. S31	f, SEM
S4	S35182	Si - 20nm SiO_2 - ~3nm Pd	SiO_2 – PECVD;<br>Pd – sputtering, 473K UHV;	Fig. S31	g, SEM

			After C_2H_2 hydrogenation.		
S4	S35953	Si - 20nm SiO_2 - ~3nm Pd	SiO_2 – PECVD, 473K UHV; Pd – sputtering, 473K UHV; After C_2H_2 hydrogenation.	Fig. S31	h, SEM
S10	S34906	Si - ~3nm Pd	Pd – sputtering	Tables S7,8	1, catalysis
S4	S35268	Si - 20nm SiO_2 - ~3nm Pd	SiO_2 – PECVD; Pd – sputtering.	Tables S7,8	2, catalysis
S4	S35289	Si - 20nm SiO_2 - ~3nm Pd	SiO_2 – PECVD, 473K UHV; Pd – sputtering.	Tables S7,8	3, catalysis
S4	S35247	Si - 20nm SiO_2 - ~3nm Pd	SiO_2 – PECVD; Pd – sputtering, 473K UHV.	Tables S7,8	4, catalysis
S4	S35252	Si - 20nm SiO_2 - ~3nm Pd	SiO_2 – PECVD, 473K UHV; Pd – sputtering, 473K UHV.	Tables S7,8	5, catalysis
S4	S35288	Si - 20nm SiO_2 - ~3nm Pd	SiO_2 – PECVD, H_2 sputter at 573K; Pd – sputtering.	Tables S7,8	6, catalysis
S4	S35291	Si - 20nm SiO_2 - ~3nm Pd	SiO_2 – PECVD, Ar sputter; Pd – sputtering.	Tables S7,8	7, catalysis
S5	S37378	Si - 20nm SiO_2 - 100nm Pd	SiO_2 – PECVD; Pd – sputtering.	Tables S7,8	8, catalysis
S6	S35009	Pd foil	Good Fellow, commercial.	Tables S7,8	9, catalysis
S7	S36386	0.016% Pd@SiO_2 powder	Wetness impregnation	Tables S7,8	10, catalysis
S14	S38113	0.079% Pd@SiO_2 powder	Wetness impregnation	Tables S7,8	11, catalysis
S8 and more	See DoE	Si - SiO_2 - Pd	SiO_2 – PECVD; Pd – sputtering; Various pretreatments.	Fig. S32,33,34	DoE
S8 and more	See DoE	Si - SiO_2 - Pd	SiO_2 – PECVD; Pd – sputtering; Various pretreatments.	Table S9	DoE

Supplementary Method for DoE

Preparation of thin film catalysts

The experimental design in **Supplementary Table 9** contained 35 unique combinations of settings A-E and hence the respective number of LCCs wafers were prepared. The catalysts were synthesized using commercially relevant deposition technologies namely physical vapor deposition (PVD, sputtering) and plasma enhanced chemical vapor deposition (PECVD). Czochralsky grown, 6- and 8-inch silicon wafers were used as substrate material. To separate the wafer into individual sample pieces, we employed UV laser ablation (Keyence MD-U) to structure the backside of the wafers. After structuring the samples were separated into 5x5cm^2 plates and wet chemically cleaned afterwards. For cleaning an ozone-based cleaning process (DI water with an O_3 concentration of 20ppm at RT) was used. Final native oxide was removed immediately before coating of the buffer layers by diluted HF (1%). The buffer layers were deposited by PECVD using 60MHz excitation at varying powers (control factor "Plasma Power") using monosilane (SiH_4) at a fixed flow rate of 4 sccm and nitrous oxide (N_2O) at varying flow rates (control factor "N_2O-Flow") as precursor gases. During deposition, the substrates were placed on a heated chuck with a temperature of 400°C, while varying chamber pressures were applied (control factor "Total Pressure").

After buffer deposition, samples were transferred into a magnetron sputter coater (PREVAC) for palladium deposition at RT. Films were deposited using a 5N 2-in palladium target, chamber pressure (control factor "Total Pressure 2") and 13.56 MHz RF plasma power (control factor "Sputter Power") were varied according to the experimental plan.

Catalytic testing of thin film catalysts in acetylene hydrogenation

The acetylene hydrogenation experiments were carried out in a commercial parallel reactor setup (Integrated Lab Solutions) with 8 fixed bed quartz reactors at atmospheric pressure. Reactors 1 to 7 were used for the thin film samples, while reactor 8 was empty and served as blank measurement.

The sample size was a 20 mm x 5 mm section of the wafer with the thin film deposited on both sides. A height of 20 mm was selected to ensure that the sample will be inside the isothermal zone of the reactor oven. A sample width of 5 mm was chosen so that the sample can be easily loaded into the quartz reactor, which has an internal diameter of 6 mm.

Each sample measurement can be divided in two stages, the conditioning phase and the steady state phase. The conditioning phase is part of the DoE with varying temperature (100 °C, 125 °C or 150°C), heating rate (0.5 K/min, 2.75 K/min or 5 K/min), hydrogen flow per reactor (0 ml/min, 0.6 ml/min or 6 ml/min), and hydrocarbon flow per reactor (acetylene with 0.2 ml/min, ethylene with 2 ml/min or acetylene plus ethylene). The total flow per reactor was held constant at 20 ml/min, balanced with nitrogen. The temperature was held for 24 hours and then cooled down if necessary to 100°C, which ends the conditioning phase. The steady state phase is then performed to measure the catalytic performance of the different thin film samples. The following reaction conditions were applied: temperature steps in the order 100 °C → 75 °C → 125 °C → 100 °C with holding time of 16 hours per temperature, heating rate of 0.5 K/min, 6 ml/min hydrogen flow per reactor, 0.2 ml/min acetylene flow per reactor, and 13.8 ml/min nitrogen flow per reactor. **Supplementary Fig. 33** shows a graphical representation of the measurement protocol.

Under these DoE experimental conditions and over the investigated temperature range (75 °C to 125 °C), roughly 60% of the measurements show a conversion of almost 100%, while none of the samples showed catalytic inactivity (conversion $\gtrsim$ 15 %).

Conversion of acetylene and selectivity of products are calculated based on the equations shown in the main article.

The early and late TOS averages $\bar{S}_1$ and $\bar{S}_2$ over ethylene selectivities S_{C2H4} are computed over at least 4 data-points, extracted from the two 100 °C windows included in the measurement protocol (meaning right before the 75 °C window and right before the end of the protocol respectively). All such measurements were taken at temperatures between 99 and 101 °C

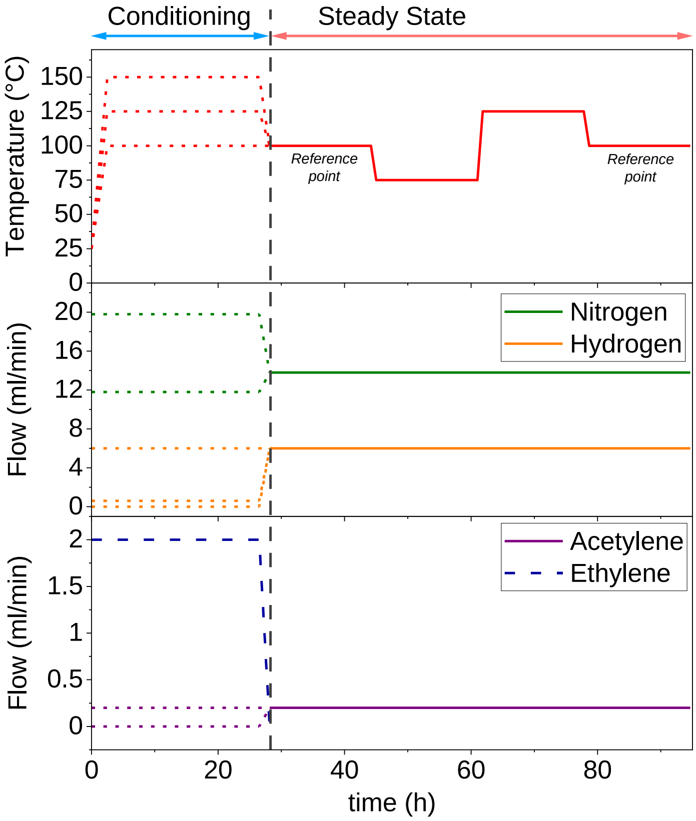

Supplementary Fig. 33 | Graphical representation of the measurement protocol with conditioning phase (dashed line) and steady state phase (solid line)

Considered experimental control factors

For each stage of catalyst preparation, we selected experimental control factors that might influence catalytic properties. The set of deposition and chemical synthesis control factors results in a 10-dimensional design of experiment (DoE) factor space. To systematically assess the influence of each control factor an efficient and robust screening design was computationally constructed. Maximally feasible ranges for each control factor are thereby employed. Not only does this probe their effects over the accessible synthesis space, it also improves the signal over experimental noise ratio.

Stage 1: SiO_x barrier layer deposition

- A: N_2O-Flow (3 levels: 0, **50**, or 100 sccm)
- B: Plasma Power (3 levels: 30, **115**, or 200 W)
- C: Total Pressure (3 levels: 0.3, **1.15**, or 2.0 µBar)

Stage 2: Pd Deposition

- D: Total Pressure 2 (3 levels: 4, **10**, or 16 µBar)
- E: Sputter Power (3 levels: 10, **35**, or 60 W)

The stage 1 and 2 factors will have different effects on the growing of layers. Varying the N_2O flow at constant monosilane flow rate (A) will change the stoichiometry of the growing film ranging from amorphous, hydrogen rich silicon (N_2O level of 0sccm), sub-stoichiometric SiO_x with excess silicon for a N_2O flow of 50sccm, up to SiO_2 for a N_2O flowrate of 100sccm. The plasma power during PECVD deposition (B) will directly correlate with the layer thickness and hydrogen content for Si-rich films. Highest thickness with lowest hydrogen content can be achieved for a RF power of 200W, due to highest SiH_4 dissociation rate. However, for a controllable growth within the desired thickness range, a RF power of 30W is usually chosen as a trade-off between hydrogen content and controllable film thickness. Finally, the total pressure (C and D) will change the film density, lateral homogeneity of the film as well as mechanical strain of the growing layer for both, barrier layer and catalyst deposition. Reference SiO_2 films are grown at a pressure of 0.3 mBar, for higher levels of the total pressure (1 – 2 mBar) a tendency towards pre-strained SiO_2 films is accepted. For sputtered Pd films, increasing the pressure (D) will lead to a less dense film compared to the reference, prone to reorganization during subsequent catalytic testing. Finally, increasing RF sputter power (E) will lead to increasing Pd film thickness, and less incorporation of impurities due to less contact time of the growing film with the sputter-chamber's atmosphere

The unique combinations of settings A-E in **Supplementary Table 9** also determine the number of thin films that needed to be produced. From each produced thin film sample, multiple smaller samples could be obtained and subjected to different conditioning phases followed by catalytic measurement.

Variations in catalytic observables during the initial TOS are indicative of catalyst formation. To gain control over such processes, the synthetic conditioning stage 3 was deliberately introduced (see **Supplementary Fig. 32 (i)**) within the reactor as an additional catalyst design step. Conditions applied at this stage can vary significantly from regular TOS conditions and should target long-term stability objectives rather than regular catalytic conversion.

Stage 3: Conditioning phase (24 h in reactor before catalytic testing)

- F: Final Temperature (3 levels: 100, **125** or 150°C)
- G: Heating rate (3 levels: 0.5, **2.75** or 5 K/min)
- H: Hydrogen (2 levels: 0, 6 ml/min)
- I: Carbon Source (3 levels for optimal design construction: Acetylene 0.2 / Ethylene 0, Acetylene 0.2 / Ethylene 2, or Acetylene 0 / Ethylene 2 ml/min)

For analysis finally split into:

- I.1: Acetylene (2 levels: 0 or 0.2 ml/min)
- I.2: Ethylene (2 levels: 0 or 2 ml/min)

During conditioning the catalyst's reactive interface forms and the presence or absence of carbon sources ("Acetylene", "Ethylene") or "Hydrogen" in the gas feed allows for an interface formation under different chemical potentials. The influence of thermal effects on this is addressed by varying initial "Heating rates" and the "Final (holding) temperatures" (over 24 hours).

Analyzing the influence of experimental results on catalytic stability

In summary, we arrive at 10 (considering I.1 and I.2) experimental factors whose influence on the catalytic properties were assessed. An optimal experimental design (a measurement plan, see **Supplementary Table 9**) over these factors was first constructed, as fully described below.

After measurements were completed, the influential factors can be identified from the following linear model for $\bar{S}_{2,i} - \bar{S}_{1,i}$ (for an experiment i)

$\bar{S}_{2,i} - \bar{S}_{1,i}$ = $\hat{\beta}_0 + \hat{\beta}_{\mathrm{A}} A_i + \hat{\beta}_{\mathrm{B}} B_i + \hat{\beta}_C C_i + \hat{\beta}_D D_i + + \hat{\beta}_E E_i + \hat{\beta}_F F_i + \hat{\beta}_G G_i + \hat{\beta}_H H_i + \hat{\beta}_{I.1} I.1_i + \hat{\beta}_{I.2} I.2_i$The estimated model coefficients $\hat{\beta}_X$ (X = A-I.2), when characterized as large in magnitude, then indicate strong influence from changes to the respective factor setting A_i-$I.2_i$. The vector of estimated model coefficients $\hat{\boldsymbol{\beta}}$ is here obtained from the feasible generalized least squares (GLS) estimator

$$\hat{\boldsymbol{\beta}} = \left(\mathbf{X}'\hat{\mathbf{V}}^{-1}\mathbf{X}\right)^{-1}\mathbf{X}'\hat{\mathbf{V}}^{-1}\mathbf{y}$$

- The vector $\mathbf{y}$ contains the response measured for each experiment i, here $\bar{S}_{2,i} - \bar{S}_{1,i}$.
- $\mathbf{X}$ is the design matrix for the linear model. It collects in columns the constant intercept and the factor settings A_i-$I.2_i$ as "fixed-effects". Note, that A_i-$I.2_i$ entered into the design matrix, under the following intuitive coding convention (also used in **Supplementary Table 9**, and any analysis is reported with respect to it). All factors were rescaled such that low and high levels are coded as -1 to 1. For control factors assessed on 3 levels, the 0 level then identifies the well-defined middle setting. Control factors assessed on only 2 levels pertain to the absence or presence (at an indicated flow setting) of gases in the conditioning feed. Albeit the tentative middle flow setting hasn't been experimentally realized we also treat these factors as continuous variables. Overall, these (tentative) 0 levels of each factor then serve as a reference setting: The estimated intercept coefficient describes the mean response expectable at this reference setting. A positive (negative) estimated model coefficient for a linear term then indicates an increase (decrease) in the modeled response when changing this factor's setting to a higher value.
- $\hat{\mathbf{V}}$ is an estimated variance-covariance matrix obtained by restricted maximum likelihood (REML) estimation. It accounts for the special noise structure in the experiment, e.g., arising from the batch requirements of the experiments carried out in the parallel reactor setup, see below.

Identifying influential second order terms

In our experimental design, we already took precautions to keep the estimated coefficients in the linear model free from bias by influential second order effects not included in the model (see below). Mitigating such "aliasing" then in principle allows us to detect true linear effects. Yet, additional conclusions can be drawn from the occurrence of such second order effects and we attempted an identification of a few decisive ones. Detection of related strong effects might be useful for the following reasons (effectively going from $\mathbf{X}$ to an extended model design matrix $\mathbf{X}_{\text{ext}}$):

1) A reduced error in modeling $\mathbf{y}$ might provide for a better estimate of the true experimental noise contribution. This potentially also reduces the confidence intervals around the linear coefficient estimates and consequently increases our certainty about their statistical (in-) significance. We briefly note, that the confidence intervals indeed narrowed when moving from $\mathbf{X}$ to $\mathbf{X}_{\text{ext}}$, while the assignment of statistical significance to main effects (at $p < 0.05$) remained unaffected. Similarly, the derived main effects remained qualitatively unaffected.
2) Identification of higher-order effects also builds a strong case in favor of optimally selected follow-up experiments to systematically unravel the complexity of the underlying response surface. This approach then seems favorable over one-factor at a time experimentation.

Hence, $\mathbf{X}$ is subsequently extended to $\mathbf{X}_{\text{ext}}$ by selected strong second order effects. Considered candidate terms included pairwise interactions of the form $\hat{\beta}_{X\,Y}X_iY_i$ and quadratic terms of the form $\hat{\beta}_{XX}X_i^2$. Our model selection approach to identify the important ones follows a rudimentary bottom-up strategy, here favored over the common top-down approaches for linear mixed models[56]. Using forward selection, we tested all possible model extensions by a single second term and in each round added the one with the lowest *p*-value. In two steps, this revealed two additional pairwise interactions with a highly significant contribution (p < 0.001). Finally, this led to the extended model.

$$\bar{S}_{2,i} - \bar{S}_{1,i} = \hat{\beta}_0 + \hat{\beta}_{\text{A}}\,A_i + \hat{\beta}_{\text{B}}\,B_i + \hat{\beta}_C\,C_i + \hat{\beta}_D\,D_i + + \hat{\beta}_E\,E_i + \hat{\beta}_F\,F_i + \hat{\beta}_G\,G_i + \hat{\beta}_H\,H_i + \hat{\beta}_{I.1}I.1_i + \hat{\beta}_{I.2}I.2_i + \hat{\beta}_{\text{E H}}E_iH_i + \hat{\beta}_{\text{E I.1}}E_iI.1_i$$

An additionally selected third interaction term (A x E) was significant at $p < 0.05$. While this term could be included next, we stopped at this stage, leaving a more detailed modeling of the response surface for further studies.

All models were constructed using the *lmer* function of the R package *lme4* (v1.1-33)[57] as interfaced from the python package *pymer4* (v0.8.0)[58]. In forward model selection of second order terms the Bayesian Information Criterion (BIC) was used to find the best candidate. Coefficient estimates and confidence intervals for the final model used as reported. The significance of coefficient estimates was assessed from the *p*-values computed by *lmerTest* (v3.1-3)[59] under Satterthwaite's degree of freedom method. The conditional and marginal coefficients of determination R_c^2 =0.81 and R_m^2 =0.74 [60] for the model fit were computed using the *rsquared* function of the R package *piecewiseSEM* (v2.3.0)[61].

Noise structure of the experiment

In the catalytic experiments carried out in the parallel reactor setup, 7 samples are measured simultaneously. Up to 7 different preparation phases (Factors A-E) can hence be assessed in one run. Yet, each sample is receiving a similar (nominally identical) feed and is kept at a similar temperature (Factors F-I). A certain commonality among the 7 samples might thus be expected from the similar conditioning phases applied to them. In consequence we considered this to be a special type of a blocked experiment – a split-plot experiment.

In such experiments, the assumption of a purely random experimental noise σ^2 might be violated and e.g., a run-to-run variance σ_b^2 might additionally contribute[62]. This “random-effects” structure is accounted for by the matrix $\widehat{\boldsymbol{V}}$. More specifically, we here allow for random shifts around the intercept that can arise from run-to-run variation between parallel reactor runs as well as by slight differences among the reactors 1-7. Apart from $\widehat{\boldsymbol{\beta}}$, these random effects were thus also estimated from the recorded response data. The obtained random effect estimates indicate a minor run-to-run variation in $\overline{S}_2 - \overline{S}_1$ (in %) among parallel reactor runs and a likely negligible one due to systematic variations among reactors.

Contribution	Type	Variance	Standard Deviation
Run	Random intercept	7.80	2.80
Reactor	Random intercept	0.37	0.61
Residual		25.92	5.10

Optimal construction of the robust experimental screening design

Considering the split-plot requirement, we constructed an experimental design that allows us to estimate the influence of experimental control factors on the catalytic performance. With an anticipated budget below 15 runs we decided to construct our initial experimental design from 10 runs. Most resources are then allocated to arrive at a high statistical power for effect estimation, while some follow-up experimentation was possible.

Literature designs likely do not fulfill all of our requirements. An algorithmic approach was therefore used to arrive at an optimal design that fulfils the specific requirements. We ultimately strive for a low uncertainty and a low bias in the estimated fixed effects (obtained from model coefficients in $\widehat{\boldsymbol{\beta}}$) due to aliasing. The latter objective reduces the misleading conclusions about influential design factors, see below. Both objectives are thus accounted for in the design construction through the following criteria:

- D-optimal designs afford a low variance of parameter estimates in $\widehat{\boldsymbol{\beta}}$ and can be constructed by maximizing the determinant of the Information Matrix $|\mathbf{X}'\mathbf{V}^{-1}\mathbf{X}|$. Here, 9 main effects A-I and an intercept column entered in the design matrix $\mathbf{X}$ (we initially considered a combined factor I instead of I.1 *and I.2*, see above). Purely random experimental noise σ^2 and a run-to-run variance σ_b^2 were initially considered in the construction of $\mathbf{V}$. Note however, that values of σ_b^2 and σ^2 were unknown at this point, but the introduction of a variance ratio among them suffices to carry out the optimization. A value of 1 has been assumed, constituting a robust choice when prior information is not available[63].

- To achieve a low bias in $\widehat{\boldsymbol{\beta}}$, we balance the former criterion with one that enhances the "Alias optimality" of our design[64] with respect to pairwise interaction terms not included in our model so far. In detail, the trace of the sum of squares of the alias matrix $\mathrm{trace}(\mathbf{A}'\mathbf{A})$ is minimized in the design construction phase (where $\mathbf{X}_2$ is the design matrix containing the pairwise interactions of A-I). The alias matrix is given by $\mathbf{A} = (\mathbf{X}'\mathbf{V}^{-1}\mathbf{X})^{-1}\mathbf{X}'\mathbf{V}^{-1}\mathbf{X}_2$ [65]. Simultaneously it was ensured, that the relative D-efficiency with respect to a D-optimal design remains above a certain threshold (using the default setting *minDopt*=0.8).

A point-exchange algorithm implemented in the R-package *skpr*[66] was used to find the optimal design under these criteria. The search was repeated 100 times to circumvent unfortuitous bias due to convergence to a certain local minimum. Note, that *skpr* thereby employs a robust two-step procedure to construct such optimal split-plot designs:

1. Focus is initially on Stage 3 factors F-I only. As their settings are reset less often, they exhibit lower statistical power compared to A-E. Hence, improving the optimality of settings for A-E in favor of F-I is likely an unreasonable choice. *Skpr* is thus used with above objectives (in this case $\mathbf{V} = \sigma^2\,\mathbf{I}$) to find an optimal outer "whole-plot" design for 10 runs of the parallel reactor setup. We thereby initially used candidates distributed over a design region $\chi = [-1,+1]^4$ over 3. For H only 2 levels (-1 and 1) were finally populated by the search. Note also, that for an intuitive analysis we slightly deviated from this factor initial coding scheme, see **Supplementary Table 9** and comments above.
2. After selecting this whole-plot design for F-I we proceeded to generate the inner "sub-plot" design, choosing factor settings A-E for the 7 samples of each run. The rows of a Box-Behnken (BB) Design[67] in 5 factors served as a candidate set.

After obtaining this ideal design for the estimation of main effects A-I, a practical choice was made to derive the final experimental design (**Supplementary Table 9**) from it: A set of 14 samples from the BB design were available to us at first. To start measurements, we selected runs 3 and 5 and replaced those 1 and 3 samples that were not yet available to us with (D-optimally selected) available ones. The quality of the experimental design remained practically unaffected by this exchange.

Limitations of the robust experimental screening design:

As described in detail above, influential control factors are identified from large (in magnitude) estimated linear model coefficients. Yet, "aliasing" can distort the conclusions that are drawn from such an analysis. Aliasing here refers to a situation where the estimated model coefficients are biased by active contributions from terms not included in the model. This can affect magnitude and sign of the estimated coefficients, and hence can mask influential factors or produce spurious ones.

To illustrate this, assume for a moment, that true coefficients for this model are summarized in $\boldsymbol{\beta}$ while the coefficients for the remaining unconsidered second order terms are given as $\boldsymbol{\beta}_2$. If such unconsidered second order terms are indeed important (and hence occur with a non-zero coefficient in $\boldsymbol{\beta}_2$), the estimated model coefficients $\widehat{\boldsymbol{\beta}}$ systematically deviate from the true coefficients $\boldsymbol{\beta}$ as:

$$E\{\widehat{\boldsymbol{\beta}}\} = \boldsymbol{\beta} + \mathbf{A}\boldsymbol{\beta}_2$$

where $\mathbf{A}$ is the alias matrix. A worst-case scenario would be encountered under strong aliasing (high respective values in $\mathbf{A}$) with highly influential unconsidered terms (high respective values in $\boldsymbol{\beta}_2$). Sign and magnitude of the coefficients are then altered so strongly, that important main effects might be missed, nonrelevant ones detected, or reversed signs for coefficients obtained (resulting in incorrectly recommended factor levels).

To mitigate this problem, we constructed our design such that aliasing with pairwise factor interactions is largely avoided. To a certain degree this allows us to detect true linear effects. For the model used in **Supplementary Fig. 32 (iv)**, we here analyze the remaining aliasing in the experimental screening design between linear and second order terms. The Alias matrix is visualized in **Supplementary Fig. 34** and was computed as $\mathbf{A} = \left(\mathbf{X}'\widehat{\mathbf{V}}^{-1}\mathbf{X}\right)^{-1}\mathbf{X}'\widehat{\mathbf{V}}^{-1}\mathbf{X}_2$ where $\boldsymbol{X}$ and $\mathbf{X}_2$ are the respective model design matrices.

Non surprisingly, only a small number of sizable terms are visible and our design exhibits a favorable aliasing structure for the screening of main effects. As the factor settings of “whole-plot” factors F-I.2 are reset less often in the design, some strong aliasing with terms including these factors is observed: The intercept is strongly aliased with the block of quadratic terms $(\text{F-I.2})^2$ and with the pairwise interaction term Ethylene x Acetylene. Aliasing is also observed for terms involving Acetylene (I.1) and Ethylene (I.2), previously considered as a combined factor I. Especially for influential factors such aliasing could be resolved in follow-up experiments.

Moving on, we also note, that the parallel reactor run might not be the only grouping variable. Others might include the respective reactor 1-7, the deposited film the catalytic sample was taken from or the grouping arising from batch processing (**Supplementary Table 9**). These groupings were not accounted for in the design construction phase.

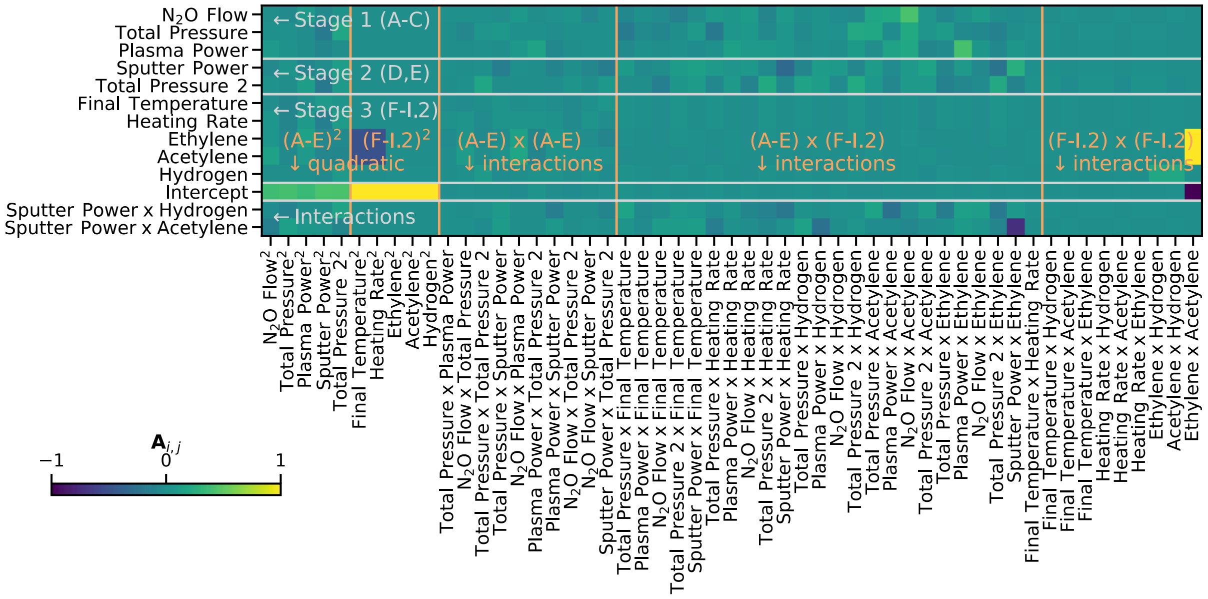

Supplementary Fig. 34 | Alias matrix for the model of **Supplementary Fig. 32 (iv)** with unconsidered second order effects (pairwise interaction terms and quadratic terms).

Supplementary References

1 Studt, F. *et al.* Identification of non-precious metal alloy catalysts for selective hydrogenation of acetylene. *Science* **320**, 1320-1322 (2008) doi:10.1126/science.1156660.

2 Studt, F. *et al.* On the role of surface modifications of palladium catalysts in the selective hydrogenation of acetylene. *Angew. Chem. Int. Ed.* **120**, 9439-9442 (2008) doi:10.1002/ange.200802844.

3 Rose, M. K., Borg, A., Mitsui, T., Ogletree, D. F. & Salmeron, M. Subsurface impurities in Pd(111) studied by scanning tunneling microscopy. *J. Chem. Phys.* **115**, 10927-10934 (2001) doi:10.1063/1.1420732.

4 Yang, B., Burch, R., Hardacre, C., Headdock, G. & Hu, P. Influence of surface structures, subsurface carbon and hydrogen, and surface alloying on the activity and selectivity of acetylene hydrogenation on Pd surfaces: a density functional theory study. *J. Catal.* **305**, 264-276 (2013) doi:10.1016/j.jcat.2013.05.027.

5 Clark, I. T., Yoshimura, M. & Ueda, K. Palladium thin-films on clean and hydrogen-terminated Si(110): the effect of hydrogen termination on metal adsorption. *Jpn. J. Appl. Phys.* **48**, 08JB04 (2009) doi:10.1143/jjap.48.08jb04.

6 Yamada, Y. M. A., Baek, H., Sato, T., Nakao, A. & Uozumi, Y. Metallically gradated silicon nanowire and palladium nanoparticle composites as robust hydrogenation catalysts. *Commun. Chem.* **3**, 81 (2020) doi:10.1038/s42004-020-0332-z.

7 Teschner, D. *et al.* Alkyne hydrogenation over Pd catalysts: a new paradigm. *J. Catal.* **242**, 26-37 (2006) doi:10.1016/j.jcat.2006.05.030.

8 Alay, J. L. & Hirose, M. The valence band alignment at ultrathin SiO_2/Si interfaces. *J. Appl. Phys.* **81**, 1606-1608 (1997) doi:10.1063/1.363895.

9 Ichinohe, T., Masaki, S., Kawasaki, K. & Morisaki, H. Palladium silicide oxide formations in Pd/SiO_2 complex films. *Thin Solid Films* **343**, 119-122 (1999) doi:10.1016/S0040-6090(98)01626-5.

10 Ho, P. S. & Rubloff, G. W. Electronic states and microstructure at the silicide-silicon interface. *Thin Solid Films* **89**, 433-446 (1982) doi:10.1016/0040-6090(82)90324-8.

11 Tew, M. W., Miller, J. T. & van Bokhoven, J. A. Particle size effect of hydride formation and surface hydrogen adsorption of nanosized palladium catalysts: L_3 edge vs K edge X-ray absorption spectroscopy. *J. Phys. Chem. C* **113**, 15140-15147 (2009) doi:10.1021/jp902542f.

12 Bancroft, G. M. *et al.* Toward a comprehensive understanding of solid-state core-level XPS linewidths: experimental and theoretical studies on the Si 2p and O 1s linewidths in silicates. *Phys. Rev. B* **80**, 075405 (2009) doi:10.1103/PhysRevB.80.075405.

13 Gabasch, H. *et al.* In situ XPS study of Pd(111) oxidation at elevated pressure, Part 2: palladium oxidation in the 10^{-1} mbar range. *Surf. Sci.* **600**, 2980-2989 (2006) doi:10.1016/j.susc.2006.05.029.

14 Zemlyanov, D. *et al.* In situ XPS study of Pd(111) oxidation. Part 1: 2D oxide formation in 10^{-3} mbar O_2. *Surf. Sci.* **600**, 983-994 (2006) doi:10.1016/j.susc.2005.12.020.

15 Davoli, I. *et al.* The local electronic structure of PdO crystal and PdO catalyst supported on SiO_2 and γ-Al_2O_3 from L_3 and L_1 x-ray absorption Pd edge in XANES spectra. *Solid State Commun.* **48**, 475-478 (1983) doi:10.1016/0038-1098(83)90857-8.

16 Wu, T. P., Kaden, W. E., Kunkel, W. A. & Anderson, S. L. Size-dependent oxidation of Pd_n ($n \leqslant 13$) on alumina/NiAl(110): correlation with Pd core level binding energies. *Surf. Sci.* **603**, 2764-2770 (2009) doi:10.1016/j.susc.2009.07.014.
17 Sengar, S. K., Mehta, B. R. & Govind. Size and alloying induced changes in lattice constant, core, and valance band binding energy in Pd-Ag, Pd, and Ag nanoparticles: effect of in-flight sintering temperature. *J. Appl. Phys.* **112**, 014307 (2012) doi:10.1063/1.4731714.
18 Aruna, I., Mehta, B. R., Malhotra, L. K. & Shivaprasad, S. M. Size dependence of core and valence binding energies in Pd nanoparticles: interplay of quantum confinement and coordination reduction. *J. Appl. Phys.* **104**, 064308 (2008) doi:10.1063/1.2973682.
19 Zhang, J. *et al.* Composition of the green oil in hydrogenation of acetylene over a commercial Pd-Ag/Al_2O_3 Catalyst. *Chem. Eng. Technol.* **39**, 865-873 (2016) doi:10.1002/ceat.201600020.
20 Shirakawa, H., Ito, T. & Ikeda, S. Raman scattering and electronic spectra of poly(acetylene). *Polym. J.* **4**, 460-462 (1973) doi:10.1295/polymj.4.460.
21 Schaffer, H., Chance, R., Silbey, R., Knoll, K. & Schrock, R. Conjugation length dependence of Raman scattering in a series of linear polyenes: implications for polyacetylene. *J. Chem. Phys.* **94**, 4161-4170 (1991) doi:10.1063/1.460649.
22 Belov, D., Ol'khov, Y. A., Belov, G., Solovyeva, T. & Kozub, G. Thermal study of irradiated polyacetylene films. *J. Therm. Anal. Calorim.* **46**, 237-243 (1996) doi:10.1007/BF01979964.
23 Armbrüster, M. *et al.* Pd−Ga intermetallic compounds as highly selective semihydrogenation catalysts. *J. Am. Chem. Soc.* **132**, 14745-14747 (2010) doi:10.1021/ja106568t.
24 Ellis, I. T. *et al.* Lithium and boron as interstitial palladium dopants for catalytic partial hydrogenation of acetylene. *Chem. Commun.* **53**, 601-604 (2017) doi:10.1039/c6cc08404d.
25 He, Y. *et al.* Pd nanoparticles on hydrotalcite as an efficient catalyst for partial hydrogenation of acetylene: effect of support acidic and basic properties. *J. Catal.* **331**, 118-127 (2015) doi:10.1016/j.jcat.2015.08.012.
26 Luo, Y. *et al.* Addressing electronic effects in the semi-hydrogenation of ethyne by $InPd_2$ and intermetallic Ga–Pd compounds. *J. Catal.* **338**, 265-272 (2016) doi:10.1016/j.jcat.2016.03.025.
27 Shao, L. *et al.* Nanosizing intermetallic compounds onto carbon nanotubes: active and selective hydrogenation catalysts. *Angew. Chem. Int. Ed.* **50**, 10231-10235 (2011) doi:10.1002/anie.201008013.
28 Liu, Y., He, Y., Zhou, D., Feng, J. & Li, D. Catalytic performance of Pd-promoted Cu hydrotalcite-derived catalysts in partial hydrogenation of acetylene: effect of Pd–Cu alloy formation. *Catal. Sci. Technol.* **6**, 3027-3037 (2016) doi:10.1039/c5cy01516b.
29 Armbrüster, M., Wowsnick, G., Friedrich, M., Heggen, M. & Cardoso-Gil, R. Synthesis and catalytic properties of nanoparticulate intermetallic Ga–Pd compounds. *J. Am. Chem. Soc.* **133**, 9112-9118 (2011) doi:10.1021/ja202869d.
30 Osswald, J. *et al.* Palladium–gallium intermetallic compounds for the selective hydrogenation of acetylene. Part II: surface characterization and catalytic performance. *J. Catal.* **258**, 219-227 (2008) doi:10.1016/j.jcat.2008.06.014.

31 Wei, S. *et al.* Direct observation of noble metal nanoparticles transforming to thermally stable single atoms. *Nat. Nanotechnol.* **13**, 856-861 (2018) doi:10.1038/s41565-018-0197-9.
32 Pei, G. X. *et al.* Promotional effect of Pd single atoms on Au nanoparticles supported on silica for the selective hydrogenation of acetylene in excess ethylene. *New J. Chem.* **38**, 2043-2051 (2014) doi:10.1039/c3nj01136d.
33 Huang, F. *et al.* Atomically dispersed Pd on nanodiamond/graphene hybrid for selective hydrogenation of acetylene. *J. Am. Chem. Soc.* **140**, 13142-13146 (2018) doi:10.1021/jacs.8b07476.
34 Yudanov, I. V. *et al.* CO adsorption on Pd nanoparticles: density functional and vibrational spectroscopy studies. *J. Phys. Chem. B.* **107**, 255-264 (2003) doi:10.1021/jp022052b.
35 Bradshaw, A. & Hoffmann, F. The chemisorption of carbon monoxide on palladium single crystal surfaces: IR spectroscopic evidence for localised site adsorption. *Surf. Sci.* **72**, 513-535 (1978) doi:10.1016/0039-6028(78)90367-9.
36 Li, X. A., Sudarsanam, N. & Frey, D. D. Regularities in data from factorial experiments. *Complexity* **11**, 32-45 (2006) doi:10.1002/cplx.20123.
37 Decrescenzi, M. *et al.* X-ray absorption near-edge structure and extended X-ray absorption fine-structure investigation of Pd silicides. *Phys. Rev. B* **32**, 612-622 (1985) doi:10.1103/PhysRevB.32.612.
38 Zhou, H. *et al.* PdZn intermetallic nanostructure with Pd–Zn–Pd ensembles for highly active and chemoselective semi-hydrogenation of acetylene. *ACS Catal.* **6**, 1054-1061 (2016) doi:10.1021/acscatal.5b01933.
39 Ota, A. *et al.* Intermetallic compound Pd_2Ga as a selective catalyst for the semi-hydrogenation of acetylene: from model to high performance systems. *J. Phys. Chem. C* **115**, 1368-1374 (2011) doi:10.1021/jp109226r.
40 Cao, Y., Sui, Z., Zhu, Y., Zhou, X. & Chen, D. Selective hydrogenation of acetylene over Pd-In/Al_2O_3 catalyst: promotional effect of indium and composition-dependent performance. *ACS Catal.* **7**, 7835-7846 (2017) doi:10.1021/acscatal.7b01745.
41 Tao, X. *et al.* Highly active isolated single-atom Pd catalyst supported on layered MgO for semihydrogenation of acetylene. *ACS Appl. Energy Mater.* **5**, 10385-10390 (2022) doi:10.1021/acsaem.2c02076.
42 Pei, G. X. *et al.* Ag alloyed Pd single-atom catalysts for efficient selective hydrogenation of acetylene to ethylene in excess ethylene. *ACS Catal.* **5**, 3717-3725 (2015) doi:10.1021/acscatal.5b00700.
43 Xu, X. *et al.* Thermal effect optimization endows a selective and stable PdCu single atom alloy catalyst for acetylene hydrogenation. *AIChE J.* **69** (2023) doi:10.1002/aic.18042.
44 Liu, Y. *et al.* Polyoxometalate-based metal–organic framework as molecular sieve for highly selective semi-hydrogenation of acetylene on isolated single Pd atom sites. *Angew. Chem. Int. Ed.* **60**, 22522-22528 (2021) doi:10.1002/anie.202109538.
45 Zhong, M. *et al.* Depositing Pd on the outmost surface of Pd_1Ni/SiO_2 single-atom alloy via atomic layer deposition for selective hydrogenation of acetylene. *Appl. Catal. A Gen.* **662**, 119288 (2023) doi:10.1016/j.apcata.2023.119288.
46 Li, R. *et al.* Selective hydrogenation of acetylene over Pd-Sn catalyst: identification of Pd_2Sn intermetallic alloy and crystal plane-dependent performance. *Appl. Catal. B* **279**, 119348 (2020) doi:10.1016/j.apcatb.2020.119348.

47 Li, Z. *et al.* Regulating metal-support interactions of Pd/$MgAl_2O_4$ for efficient selective hydrogenation of acetylene. *Catal. Today* **423**, 114253 (2023) doi:10.1016/j.cattod.2023.114253.
48 Forman, A. J. *et al.* Silica-encapsulated Pd nanoparticles as a regenerable and sintering-resistant catalyst. *ChemCatChem* **2**, 1318-1324 (2010) doi:10.1002/cctc.201000015.
49 Zou, S. *et al.* Grafting nanometer metal/oxide interface towards enhanced low-temperature acetylene semi-hydrogenation. *Nat. Commun.* **12**, 5770 (2021) doi:10.1038/s41467-021-25984-8.
50 Guo, Y. *et al.* Photo-thermo semi-hydrogenation of acetylene on Pd_1/TiO_2 single-atom catalyst. *Nat. Commun.* **13**, 2648 (2022) doi:10.1038/s41467-022-30291-x.
51 Huang, F. *et al.* Low-temperature acetylene semi-hydrogenation over the Pd_1–Cu_1 dual-atom catalyst. *J. Am. Chem. Soc.* **144**, 18485-18493 (2022) doi:10.1021/jacs.2c07208.
52 Armbrüster, M. *et al.* $Al_{13}Fe_4$ as a low-cost alternative for palladium in heterogeneous hydrogenation. *Nat. Mater.* **11**, 690-693 (2012) doi:10.1038/nmat3347.
53 Gao, R. *et al.* Pd/Fe_2O_3 with electronic coupling single-site Pd–Fe pair sites for low-temperature semihydrogenation of alkynes. *J. Am. Chem. Soc.* **144**, 573-581 (2022) doi:10.1021/jacs.1c11740.
54 Liu, Y. *et al.* Palladium phosphide nanoparticles as highly selective catalysts for the selective hydrogenation of acetylene. *J. Catal.* **364**, 406-414 (2018) doi:10.1016/j.jcat.2018.06.001.
55 Feng, Q. *et al.* Isolated single-atom Pd sites in intermetallic nanostructures: high catalytic selectivity for semihydrogenation of alkynes. *J. Am. Chem. Soc.* **139**, 7294-7301 (2017) doi:10.1021/jacs.7b01471.
56 Zuur, A. F., Ieno, E. N., Walker, N. J., Saveliev, A. A. & Smith, G. M. *Mixed effects models and extensions in ecology with R*. Vol. 574 (Springer, 2009).
57 Bates, D., Machler, M., Bolker, B. M. & Walker, S. C. Fitting linear mixed-effects models using lme4. *J. Stat. Softw.* **67**, 1-48 (2015) doi:10.18637/jss.v067.i01.
58 Jolly, E. Pymer4: connecting R and Python for linear mixed modeling. *J. Open Source Softw.* **3**, 862 (2018) doi:10.21105/joss.00862.
59 Kuznetsova, A., Brockhoff, P. B. & Christensen, R. H. lmerTest package: tests in linear mixed effects models. *J. Stat. Softw.* **82**, 1-26 (2017) doi:10.18637/jss.v082.i13.
60 Nakagawa, S. & Schielzeth, H. A general and simple method for obtaining R2 from generalized linear mixed-effects models. *Methods Ecol. Evol.* **4**, 133-142 (2013) doi:10.1111/j.2041-210x.2012.00261.x.
61 Lefcheck, J. S. PIECEWISESEM: Piecewise structural equation modelling in R for ecology, evolution, and systematics. *Methods Ecol. Evol.* **7**, 573-579 (2016) doi:10.1111/2041-210x.12512.
62 Jones, B. & Nachtsheim, C. J. Split-plot designs: what, why, and how. *J. Qual. Technol.* **41**, 340-361 (2009) doi:10.1080/00224065.2009.11917790.
63 Goos, P. *The optimal design of blocked and split-plot experiments*. Vol. 164 (Springer Science & Business Media, 2012).
64 Jones, B. & Nachtsheim, C. J. Efficient designs with minimal aliasing. *Technometrics* **53**, 62-71 (2011) doi:10.1198/Tech.2010.09113.
65 Kulahci, M. & Bisgaard, S. A generalization of the alias matrix. *J. Appl. Stat.* **33**, 387-395 (2006) doi:10.1080/02664760500449014.

66 Morgan-Wall, T. & Khoury, G. Optimal design generation and power evaluation in R: the skpr Package. *J. Stat. Softw.* **99**, 1-36 (2021) doi:10.18637/jss.v099.i01.

67 Wei, X., Ma, Z., Lu, J., Mu, X. & Hu, B. The highly efficient and selective dicarbonylation of acetylene catalysed by palladium nanosheets supported on activated carbon. *New J. Chem.* **44**, 11835-11840 (2020) doi:10.1039/d0nj01173h.
